# Enduring constraints on grammar revealed by Bayesian spatiophylogenetic analyses

**DOI:** 10.1038/s41562-025-02325-z

**Published:** 2025-11-17

**Authors:** Annemarie Verkerk, Olena Shcherbakova, Hannah J. Haynie, Hedvig Skirgård, Christoph Rzymski, Quentin D. Atkinson, Simon J. Greenhill, Russell D. Gray

**Affiliations:** 1 Department of Language Science and Technology, Saarland University, Saarbrücken, Germany; 2 Department of Linguistic and Cultural Evolution, Max Planck Institute for Evolutionary Anthropology, Leipzig, Germany; 3 Department of Linguistics, University of Colorado Boulder, Boulder, CO USA; 4 School of Psychology, University of Auckland, Auckland, New Zealand; 5 School of Anthropology and Museum Ethnography, University of Oxford, Oxford, UK; 6 School of Biological Sciences, University of Auckland, Auckland, New Zealand

**Keywords:** Evolution of language, Language and linguistics, Phylogenetics

## Abstract

Human languages show astonishing variety, yet their diversity is constrained by recurring patterns. Linguists have long argued over the extent and causes of these grammatical ‘universals’. Using Grambank—a comprehensive database of grammatical features across the world’s languages—we tested 191 proposed universals with Bayesian analyses that account for both genealogical descent and geographical proximity. We find statistical support for about a third of the proposed linguistic universals. The majority of these concern word order and hierarchical universals: two types that have featured prominently in earlier work. Evolutionary analyses show that languages tend to change in ways that converge on these preferred patterns. This suggests that, despite the vast design space of possible grammars, languages do not evolve entirely at random. Shared cognitive and communicative pressures repeatedly push languages towards similar solutions.

## Abstract (web summary)

Despite their great diversity, human languages are shaped by recurring grammatical universals. Verkerk et al. show that about one-third of the proposed universals hold cross-linguistically through analyses of the Grambank database.

## Main

Human languages are strikingly diverse. They vary in almost every way^1^, from the sounds they use to the ordering of words and other grammar rules. However, such diversity does not preclude the existence of regular patterns and structured variation. A central goal of linguistics has been to describe the patterns and structure of human linguistic diversity and identify the constraints on that diversity^2–8^. In Hjelmslev’s^2^ words, the aim of linguistic typology/theory “must be to show which structures are possible, in general, and why it is just those structures, and not others, that are possible”.

Some argue that languages all face the same pressures for communicating and encoding information, leading to convergence towards structurally good solutions^9–11^. For example, take verb agreement, the phenomenon where verbs are marked for the person and number features of their grammatical arguments, such as ‘-es’ in ‘Alex catch-es the ball’. Verb agreement in English is very limited; in other languages, it is far more extensive or non-existent altogether. One explanation for this cross-linguistic variation is that verb agreement ‘trades off’ with word order. Speakers of all languages need a way to differentiate argument relations (to identify and mark subjects, objects and other syntactic arguments as such), and languages with subject–verb–object word order (as in English) do not ‘need’ verb agreement because subjects are on one side of the verb and objects on the other.

Others argue that all languages are shaped by our human cognitive capacity for online production, comprehension and acquisition^5,12–22^. Word order patterns, especially those related to the order of object and verb, have been explained in terms of efficient online processing. These have been claimed to be rooted in principles where the order of the ‘head’ (the most important element of a phrase that determines its type and syntactic behaviour) and its ‘dependents’ (other elements in the phrase) match each other across different types of phrases^13,17,23^. For example, if adpositions (heads of adpositional phrases) precede nouns (dependents in adpositional phrases) in a given language, we may expect the same ‘matched’ pattern where verbs (heads of verb phrases) also precede objects (dependents in verb phrases).

It is also possible that all languages are shaped by general pathways of diachronic language change^11,24–28^. For example, the association between the order of adposition and noun and the order of object and verb has been explained through the common process of grammaticalization where adpositions develop from verbs^29^. Here, adpositions such as ‘for’ may arise from verbs meaning ‘give’ (as in ‘Anna gave John a flower’), and if the word order is such that verbs come before objects, then these forms will be prepositions rather than postpositions (‘for John’ rather than ‘John for’).

The nature of universals and the extent to which the types of constraints mentioned above contribute to their emergence is of direct relevance for understanding the nature of human language and human cognition and, hence, a matter of some dispute across various approaches to linguistics^30,31^. Within formal approaches, universals such as those invoked in X-bar structure (simplistically, the idea that phrases of any type consist of specifiers, heads and their complements^32,33^) are seen as absolute rules of human language, tied to robust, innate grammatical constraints^5,7,8,32,34^. Evans and Levinson^1^ argue against absolute universals of all types, emphasizing the diversity of the world’s languages and the complex interactions of multiple potential constraints in any non-trivial generalizations about human grammars. Generative replies to Evans and Levinson^1^, such as Freidin^35^, Pesetsky^36^ and Rizzi^37^, argue that their account does not hold, among other critiques, because the generative level of analysis is deep. In contrast, the analysis in Evans and Levinson^1^ and other work in functional typology remains surface-level. Hence, the generative study of universals is distinct from the typological one, both in its methods and in terms of explanations.

The current study is rooted in the field of linguistic typology, initially pioneered by Greenberg^38^, which has subsequently generated a large body of research on linguistic universals (see, among other works, ref. ^6^). This approach focuses on identifying patterns of grammatical feature co-occurrence that need not be exceptionless (‘statistical universals’), in many cases using them to inform theories that invoke the aforementioned types of constraints (communication, cognition and language change). For example, Greenberg’s^38^ universal number 4 claims that “with overwhelmingly greater than chance frequency, languages with normal SOV order are postpositional” (where SOV is subject–object–verb order), which follows the earlier example. Dryer^23^ finds evidence for a large set of word order associations using a large language sample and proposes an explanation for them rooted in ease of online language processing. Similarly, Bickel et al.^39^ have demonstrated that a strong cognitive preference to identify the first base-form noun phrase as the agent (the ‘do-er’ of an action) leads to a persistent bias against so-called ergative languages, which can explain the rarity of this pattern.

These types of explanations for universals are often grounded in the so-called competing motivations account, which can account for both variation across languages as well as provide (cognitive) grounds for the universal itself. A famous example of a formal application of this model is Aissen’s^40^ optimality theory account of differential object marking (DOM). DOM is common cross-linguistic behaviour where some direct objects in a language are marked (for example, with case or adpositions), while other direct objects remain unmarked. For example, in Spanish, direct objects that are definite and denote human referents are preceded by the marker ‘*a*’ (lit. ‘to’), whereas other objects are left unmarked. Aissen demonstrates that cross-linguistic variation in DOM can be explained by two interacting principles (motivations): (1) iconicity, which implies that more prominent objects (those that are definite, human or higher animates) are more likely to receive marking and (2) economy, which implies that marking should be avoided altogether. Her analysis suggests that these two constraints interact in such a way that the outcome is the observed cross-linguistic variation in differential object marking—in DOM languages, highly prominent objects always receive marking, but languages vary on the ‘cut-off’ regarding less prominent objects. Underlyingly, the iconicity motivation may be grounded in the communicative need to distinguish prominent objects from subjects, which also tend to be definite, human or higher animates.

Such accounts form the theoretical groundwork for why we may find universals in the first place. However, not all linguists are convinced of the importance of such constraints: Dunn et al.^41^ argue that statistical word order universals arise in “an evolutionary landscape with channels and basins of attraction that are specific to linguistic lineages”. They claim that word order correlations do not emerge in response to functional constraints, but rather are a consequence of particular diachronic changes unique to particular language families. A common view among generative linguists is that universal grammar does not (and should not aim to) explain statistical universals^34^.

Resolving the complex problem of what grammatical relationships are ‘universals’ and what they mean for understanding language or cognition has been hindered by several fundamental challenges. First, the lack of a comprehensive grammatical dataset has meant previous work has tended to consider a relatively small subset of the world’s ~7,000 languages, limiting statistical power and the ability to test for strong associations rigorously. Second, shared linguistic ancestry and the diffusion of features between neighbouring populations mean linguistic data do not constitute independent data points, violating the independence assumption of many statistical tests and potentially generating spurious statistical associations between features^42^. Finally, raw correlations between features (or traits) tell us little about the historical causal relationships between them.

Here, we overcome these challenges by analysing a comprehensive database of grammatical features, Grambank^43^, which covers more languages than previous work (Supplementary Text 1), and employing sophisticated methodologies to handle non-independence and test for co-evolution. We test 191 putative ‘linguistic universals’ extracted from the Universals Archive^44^ (Supplementary Text 2 and 3). These are all so-called implicational universals, as Greenberg’s^38^ universal number 4 above (SOV ⇒ postpositions): they relate characteristics of the world’s languages in an ‘if X, then Y’ structure. First, we apply a Bayesian generalized linear mixed effects model to evaluate the support for each hypothesis while controlling for genealogical and geographical relations. We then apply a Bayesian phylogenetic method to infer the underlying evolutionary dynamics (see Methods and Supplementary Text 5 for explanations and rationale). We see this work as rising to the challenge spelt out by Piantadosi and Gibson^45^, which claims that “claims about linguistic universals should be accompanied by some measure of the strength of evidence in favour of such a universal”. They propose that any hypothesized universal should be compared with the corresponding null hypothesis using a Bayes factor or similar, and point out that such measures are critical given the relevance of universals to debates on the nature of human language.

To examine differences in the reasoning behind universals we divided the generalizations from the Universals Archive into four types, reflecting recurrent themes in typological literature^10,12,46^: (1) narrow word order, (2) broad word order, (3) hierarchical universals and (4) other. Narrow word order universals link the order of words in two or more constructions in ways that generally relate to where the most important words occur, such as Greenberg’s^38^ universal 4 above. Broad word order universals correlate a word order feature with a morphosyntactic feature unrelated to word order, such as “non-accusative alignment may be associated with verb–initial order”^47^. Hierarchical universals are chains of implicational universals with the most frequently attested traits on the left and the rarest ones on the right, as defined within the same domain or paradigm. An example is Greenberg’s^38^ claim that “no language has a dual unless it has a plural”; this type of universal has also been called scalar or ‘scale’ in literature. The remaining universals are captured under ‘other’, but in practice often correlate two morphological features (see Methods and Supplementary Text 2, 7 and 8 for further details).

## Results

### Phylogenetic and spatial correlation

**Fig. 1:**
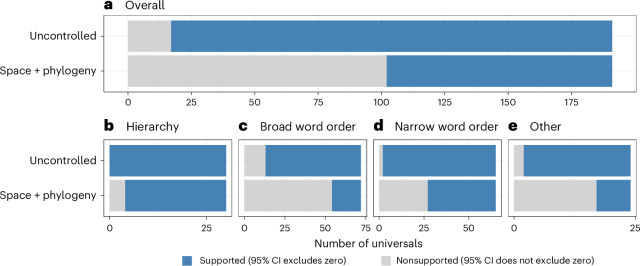
Bar chart showing the proportion of supported universals under the naive model and the spatiophylogenetic model. Support implies, for the naive model, that the 95% CI of posterior coefficient estimates does not straddle zero. For the spatiophylogenetic model (with genealogical and geographical relations controlled for), it means that the median of the 95% CI of the main fixed effect estimates does not straddle zero. Universals identified to be supported are coloured in blue, while non-supported universals are coloured in grey. **a**, The overall universals. **b**–**e**, The universals by subset: hierarchy (**b**), broad word order (**c**), narrow word order (**d**) and other (**e**).

We constructed Bayesian generalized linear mixed effects models (GLMMs) for all universals using brms^48^ implemented in R^49^. We find that, without controlling for genealogical and geographical relations, the vast majority of the proposed universals are supported by our regression models—that is, the fixed effect of the predictor variable (the second part of the universal) has posterior estimates whose 95% credible interval (CI) exclude zero (Fig. 1, Supplementary Data 1 and Supplementary Table 3). In these naive models, 174 (91%) of the 191 universals are found to be supported.

However, when we do control for spatial and phylogenetic non-independence this number decreases substantially to 89 of 191 (47%). Here, we conduct the analysis over 100 phylogenetic trees^50^ (Methods) and hence report on the median of posterior estimates and their 95% CIs (Supplementary Fig. 4, Supplementary Data 1 and Supplementary Table 4). We find marked differences between the strength of support for four types of universals (Fig. 1). There is strong support for hierarchical universals with 24 of 30 (80%) having posterior estimates that exclude zero. The narrow word order universals are also relatively well supported, with 36 of 65 (58%) confirmed. In contrast, there is weaker support for the broad word order (18 of 72 supported, 25%) and the ‘other’ universals (7 of 24, 32%).

### Evolutionary dynamics

**Fig. 2:**
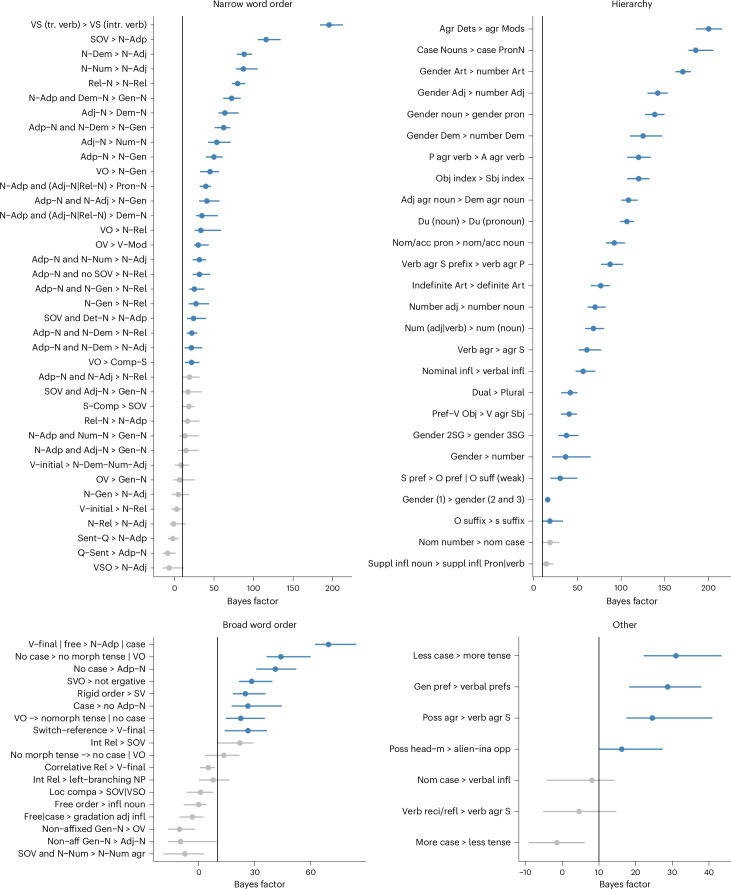
Median natural log BF and their 95% HDI from the BayesTraits analyses showing support for co-evolutionary models. Universals with the lower bound of the 95% HDI on the distribution of BF >10 are considered supported and are coloured blue. The relationships are ranked from strongest to weakest, top to bottom, per category. The universals are given in short form, where the formula X ⇒ Y means ‘if X, then Y’; the full citations, sources and all abbreviations can be found in Supplementary Table 2. Sample sizes can be found in Supplementary Data 1 and Supplementary Table 4.

To infer the evolutionary (in the sense of diachronic) pathways behind the statistically supported universals identified in the spatiophylogenetic brms analyses, we performed co-evolution analyses using the BayesTraits program^51,52^. Again, we conducted the analyses over 100 phylogenetic trees, calculated Bayes factors (BF) by comparing the dependent and independent model and calculated the 95% high density interval (HDI) to summarize BF support for each universal (Methods and Supplementary Texts 5 and 6). We took the lower bound of the 95% HDI >10 as indicating support for the dependent model of trait co-evolution over the independent model (Fig. 2, Supplementary Data 1 and Supplementary Table 4). On this criterion, 60 of the 89 universals supported in the spatiophylogenetic model were also supported in the co-evolution analyses. We continue discussing this set of 60 universals. Across the different types of universals, we observe the same pattern as for the spatiophylogenetic correlations: the strongest evidence can be found among the hierarchical universals (evidence for all of the 24 universals supported in the spatiophylogenetic analysis, 80% of all hierarchical universals (*n* = 30)). Second, the word order universals show a more mixed pattern: for the narrow word order universals, less than half of narrow word order universals (24/36, 37% in all (*n* = 65)) and a much smaller fraction of the broad word order universals (8/18, 11% in all (*n* = 72)) are supported. Third, we find that only four of the seven ‘other’ universals supported in the spatiophylogenetic model hold (17% in total, *n* = 24).

### Robustness

Since both studies are dependent on a global phylogeny of languages^50^ (Methods), we conducted additional tests with categorical control for language family. Our results also hold with this approach (Supplementary Texts 5 and 6). Of the 89 statistically supported universals identified in the spatiophylogenetic analysis using the global phylogeny, 67 (75%) are supported using a categorical control for language family instead (Supplementary Fig. 6, Supplementary Data 1 and Supplementary Table 3). The main effect estimates of the two studies (the spatiophylogenetic brms models and the categorical control for language family brms models) are highly correlated (Spearman’s *r* = 0.93 (189 degrees of freedom), *P* < 0.001, 95% CI 0.91–0.95).

Taking the universals supported in the spatiophylogenetic brms model and additionally also supported in the BayesTraits analyses, we find support for 60 (of 191, 31%) universals. The strength of the correlations between the two terms linked in the putative universal (median of the main effect estimates in brms and the median BF in BayesTraits) are high (Spearman’s *r* = 0.72 (189 degrees of freedom), *P* < 0.001, 95% CI 0.65–0.78). In the remainder of the paper, we will consider these 60 as well-supported universals and discuss them further. See Supplementary Text 5 for more on the comparison of these two analyses.

### An analysis of possible biases in past studies

The conditions under which the universals were first proposed were considered as possible explanatory factors of why some universals are well-supported in the spatiophylogenetic brms models and the BayesTraits analyses. Many of the strongest supported universals were formulated in the 1960s and 1970s, and the language samples then were often relatively small and biased (often featuring mainly Eurasian languages). We considered the size and geographical bias of the original language sample and implementation of the universal in terms of the Grambank questionnaire (Supplementary Text 9). We found that none of these had an impact on the support of universals as defined above. This shows that the limited representation of worldwide linguistic diversity in twentieth century research on universals did not prevent the discovery of universals that still hold in our study, with its massively increased sample size (Supplementary Text 1) and appropriate statistical methodology.

### Hierarchical universals

The tested hierarchical universals generally deal with the expression of grammatical categories through agreement, and within the (pro)nominal paradigm. An assessment of why certain universals are supported (and others are not) is provided in Supplementary Text 9. Here, we discuss that hierarchical universals have often been explained using competing motivations accounts, where ‘competition’ between certain principles (‘motivations’) such as economical behaviour (avoidance of overt grammatical marking) and salience (preferred marking of entities that matter, such as humans) results in the attested cross-linguistic distributions^9,53–55^. We think that the success of the studied hierarchical universals (80%, 24 of 30, are supported by both the spatiophylogenetic brms and BayesTraits analyses) can be explained in such an account, especially if we consider the role of diachronic change and if the universals are formulated in a specific enough manner: two unsupported ones are probably too general. Note that we do not and cannot test which explanations are ‘best’ in this paper, and that which follows (also on other types of universals) is left for further research. As is clear from Fig. 2 and Supplementary Figs. 6 and 7, there is a gradient in support for hierarchical universals despite them being highly supported as a category; this gradient persists across the different analyses and should be the focus of further investigation.

Many of the supported universals in this category posit the presence of a more common feature given the presence of a rarer feature within the same paradigm. Hierarchical universals hence capture the frequency at which various features appear in the world’s languages and language change in (morphological) paradigms. Exemplified in Supplementary Fig. 10 is Plank’s^56^ “If determiners agree within NPs, modifiers are likelier also to agree than not to agree”. Agreement within noun phrases (NPs) is common in the languages of the world, with determiners being a less common agreement target than, for example, adjectives; Plank’s^56^ generalization is highly supported (BayesTraits median BF 200, median spatiophylogenetic brms coefficient estimate 2.01 and 95% CI 1.42–2.65). A second example is provided in Supplementary Fig. 11.

**Fig. 3:**
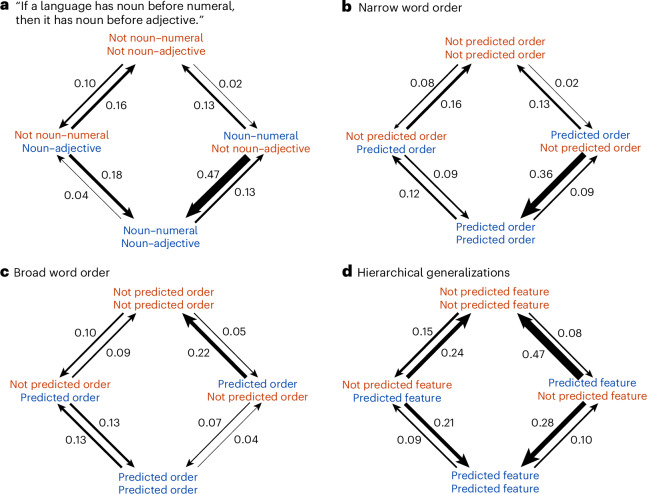
Median rates of change towards harmonic (bottom) and disharmonic states (left and right) for supported universals in BayesTraits analyses (*n* = 60). **a**, An illustration of the dependent model in BayesTraits for the universal ‘if a language has noun before numeral, then it has noun before adjective’^12^. **b**, Median rates of change for supported narrow word order universals. **c***,* Broad word order. **d**, Hierarchical universals. Blue text refers to orders or features predicted by the universal, red text refers to orders or features that the universal does not predict and is agnostic about.

An example of a universal possibly rooted in the interaction between economy and salience (broadly construed as principles on what humans find important) is Croft’s^54^ claim “if there is a construction in which the verb agrees with some member of the relational hierarchy subject ⇒ direct object ⇒ indirect object ⇒ oblique ⇒⇒ [⇒genitive], then there are at least some constructions in which the verb agrees with members higher on that hierarchy”. We tested this proposal by investigating if object indexing implies subject indexing. This is a highly supported claim (BayesTraits median BF 120, median spatiophylogenetic brms coefficient estimate 3.81 and 95% CI of 2.32–5.35), which Croft^54^ explains by stating that verbal indexing is used to mark important or salient arguments, that is, arguments that are high on animacy, definiteness and case scales that line up to be important in the speaker’s perspective on the event. Hierarchical universals, hence, ultimately deal with the expression of those grammatical categories that are most salient for humans across cultures (and more frequent) in comparison with those that are less salient (and less frequent). A complementary account may be a tendency of languages to cover connected regions in conceptual space^10^. They may have diachronic explanations too; see results below (Fig. 3d) on the analyses of rates of change, with a change towards the presence of both features predicted by the universal being more common than the reverse sets of changes away from this state.

### Narrow word order universals

We find support for 37% (24 of 65) of the narrow word order universals (implications between one (or more) word order(s) and another word order). We find support for generalizations between the order of object and verb, the order of adposition and noun, as well as generalizations that describe the order of modifiers and other elements of the noun phrase and the head noun, in relation to other word orders, reflecting findings of previous investigations^12,13,23,38,41,57,58^. An assessment of our results in light of other papers on word order universals is given in Supplementary Text 8; on explanations, see Supplementary Text 9. A key take-away from the latter overview is that the order of adposition and noun is somehow central, as it is involved in the greatest number of supported correlations (Supplementary Fig. 9). Hawkins^12^ has in fact already proposed that “Prep and Postp are more general typological indicators”. There is a tendency for harmonic ordering of the noun and its various adnominal modifiers. Aside from that, VO (verb–object order) languages and OV (object–verb order) languages seemingly evolve differently as they do not engage in the same word order correlations, which is again not a new finding (see, for example, research on the final-over-final condition^59,60^). As is clear from Fig. 2 and Supplementary Fig. 7, some universals receive higher support than others, pointing to a gradience that is in line with the lineage-specificity proposed by Dunn et al.^41^ and findings of other quantitative studies^57,58^. One possible interpretation of our results is that different universals have differential strengths across lineages.

We find evidence for prepositional languages to have a noun–modifier order within the noun phrase and, though less pronounced, for postpositional languages to have modifier–noun order. Such patterns have been explained in terms of strong cognitive predictions associated with ‘headedness’, although the relevant properties differ from author to author^12,13,23,61^. We refer to universals positing a consistent head-dependent order across different phrases or, in other words, a tendency for languages to consistently put the most important words of a phrase in the same position, as ‘harmonic’^62^.

**Fig. 4:**
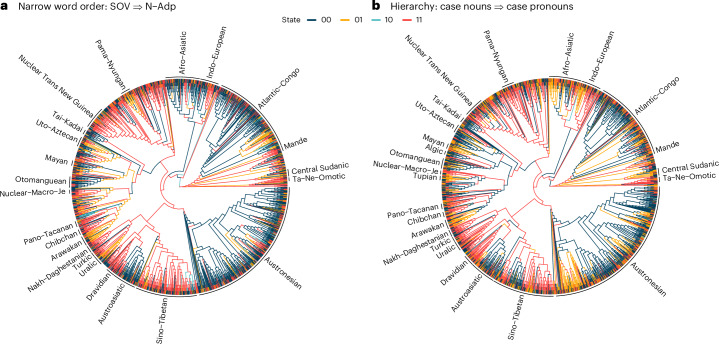
Ancestral state reconstruction of two highly supported universals. State 11 (red) is the prediction made by the universal (the harmonic state 4, see above). State 00 (black) is when both features are absent. State 01 (orange) and 10 (light blue) are disharmonic states (see above). See ‘Ancestral state reconstruction’ section in the Methods for further details. **a**, “With overwhelmingly greater than chance frequency, languages with normal SOV order are postpositional”^38^. **b**, “If there is case-inflection on nouns, there is also case-inflection on some pronouns” (attributed to Edith Moravcsik, source unknown; Supplementary Text 7).

The phylogenetic co-evolution analyses in BayesTraits allow us to assess whether explanations rooted in harmony hold in a diachronic context^10^. We take N–Num ⇒ N–Adj^12^ as an example. The values predicted by the universal, the presence of the orders where nouns precede numerals and adjectives, N–Num and N–Adj respectively, are given state ‘1’, and the reverse order where nouns follow numerals and adjectives, Num–N and Adj–N, are given state ‘0’ (Supplementary Text 5). Such generalizations about states (as shown in Fig. 3a) were coded in the same way: in the BayesTraits models, state 4 (1,1) is harmonic, while state 2 (0,1) and state 3 (1,0) are disharmonic. Note that state 1 (0,0) does not concern us here, as diachronic change to state 4 always goes through state 2 or 3. Figure 3b shows that change to the harmonic state 4 is more frequent than change to disharmonic states for narrow word order universals. This applies to both ‘simple’ word order universals that condition exclusively on a single word order (such as N–Num ⇒ N–Adj), as well as more complex universals that have multiple word order conditions in the implication part (such as Adp–N and N–Num ⇒ N–Adj^12^), where Adp stands for adposition. Figure 4 provides an illustration of this pattern by depicting the support for two highly supported universals on the global phylogeny, showing that the harmonic state is most probable in large chunks of the global tree. We suggest that these findings may benefit future research into the why and how of narrow word order universals, especially considering the interaction between cognitive preference for global head-dependent orders and diachronic explanations rooted in grammaticalization.

### Broad word order and other universals

The broad word order universals are less well supported (11%, 8 of 72) than narrow word order ones, and the rates of change towards combinations of states predicted by the universal are not faster than those towards other state combinations (Fig. 3c). Much the same applies to other universals, only 17% (4 of 24) are supported. Several of the supported broad word order universals are involved in the resolution of argument relationships; supporting the well-studied interaction between grammatical case marking^47,63–66^, the order of adposition and noun, and free word order. Other supported ones are also highly specific: across all four categories of universals, generally formulated universals fare poorly (such as Greenberg’s^38^ universal “if a language is exclusively suffixing, it is postpositional; if it is exclusively prefixing, it is prepositional”). These generally formulated universals make sweeping statements, for example, about all affix positions in an entire language (see above) or about different alignment systems at the same time, as in Nichol’s^47^ “non-accusative alignment may be associated with verb–initial order”. In contrast, more specific universals refer to specific phrase types, parts of speech or function words or morphs. Many broad word order universals, however, are highly specific and nevertheless unsupported; such as Stassen’s^67^ “if attributive adjectives have the form of relative clauses, there is a positive correlation with SVO”.

What unites supported universals of the remaining ‘other’ type is that they tend to relate patterns of morphology, such as Keenan’s^68^ universal “if heads of possessive constructions agree with their possessors in a given language then verbs agree with subjects in that language”. This finding, along with the poor support for broad word order universals in general, may imply that there are few universals that impact both morphology and syntax, except where morphology and syntax ‘connect’, as in argument resolution (Supplementary Text 9).

### Harmonic tendencies across types

**Fig. 5:**
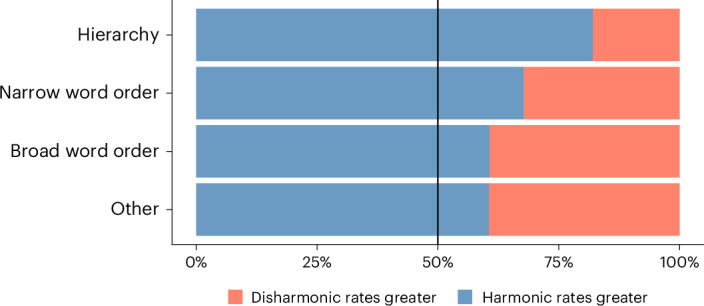
Bar plot showing the proportion of sampled rates from supported universals where the harmonic rate was greater than the disharmonic rate. Distributions in blue show the proportion of times the rates in the harmonic categories were larger than the rates in the disharmonic categories, while distributions in red show the proportion of times the rates in the disharmonic category were larger.

The estimated rates of change for the narrow word order and hierarchy categories (Fig. 3) showed a significant but moderate tendency towards having higher rates towards harmonic states (Fig. 5). We tested significance using two-sided Wilcoxon signed rank tests to avoid making assumptions about normality: narrow word order (*V* = 6,792, *n* = 22 and *P* < 0.001), broad word order (*V* = 1,878, *n* = 10 and *P* = 0.07), hierarchy (*V* = 3,241, *n* = 24 and *P* < 0.001) and other (*V* = 240, *n* = 4 and *P* = 0.21). For narrow word order and hierarchical universals supported in the BayesTraits analyses, the proportion of harmonic rates > disharmonic rates exceeds the expected proportion (0.5). The ‘other’ category was not found to show a significant effect here, presumably owing to its small size (*n* = 4).

## Discussion

In this paper, we overcome the limitations of previous studies of linguistic universals in several ways. First, our large sample of languages from across the world gives us greater statistical power to detect robust linguistic generalizations. It also avoids problems created by small samples focused on a specific region of the world or a small set of language families^42^. Supplementary Fig. 1 shows that the amount of data available in Grambank to test these hypotheses dwarfs that used to propose these hypotheses in the first place. Second, we used appropriate computational methods that explicitly control for the phylogenetic and spatial non-independence of the languages sampled and enabled us to investigate the diachronic relationships between putatively linked grammatical features^57^. In this way, we meet the challenge spelt out by Piantadosi and Gibson^45^ “that claims about linguistic universals should be accompanied by some measure of the strength of evidence in favour of such a universal”. Third, by investigating a large and varied number of putative universals, we can detect general patterns across distinct types of universals with differing rationales and identify common underlying properties. The limitations of this study are that we focused on just the universals listed in the Universals Archive^44^ that could be tested with the data in Grambank (Supplementary Texts 1 and 2). While the statistical models and set of phylogenetic trees we use are the state of the art, it is hoped that future developments will bring further refinements of our results.

We tested 191 putative linguistic universals. The vast majority of these were statistically supported in a naive analysis that did not control for phylogenetic and spatial autocorrelation. However, less than half of these universals were supported once genealogical and geographical relations between the languages were taken into account. In other words, many proposed universals are artifacts of the non-independence of features among closely related or neighbouring languages. This result demonstrates the critical importance of fully controlling for spatial and phylogenetic autocorrelation.

We find statistical support for 60 of the 191 universals. This includes support for most hierarchical universals (80%, 24 of 30) and over a third of the tested narrow word order universals (37%, 24 of 65). The two other types of universals, broad word order universals and ‘other’, do not have the same level of statistical support. Of the 72 broad word order universals we tested, only 8 were found to be supported (11%); of the 24 ‘other’ universals, just 4 were supported (17%). The fact that we find statistical support for a third (31%) of the proposed linguistic universals suggests that, while grammar is far less constrained than linguists have claimed^23,61^, there are indeed some enduring constraints on grammatical variation (contra Dunn et al.^41^). Given that universals differ in strength (Fig. 2 and Supplementary Fig. 7), our results elucidate future directions in universals research, which should be aimed towards explaining gradient support in terms of linguistic, lineage-specific and areal factors. Linguistic theory has generated a multitude of explanatory accounts for universals, and the current study is not in a position to differentiate between their relative merit. Instead, our analyses can shed light on different hypotheses and may be valuable to multiple theoretical perspectives (Supplementary Text 9 and ref. ^69^).

One central take-away is that hierarchical and narrow word order universals are supported more often than other types of universals. Many have argued that the use of harmonic word orders is rooted in processing constraints^13,22,23^. In such an account, the supported universals can then be explained in terms of a cognitive preference for heads and (nominal) dependents to be ordered in the same direction^12,13,23^. Alternatively, in a generative framework, merge and move (internal merge) operations explain such dependencies^70^ or overarching parameter settings^71^. Regardless of the approach, our results support the following long-standing caveat: not all head-dependent pairs engage in correlated behaviour, and some correlations are stronger than others; any account of narrow word orders needs to be able to capture this.

Explanations of hierarchical universals have likewise been rooted in functional and cognitive explanations^9,16,54^, with generative accounts explaining these effects with theoretical mechanisms such as semantic feature geometries^72,73^. We speculate that these are so widely supported because they are highly specific, ingrained in the structure of agreement and other morphological paradigms and possibly rooted in language change^11,74^.

Unlike the narrow word order universals, the broad word order universals do not rely on the concept of harmonic structures in different parts of the grammar. Likewise, the ‘other’ category includes a diverse set of proposed universals. Because both types of universals target such a wide range of morphosyntactic phenomena, the processes that potentially shape them are also more disparate (Supplementary Text 9). We can, however, conclude that there are few links between word order and other aspects of morphosyntax—many of the unsupported broad word order universals deal with supposed characteristics of verb–initial or verb–final languages, as well as languages with free or rigid word order. One of the few exceptions to this is universals involved with the resolution of argument relationships, which are probably involved in a functional trade off^64–66^.

Each individual language could be thought of as an experiment in how to construct an effective communication system that can be learnt and used by both sender and receiver. An enormous array of combinatorial possibilities in all aspects of grammar is available to construct these systems. Dunn et al.^41^ claim that constraints on these possibilities (‘universals’) may arise through lineage-specific processes. Figure 4 depicts the support for two highly supported universals on the global phylogeny. It reveals that, although the feature configurations associated with well-supported universals do tend to be disproportionately concentrated in specific lineages, they also evolve repeatedly in different language families and areas. Thus, despite the flexibility of language pragmatics^75^, and the vast combinatorial array of possible language systems, some aspects of language do repeatedly evolve toward the same preferred regions of the ‘design space’ of morphosyntactic variation. This convergent evolution may reflect common cognitive and communicative pressures, opening the door to integrative accounts of language where language change is a key component, next to functional or formal constraints^26,74^. However, the precise nature of these pressures cannot be identified by large-scale comparative analyses alone. There are numerous possible explanations for these preferred outcomes. Our analyses do not distinguish between different potential causal mechanisms but do provide a restricted set of universals to investigate further from a wide set of theoretical viewpoints. Mechanistic research in the fields of cross-linguistic psycholinguistics, artificial language learning, corpus-based typology, historical linguistics and computer simulation is ideally suited to this task. Combining mechanistic and macro-evolutionary analyses is an exciting challenge for future research that will reveal the complex interplay of factors that shape both linguistic diversity and commonalities.

## Methods

### Universals data

We evaluated over 2,000 documented universals in the Universals Archive^44^ (https://typo.uni-konstanz.de/rara/category/universals-archive/). We compared the morphosyntactic features involved in these universals with the data in Grambank version 1.0^43^ to determine which of them could be analysed using features from Grambank. Then, we reformulated those universals that were testable, first by splitting complex universals that address multiple ostensibly associated linguistic features into simple implicational universals (which we refer to here as simply ‘universals’ or ‘generalizations’). Second, we matched feature constellations from universals to Grambank questionnaire questions and combinations thereof. Since Grambank only covers morphosyntax, any universals involving phonology or semantics were excluded; many other claims were excluded because they were too specific, were explicitly diachronic or were not implicational. From 146 original universals that matched the Grambank variables and our criteria for synchronic implicational claims, we formulated 191 simple universals to test. These were classified into four types (see main text and Supplementary Text 2). For more on the Bayesian statistical analyses including checks for analysis convergence, see below and Supplementary Text 5. All data, code and output that support the findings of this study, including Supplementary Data 1, are available via GitHub (https://github.com/SimonGreenhill/TestingLinguisticUniversals).

### Grammatical data

To obtain grammatical data to test these proposed correlations we used Grambank version 1.0^43^, a database containing morphosyntactic data on 2,430 languages (Supplementary Text 1). Grambank contains information on 195 typological features, which we mapped against the universals from the Universals Archive.

### Spatial data

To control for spatial diffusion, we obtained location data as longitude–latitude pairs from the Glottolog database version 4.3^76^.

### Phylogenetic trees

To control for phylogeny, we used the posterior sample of trees (*n* = 902, downsampled to 100) from a recently released global phylogeny of 6,635 languages^50^.

All matching and creation of datasets was handled in R^49^. For each universal, we used the maximum number of available languages given missing data in Grambank, the Bouckaert et al. phylogenetic trees^50^ or in Glottolog. Ultimately, we sampled between 329 and 2,226 languages, with mean 1,653 and median 1,679 for our analyses (Supplementary Fig. 1 and Supplementary Tables 3 and 4).

### Phylogenetic and spatial correlation

To test whether the proposed universals were indeed correlated after controlling for genealogical and geographical relations, we constructed GLMMs using the package brms^48^ in R^47^. This package allowed us to fit Bayesian multilevel models in R using the probabilistic programming language STAN^77^. We chose brms because it allowed us to include categorical (binary) variables as well as random effects that control for genealogical and spatial relations. Both the response variable (the condition part of the universal) and the main fixed effect (the result part of the universal) are binary. Random effects included phylogenetic distance, spatial distance and macro-area. Phylogenetic and spatial distance were calculated from covariance matrices using trees from ref. ^50^ for the former, and longitude and latitude from Glottolog version 4.3^76^ for the latter. Language areas (‘macro-areas’) were likewise taken from Glottolog version 4.3^76^. Analyses were conducted on 100 phylogenies taken from Bouckaert et al.’s^50^ posterior sample, hence we report on the medians of the main fixed effect (the result part of the universal) and its 95% CI. Our approach is similar to that of Guzmán Naranjo and Becker^78^, who used a correlation matrix in their Bayesian GLMM to deal with genealogical autocorrelation; in contrast, they deal with spatial autocorrelation through Gaussian processes on macro-areas.

### Robustness

To test whether the supported universals using the spatiophylogenetic GLMMs hold without the maximum clade credibility tree from Bouckaert et al.^50^, we constructed another set of GLMMs with brms^48^ in R^49^, which used language family membership as a control instead of the full phylogeny. For these analyses, data on language families was taken from Glottolog version 4.3^76^. Language family membership is a categorical variable construed as a random effect, and both intercepts and slopes were estimated^79^. Only languages from families with five or more members were included.

### Evolutionary dynamics

To model the evolutionary dynamics of the universals that were supported in the spatiophylogenetic GLMMs, we used BayesTraits^51,52^. We mapped our grammatical data onto the Bouckaert et al.^50^ phylogenies using a continuous-time Markov model of trait evolution implemented as the ‘discrete’ model in BayesTraits. We seeded the mean and variance of the gamma prior using a uniform hyperprior between 0 and 10. Correspondingly, each analysis ran for 30,300,000 iterations (the first 300,000 generations were discarded as burn-in), sampling every 30,000 generations. To better explore parameter space among Bouckaert et al.’s^50^ set of posterior phylogenetic trees, we randomly sampled 100 trees (from Bouckaert et al.’s 902 posterior tree sample) and forced BayesTraits to spend an equal amount of time on each of those 100 trees (50,000 iterations). Model fit was assessed using log marginal likelihoods, estimated using stepping stone sampling^80^. Model fit of the dependent and independent model of trait evolution were then compared using natural log BF^81^

$$\begin{array}{l}\log \,\mathrm{Bayes}\,\mathrm{factors}\,=\,\log \,\mathrm{marginal}\,\mathrm{Lh}\,\mathrm{dependent}\,\mathrm{model}\\ \,\,\,\,\,\,\,\,\,\,\,\,\,\,\,\,\,\,\,\,\,\,\,\,\,\,\,{\rm{\mbox{--}}}\,\log \,\mathrm{marginal}\,\mathrm{Lh}\,\mathrm{indep}.\,\mathrm{model}\end{array}$$

A positive Bayes factor indicates that the dependent model performs better than the independent model, providing evidence for co-evolution of both features. Given that we are testing for multiple correlations we use a conservative cut-off. We estimate the 95% HDI of the 100 BFs and take the lower bound of the 95% HDI >10 to constitute significant evidence for co-evolution of two features.

To identify whether these models showed a tendency towards evolving into harmonic states versus disharmonic states, we extracted rate estimates from these analyses. We categorized a subset of the rates in the *Q* matrix as either leading to a harmonic state (both features present) or to a disharmonic state (one feature absent and one present). For example, rate *q*_34_ is the rate at which languages evolve from having feature 1 without feature 2 (1,0) into having both features (1,1) and is therefore harmonic. The opposite rate, *q*_43_, is the rate at which languages evolve from having both features (1,1) to having just the first feature (1,0) and is therefore disharmonic. For each of these pairs of rates, in each generation of the Markov chain Monte Carlo (MCMC) chain, we asked if the harmonic rate was larger than the disharmonic rate, that is, if there was a stronger tendency towards harmonic state patterns.

### Ancestral state reconstruction

To visualize the universals on the phylogeny, we recoded the states of the paired features into a four-state variable (absent + absent = ‘A’, absent + present = ‘B’, present + absent = ‘C’ and present + present = ‘D’). We then fitted a hidden rates model using the corHMM package version 2.8^82,83^ in R^49^ to estimate the marginal maximum likelihood node state probabilities. We modelled the history of these features using an ‘all rates different’ model such that transitions between each state had their own rate. We specified the root probabilities following FitzJohn et al.^84^ and corrected for ascertainment bias following Lewis^85^. Feature manipulation and visualization was done using R packages treeio^86^ and ggtree^87^.

### Reporting summary

Further information on research design is available in the Nature Portfolio Reporting Summary linked to this article.

## Supplementary information

Supplementary Information Supplementary Texts 1–10, References, Figs. S1–S17 and Tables S1–S11.

Reporting Summary

## Data Availability

All the data that support the findings of this study are available via GitHub at https://github.com/SimonGreenhill/TestingLinguisticUniversals.

## Code availability

All code used is available via GitHub at https://github.com/SimonGreenhill/TestingLinguisticUniversals.
