# Supplementary material for: Enduring constraints on grammar revealed by Bayesian spatiophylogenetic analyses
Source: Nat Hum Behav. 2025 Nov 17;10(1):126–36. doi: 10.1038/s41562-025-02325-z (PMC12846912; doi:10.1038/s41562-025-02325-z)
Supplement: Supplementary file 1 — Supplementary Texts 1–10, References, Figs. S1–S17 and Tables S1–S11. [file 41562_2025_2325_MOESM1_ESM.pdf]

nature human behaviour

Supplementary information

https://doi.org/10.1038/s41562-025-02325-z

Enduring constraints on grammar revealed by Bayesian spatiophylogenetic analyses

In the format provided by the authors and unedited

Supplementary Information

Enduring constraints on grammar revealed by Bayesian spatiophylogenetic analyses

Annemarie Verkerk, Olena Shcherbakova, Hannah J. Haynie, Hedvig Skirgård, Christoph Rzymski, Quentin D. Atkinson, Simon J. Greenhill & Russell D. Gray

Nature Human Behavior, Oct. 2025

This file contains the following sections:

Supplementary Information 1
Supplementary Information Text 1: Information on Grambank sample 3
Supplementary Information Text 2: Selection of universals 13
Selection of universals 13
Classification of universals 15
List of universals 16
Supplementary Information Text 3: Implementation of universals using Grambank features 48
Supplementary Information Text 4: Outcome of *brms* and *BayesTraits* analyses 51
Supplementary Information Text 5: Extended statistical methods 62
PGLMMs in *brms*: spatiophylogenetic correlations. 62
GLMMs in *brms*: naïve analyses. 64
GLMMs in *brms*: robustness analyses. 64
BayesTraits: Evolutionary dynamics on a phylogenetic tree. 65
BayesTraits: Analysis of harmonicity. 67
Methodological conceptualization. 68
Supplementary Information Text 6: Comparison of different models 70
Naïve model vs. spatiophylogenetic model. 70
Spatiophylogenetic model vs. language family model 74
Spatiophylogenetic model vs. coevolution in *BayesTraits* 76
Supplementary Information Text 7: Typology of hierarchical universals 78
The relational hierarchy 78

The definiteness hierarchy 79
The nominal hierarchy 79
The agreement hierarchy 80
The feature hierarchy 81
The number hierarchy 81
Supplementary Information Text 8: Assessment of word order universals 83
Supplementary Information Text 9: Explaining support for universals 88
Sample size, bias, and implementation. 88
Narrow word order universals. 91
Hierarchical universals. 100
Broad word order universals. 107
Other universals. 111
Concluding remarks regarding explaining universals. 114
Supplementary Information Text 10: Modeling the results in terms of phylogenetic distance, spatial distance, and macro area 121
Supplementary Information: References 129

All data and code that support the findings of this study, including SI Data 1, are available via GitHub at: https://github.com/SimonGreenhill/TestingLinguisticUniversals

Supplementary Information Text 1: Information on Grambank sample

Grambank is a large-scale database of structural features (Skirgård et al. 2023). Grambank version 1.0 contains 2,430 languages spanning all major families and geographical regions. The Grambank feature set was designed with the aim to cover major domains commonly researched in linguistics such as alignment, pronouns, gender/noun classes, interrogatives, etc. It was also designed with the intention to be possible to fill out for most languages, including those with minimal grammar sketches only. The rest of this section gives insight into the genealogical affiliation of sampled languages, as well as where they are spoken, and lists the relevant part of the Grambank questionnaire.

For the analysis in this paper and the description in this section, we only consider languages that can be linked to the global language phylogeny of Bouckaert et al. (2022), which reduces the number of languages down to 2,226. The Grambank questionnaire consists of 195 features and in the dataset in total there are 24% missing data points. Each of the 191 universals investigated in this study is linked to between 2 and 49 Grambank features. The number of languages without missing data for the relevant set of features for a given universal varies between 329 and 2,226 with a mean at 1,653 (see Figure S1).

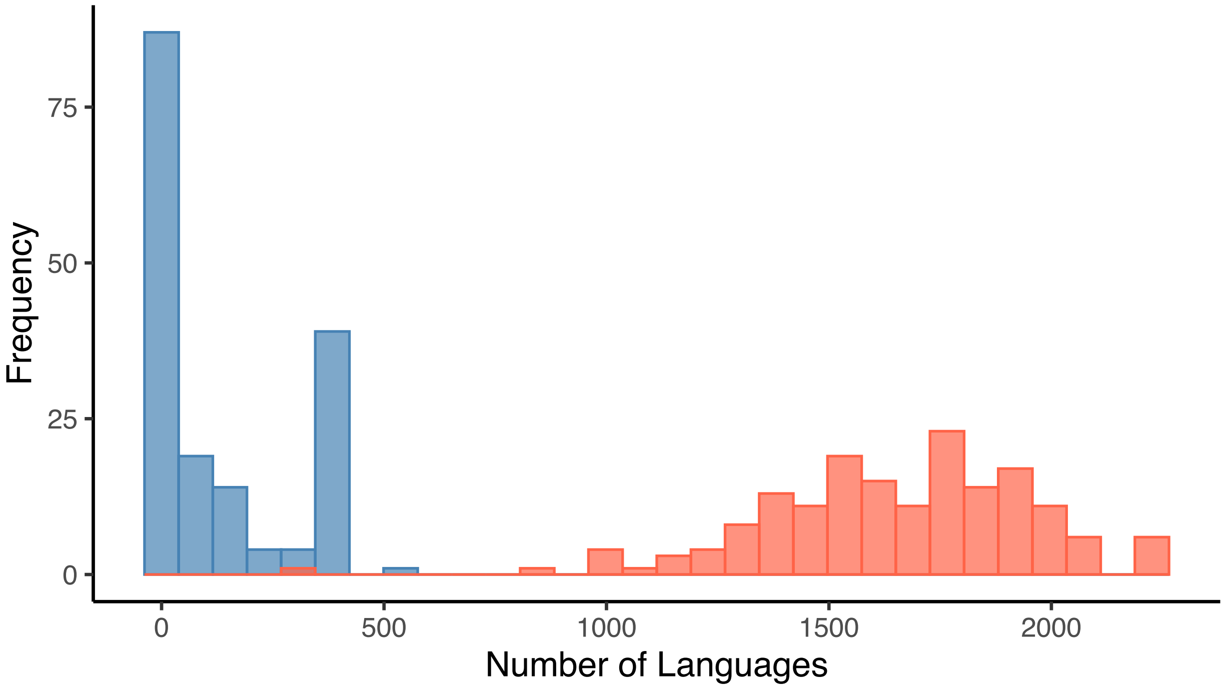

Figure S1. Histogram showing the size of the language samples per universal in Grambank dataset (red), in comparison to the original dataset used to propose the universal in the literature (blue). n = 2 x 168, taking unique combinations of sample size of the source and our sample size.

The map in Figure S2 shows the sample of languages in Grambank version 1.0 which are relevant for this study (i.e. which can be matched to the global phylogeny by Bouckeart et al. 2022). The color of the sampled languages represents the number of universals in whose analyses each language can be included, given missing data. Data coverage is generally good and well spread out over the world.

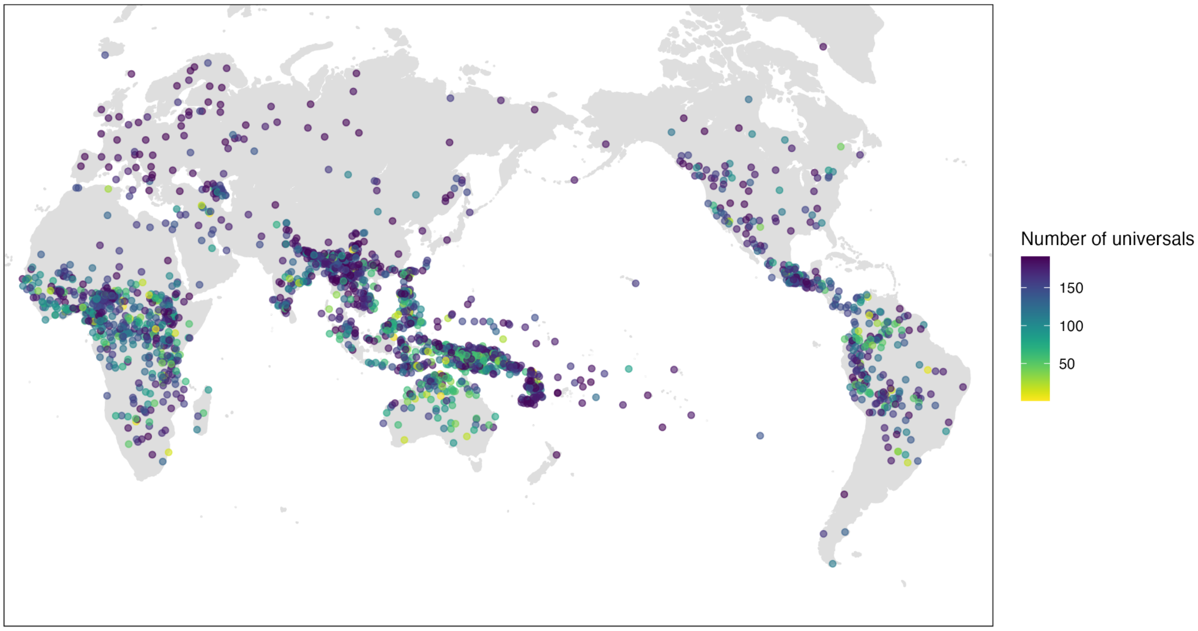

Figure S2. World map visualization of data coverage in the dataset that occurs in both Grambank version 1.0 and the phylogenetic tree sample (Bouckaert et al. 2022); including 2226 languages. The darker the color of the circle representing a language, the more universals have been tested including that language.

As for sample coverage per family and per area, we refer to the Supplementary Information of Skirgård et al. (2023). The overall sampling in Grambank across areas can be assessed in Figure S2 of their paper, which shows the coverage over macro-areas (as defined in Glottolog, Hammarström et al. 2022). The coverage over macro-areas is even, all areas have between 24% and 34% coverage. Languages are marked as "grammar exists" if Glottolog (Hammarström et al. 2022) lists at least one grammar or grammar sketch for the language. Languages without grammars are difficult to include into Grambank, they would require new data collection.

The genealogical coverage of Grambank version 1.0 can be assessed through Figure S3 in Skirgård et al. (2023). Grambank version 1.0 covers between 13% and 68% of the 15 largest

language families as listed on Glottolog (Hammarström et al. 2022). Coverage for Austroasiatic, Isolates (as a group), and Pama-Nyungan is fair, while Indo-European and Tai-Kadai languages are underrepresented.

There are 195 features in the Grambank-questionnaire. 120 of these were involved in at least one putative universal that we tested; these are listed in Table S1.

Table S1: Grambank questionnaire features used in this study

Parameter	Name	Values
GB020	Are there definite or specific articles?	0: absent, 1: present
GB021	Do indefinite nominals commonly have indefinite articles?	0: absent, 1: present
GB022	Are there prenominal articles?	0: absent, 1: present
GB023	Are there postnominal articles?	0: absent, 1: present
GB024	What is the order of numeral and noun in the NP?	1: Num-N, 2: N-Num, 3: both.
GB025	What is the order of adnominal demonstrative and noun?	1: Dem-N, 2: N-Dem, 3: both.
GB030	Is there a gender distinction in independent 3rd person pronouns?	0: absent, 1: present
GB031	Is there a dual or unit augmented form (in addition to plural or augmented) for all person categories in the pronoun system?	0: absent, 1: present
GB041	Are there several nouns (more than three) which are suppletive for number?	0: absent, 1: present
GB042	Is there productive overt morphological singular marking on nouns?	0: absent, 1: present
GB043	Is there productive morphological dual marking on nouns?	0: absent, 1: present
GB044	Is there productive morphological plural marking on nouns?	0: absent, 1: present

GB046	Is there an associative plural marker for nouns?	0: absent, 1: present
GB047	Is there a productive morphological pattern for deriving an action/state noun from a verb?	0: absent, 1: present
GB048	Is there a productive morphological pattern for deriving an agent noun from a verb?	0: absent, 1: present
GB049	Is there a productive morphological pattern for deriving an object noun from a verb?	0: absent, 1: present
GB051	Is there a gender/noun class system where sex is a factor in class assignment?	0: absent, 1: present
GB052	Is there a gender/noun class system where shape is a factor in class assignment?	0: absent, 1: present
GB053	Is there a gender/noun class system where animacy is a factor in class assignment?	0: absent, 1: present
GB054	Is there a gender/noun class system where plant status is a factor in class assignment?	0: absent, 1: present
GB057	Are there numeral classifiers?	0: absent, 1: present
GB059	Is the adnominal possessive construction different for alienable and inalienable nouns?	0: absent, 1: present
GB065	What is the pragmatically unmarked order of adnominal possessor noun and possessed noun?	1: Possessor-Possessed, 2: Possessed-Possessor, 3: both
GB068	Do core adjectives (defined semantically as property concepts such as value, shape, age, dimension) act like verbs in predicative position?	0: absent, 1: present
GB069	Do core adjectives (defined semantically as property concepts; value, shape, age, dimension) used attributively require the same morphological treatment as verbs?	0: absent, 1: present
GB070	Are there morphological cases for non-pronominal core arguments (i.e. S/A/P)?	0: absent, 1: present
GB071	Are there morphological cases for pronominal core arguments (i.e. S/A/P)?	0: absent, 1: present
GB072	Are there morphological cases for oblique non-pronominal NPs (i.e. not S/A/P)?	0: absent, 1: present

GB073	Are there morphological cases for independent oblique personal pronominal arguments (i.e. not S/A/P)?	0: absent, 1: present
GB074	Are there prepositions?	0: absent, 1: present
GB075	Are there postpositions?	0: absent, 1: present
GB079	Do verbs have prefixes/proclitics, other than those that only mark A, S or P (do include portmanteau: A & S + TAM)?	0: absent, 1: present
GB080	Do verbs have suffixes/enclitics, other than those that only mark A, S or P (do include portmanteau: A & S + TAM)?	0: absent, 1: present
GB082	Is there overt morphological marking of present tense on verbs?	0: absent, 1: present
GB083	Is there overt morphological marking on the verb dedicated to past tense?	0: absent, 1: present
GB084	Is there overt morphological marking on the verb dedicated to future tense?	0: absent, 1: present
GB086	Is a morphological distinction between perfective and imperfective aspect available on verbs?	0: absent, 1: present
GB089	Can the S argument be indexed by a suffix/enclitic on the verb in the simple main clause?	0: absent, 1: present
GB090	Can the S argument be indexed by a prefix/proclitic on the verb in the simple main clause?	0: absent, 1: present
GB091	Can the A argument be indexed by a suffix/enclitic on the verb in the simple main clause?	0: absent, 1: present
GB092	Can the A argument be indexed by a prefix/proclitic on the verb in the simple main clause?	0: absent, 1: present
GB093	Can the P argument be indexed by a suffix/enclitic on the verb in the simple main clause?	0: absent, 1: present
GB094	Can the P argument be indexed by a prefix/proclitic on the verb in the simple main clause?	0: absent, 1: present
GB103	Is there a benefactive applicative marker on the verb (including indexing)?	0: absent, 1: present

GB104	Is there an instrumental applicative marker on the verb (including indexing)?	0: absent, 1: present
GB107	Can standard negation be marked by an affix, clitic or modification of the verb?	0: absent, 1: present
GB108	Is there directional or locative morphological marking on verbs?	0: absent, 1: present
GB109	Is there verb suppletion for participant number?	0: absent, 1: present
GB110	Is there verb suppletion for tense or aspect?	0: absent, 1: present
GB111	Are there conjugation classes?	0: absent, 1: present
GB113	Are there verbal affixes or clitics that turn intransitive verbs into transitive ones?	0: absent, 1: present
GB114	Is there a phonologically bound reflexive marker on the verb?	0: absent, 1: present
GB115	Is there a phonologically bound reciprocal marker on the verb?	0: absent, 1: present
GB117	Is there a copula for predicate nominals?	0: absent, 1: present
GB118	Are there serial verb constructions?	0: absent, 1: present
GB119	Can mood be marked by an inflecting word (""auxiliary verb"")?	0: absent, 1: present
GB120	Can aspect be marked by an inflecting word (""auxiliary verb"")?	0: absent, 1: present
GB121	Can tense be marked by an inflecting word (""auxiliary verb"")?	0: absent, 1: present
GB130	What is the pragmatically unmarked order of S and V in intransitive clauses?	1: SV, 2: VS, 3: both
GB131	Is a pragmatically unmarked constituent order verb-initial for transitive clauses?	0: absent, 1: present
GB132	Is a pragmatically unmarked constituent order verb-medial for transitive clauses?	0: absent, 1: present

GB133	Is a pragmatically unmarked constituent order verb-final for transitive clauses?	0: absent, 1: present
GB136	Is the order of core argument (i.e. S/A/P) constituents fixed?	0: absent, 1: present
GB137	Can standard negation be marked clause-finally?	0: absent, 1: present
GB138	Can standard negation be marked clause-initially?	0: absent, 1: present
GB147	Is there a morphological passive marked on the lexical verb?	0: absent, 1: present
GB148	Is there a morphological antipassive marked on the lexical verb?	0: absent, 1: present
GB149	Is there a morphologically marked inverse on verbs?	0: absent, 1: present
GB151	Is there an overt verb marker dedicated to signalling coreference or noncoreference between the subject of one clause and an argument of an adjacent clause (""switch reference"")?	0: absent, 1: present
GB155	Are causatives formed by affixes or clitics on verbs?	0: absent, 1: present
GB165	Is there productive morphological trial marking on nouns?	0: absent, 1: present
GB166	Is there productive morphological paucal marking on nouns?	0: absent, 1: present
GB170	Can an adnominal property word agree with the noun in gender/noun class?	0: absent, 1: present
GB171	Can an adnominal demonstrative agree with the noun in gender/noun class?	0: absent, 1: present
GB172	Can an article agree with the noun in gender/noun class?	0: absent, 1: present
GB184	Can an adnominal property word agree with the noun in number?	0: absent, 1: present
GB185	Can an adnominal demonstrative agree with the noun in number?	0: absent, 1: present
GB186	Can an article agree with the noun in number?	0: absent, 1: present

GB192	Is there a gender system where a noun's phonological properties are a factor in class assignment?	0: absent, 1: present
GB193	What is the order of adnominal property word and noun?	0: they cannot be used attributively, 1: ANM - N, 2: N - ANM, 3: both
GB196	Is there a male/female distinction in 2nd person independent pronouns?	0: absent, 1: present
GB197	Is there a male/female distinction in 1st person independent pronouns?	0: absent, 1: present
GB198	Can an adnominal numeral agree with the noun in gender/noun class?	0: absent, 1: present
GB203	What is the order of the adnominal collective universal quantifier ('all') and the noun?	0: no UQ, 1: UQ-N, 2: N-UQ, 3: both
GB250	Can predicative possession be expressed with a transitive 'habeo' verb?	0: absent, 1: present
GB260	Can polar interrogation be indicated by a special word order?	0: absent, 1: present
GB262	Is there a clause-initial polar interrogative particle?	0: absent, 1: present
GB263	Is there a clause-final polar interrogative particle?	0: absent, 1: present
GB265	Is there a comparative construction that includes a form that elsewhere means 'surpass, exceed'?	0: absent, 1: present
GB266	Is there a comparative construction that employs a marker of the standard which elsewhere has a locational meaning?	0: absent, 1: present
GB273	Is there a comparative construction with a standard marker that elsewhere has neither a locational meaning nor a 'surpass/exceed' meaning?	0: absent, 1: present
GB275	Is there a bound comparative degree marker on the property word in a comparative construction?	0: absent, 1: present
GB276	Is there a non-bound comparative degree marker modifying the property word in a comparative construction?	0: absent, 1: present
GB286	Can polar interrogation be indicated by overt verbal morphology only?	0: absent, 1: present

GB298	Can standard negation be marked by an inflecting word (""auxiliary verb"")?	0: absent, 1: present
GB302	Is there a phonologically free passive marker (""particle"" or ""auxiliary"")?	0: absent, 1: present
GB304	Can the agent be expressed overtly in a passive clause?	0: absent, 1: present
GB309	Are there multiple past or multiple future tenses, distinguishing distance from Time of Reference?	0: absent, 1: present
GB312	Is there overt morphological marking on the verb dedicated to mood?	0: absent, 1: present
GB316	Is singular number regularly marked in the noun phrase by a dedicated phonologically free element?	0: absent, 1: present
GB317	Is dual number regularly marked in the noun phrase by a dedicated phonologically free element?	0: absent, 1: present
GB318	Is plural number regularly marked in the noun phrase by a dedicated phonologically free element?	0: absent, 1: present
GB319	Is trial number regularly marked in the noun phrase by a dedicated phonologically free element?	0: absent, 1: present
GB320	Is paucal number regularly marked in the noun phrase by a dedicated phonologically free element?	0: absent, 1: present
GB321	Is there a large class of nouns whose gender/noun class is not phonologically or semantically predictable?	0: absent, 1: present
GB322	Is there grammatical marking of direct evidence (perceived with the senses)?	0: absent, 1: present
GB323	Is there grammatical marking of indirect evidence (hearsay, inference, etc.)?	0: absent, 1: present
GB327	Can the relative clause follow the noun?	0: absent, 1: present
GB328	Can the relative clause precede the noun?	0: absent, 1: present
GB329	Are there internally-headed relative clauses?	0: absent, 1: present

GB330	Are there correlative relative clauses?	0: absent, 1: present
GB408	Is there any accusative alignment of flagging?	0: absent, 1: present
GB409	Is there any ergative alignment of flagging?	0: absent, 1: present
GB421	Is there a preposed complementizer in complements of verbs of thinking and/or knowing?	0: absent, 1: present
GB422	Is there a postposed complementizer in complements of verbs of thinking and/or knowing?	0: absent, 1: present
GB430	Can adnominal possession be marked by a prefix on the possessor?	0: absent, 1: present
GB431	Can adnominal possession be marked by a prefix on the possessed noun?	0: absent, 1: present
GB432	Can adnominal possession be marked by a suffix on the possessor?	0: absent, 1: present
GB433	Can adnominal possession be marked by a suffix on the possessed noun?	0: absent, 1: present
GB522	Can the S or A argument be omitted from a pragmatically unmarked clause when the referent is inferrable from context (""pro-drop"" or ""null anaphora"")?	0: absent, 1: present

Supplementary Information Text 2: Selection of universals

Selection of universals

Selection of universals from the *Universals Archive* (Plank & Filimonova 2006) has been conducted given the following guidelines.

We have excluded absolute non-implicational universals from our study (e.g. "All languages have passives"), as these can be evaluated by simply studying raw occurrences of values in a typological database – we are interested in dependencies between features of morphosyntax.

We include both absolute implicational universals (those that supposedly have no exceptions) as well as non-absolute implicational universals. It should be noted that universals are interesting even if they have exceptions. If something occurs in nearly all languages, that is still an interesting pattern to explain even if it has not been claimed to occur in all.

We only test implicational universals; no non-implicational ones or ones with an explicit diachronic framing. For example, "Adjectives tend to lose class agreement before number agreement" by Marchese (1988) has a specific diachronic element that we cannot test using the current methods. Greenberg's (1963) first universal "In declarative sentences with nominal subject and object, the dominant order is always one in which the subject precedes the object" is not implicational, so we exclude it.

We include all remaining universals that we can test, even those that have been refuted later on or those we think may not have been made if the authors had access to a bigger or more diverse language sample. To examine whether this inclusive approach is likely to have biased our results, we investigated whether more influential universals, measured by number of citations, show stronger support in our results. In the results file (SI Data 1), we include additional information about each universal, including its original sample, if that original sample was genealogically or areally biased, our assessment on how well it has been implemented given the Grambank questionnaire, how often the source has been cited on Google Scholar, and for some, later studies that tested the universal. Regarding the number of citations, we summed over the different sources given in the *Universals Archive*; different editions of the same source as given on Google Scholar, and we added +1 for every source for which no citations were identified by Google Scholar, given that each source is at least cited once, namely in the *Universals Archive*. We find no evidence for a relationship between the number of citations and the strength of the support for a universal (for the median coefficient estimates from the spatiophylogenetic correlations, Spearman's r is 0.16; for the median BFs from the coevolution analyses, Spearman's r is 0.10).

We do not include universals that have not been listed in the Universals Archive, as we view the Universals Archive as the core collection of linguistic universals that have been proposed. We wish to avoid cherry-picking, i.e. focus on those universals we *a priori* think could be well-supported. This comes with a caveat: none of the sources listed in the Universals Archive is more recent than 2003. The reason for this is probably that it was not maintained actively after 2003, when Frans Plank's project on linguistic universals and rarities ended. We cannot be certain how complete it is, but it covers a great wealth of claims. While the lack of new universals entered for the last two decades may seem to be a major oversight, the lack of updates to the Universals Archive since 2003 also reflects changes in the field. There was, especially in the 1970s and 1980s, a prevalence of publishing statements about cross-linguistic generalizations as strong universals, as described by Dryer (1988), see also Hawkins (1983: 60ff).

> "One can offer an absolute universal as an hypothesis, not because one believes that it is true, but simply because one does not know of any exceptions and by offering it as an hypothesis, one hopes to find an exception if there is one to be found. Offering hypotheses in this way follows the common dictum of formulating hypotheses in the strongest possible form, consistent with the available data. If there are no known exceptions to a generalization, formulating it as an absolute universal hypothesis is making a stronger claim than formulating it as a statistical universal hypothesis. Since formulating an hypothesis as an absolute universal rather than as a statistical one is more likely to result in someone bringing an exception to the attention of other linguists, the absolute form is in general a better way to formulate an hypothesis." Dryer (1998: 21)

We think that increases in the number of languages that are documented, coupled with growing awareness of global linguistic diversity, have led to a decrease in the number of new universals that are proposed. In fact, many more recent studies (Siewierska 1996, Dryer 2002, Sinnemäki 2008, Aldai 2011, de Schepper 2013, Bickel et al. 2015, Culbertson 2017, Schmidtke-Bode 2019, Tang & Her 2019, Berg 2020, Gerdes et al. 2021, Guzmán Naranjo and Becker 2022, Brosa-Rodríguez and Jiménez-López 2023) largely revisit universals first proposed in literature of the 60's and 70's.

We only include bidirectional universals if both directions are explicitly mentioned. We can easily imagine further universals, for example, as part of Dryer's (1992) Branching Direction Theory, but this would radically inflate the number of universals to test. Hence, our study only partially matches the word order universals investigated in Dunn et al. (2011) and Jäger and Wahle (2021).

We only include languages that have data on the condition and the result of each universal.

Many universals included in the *Universals Archive* have a complex structure, such as: "Nominal modifiers (such as relative, adjectival, and attributive expressions) follow nouns in VO languages and precede nouns in OV languages" (Greenberg 1963). We split these up into independent statements so that we can test each of the claims independently.

Additionally, many universals overlap. The *Universals Archive* gives in many ways a historical overview, which captures the same or similar statements that have been made over time. We only test individual unique universals and pick the source (where possible) that is earliest or most salient.

Classification of universals

We classified universals into four domains: narrow word order (65 universals), broad word order (72), hierarchies (30), and 'other' (24). While we can imagine other classifications, we chose these because they invoke well known concepts in the field of linguistics (word order correlations and functional hierarchies) that feature prominently in literature offering explanations for universals (see Supplementary Information Text 9). Word order universals were first introduced by Greenberg (1963), and hierarchies were discovered in the 1970s (Keenan & Comrie 1977, Corbett 1979, Croft 1988). Both have been very influential. What we call 'broad word order' universals, which propose relationships between word order features and other aspects of morphosyntax, have emerged due to the influential nature of word order universals (c.f. Greenberg 1963, Lehmann 1973, Hawkins 1983, Dryer 1992). Having these categories allows us to identify common patterns across a varied range of proposals regarding what is universal in morphosyntax.

Narrow word order universals are those that make reference to word order values in both the condition and the result of the claim, such as "If a language has dominant SOV order and the genitive follows the governing noun, then the adjective likewise follows the noun" (Greenberg 1963, universal no. 5). Narrow word order universals typically invoke the concept of harmonization of word order patterns, especially with regard to the relative order of phrasal heads (e.g. the noun in a noun phrase) and dependent constituents in those phrases (e.g. adjectives in a noun phrase). Broad word order universals relate a word order feature to a feature or combination of features that is not exclusively focused on word order, such as "If in a language the verb follows both the nominal subject and nominal object as the dominant order, the language almost always has a case system" (Greenberg 1963, universal no. 41). Narrow word order universals are discussed further in Supplementary Information Text 8; both narrow and broad word order universals and their explanations feature in Supplementary Information Text 9.

Hierarchies link traits within a single domain or paradigm that have been ranked in frequency or salience on the basis of cross-linguistic research, leading to inferences about patterns of trait co-occurrence (Croft 2003, Haude and Witzlack-Makarevich 2016). They express the expectation

that given the presence of one feature in a language, another related (and typically more general or basic) feature will also be found in that language, such as “If a language has gender categories in the noun, it has gender categories in the pronoun” (Greenberg 1963). Hierarchical universals are discussed further in Supplementary Information Text 7 and 9. Universals in the hierarchy category may also rely on conditional generalizations within the same paradigm, such as “No language has a trial number unless it has a dual. No language has a dual unless it has a plural”, (Greenberg 1963).

The ‘other’ category includes universals that deal with neither word order nor hierarchies. An example is “If the verb has categories of person-number or if it has categories of gender, it always has tense-mood categories” by Greenberg (1963). Many of the universals in this category involve morphology. See for supported ‘other’ universals Supplementary Information Text 9.

List of universals

Table S2 lists all universals that were investigated in this study. The first column, code, refers to the number in the *Universals Archive*; the second column gives the type (bw = broad word order; h = hierarchical; nw = narrow word order; o = other). The third column relates the universal as given in the *Universals Archive*. The fourth column gives the short form, and for those claims that are complex and have to be split up, it also relates which part of the claim is tested. The fifth column gives the source. Several sources are marked with *. These mark sources which we do not cite in the references because the source given in the *Universals Archive* is not correct (the wrongly attributed source can still be accessed in the *Universals Archive*). We have tried our best to retrieve the correct sources, many times also contacting the original author, but failed to retrieve a few. ‘LINFER’ refers to a computer program written in the context of the EUROTYP project in the early nineties. The term probably also refers to unpublished work that Frans Plank had access to while compiling the *Universals Archive*.

Table S2: Universals investigated in this study

code	type	universal	short form	source
0001_or_2aKA	nw	Universal 2. In languages with prepositions, the genitive almost always follows the governing noun, while in languages with postpositions it almost always precedes	Adp-N ⇒ N-Gen	Greenberg (1963)
0001_or_2bKA	nw	same as in previous row	N-Adp ⇒ Gen-N	Greenberg (1963)

0005KA	nw	Universal 5. If a language has dominant SOV order and the genitive follows the governing noun, then the adjective likewise follows the noun.	SOV & N-Gen ⇒ N-Adj	Greenberg (1963)
0008KA	bw	Universal 41. If in a language the verb follows both the nominal subject and nominal object as the dominant order, the language almost always has a case system.	SOV|OSV ⇒ case	Greenberg (1963)
0015aKA	nw	Verbal modifiers like those for negation, causation, and reflexive or reciprocal are placed after verb roots in OV languages and before verb roots in VO languages.	OV ⇒ V-Mod	W. P. Lehmann (1973)
0015bKA	nw	same as in previous row	VO ⇒ Mod-V	W. P. Lehmann (1973)
0045KA	h	If there is a construction in which the verb agrees with some member of the relational hierarchy Subject > DO > IO > Oblique > [Genitive], then there are at least some constructions in which the verb agrees with members higher on that hierarchy .	Obj index ⇒ Sbj index	Croft (1988)
0055KA	nw	Universal 3. Languages with dominant VSO order are always prepositional.	VSO ⇒ Adp-N	Greenberg (1963)

0056KA	nw	Universal 17. With overwhelmingly more than chance frequency, languages with dominant order VSO have the adjective after the noun.	VSO ⇒ N-Adj	Greenberg (1963)
0057aKA	nw	Universal 18. When the descriptive adjective precedes the noun, the demonstrative and the numeral, with overwhelmingly more than chance frequency, do likewise.	Adj-N ⇒ Num-N	Greenberg (1963)
0057bKA	nw	same as in previous row	Adj-N ⇒ Dem-N	Greenberg (1963)
0062aKA	nw	Universal 24. If the relative expression precedes the noun either as the only construction or as an alternate construction, either the language is postpositional, or the adjective precedes the noun or both.	Rel-N ⇒ Adj-N	Greenberg (1963)
0062bKA	nw	same as in previous row	Rel-N ⇒ N-Adp	Greenberg (1963)
0065_or_66KA	nw	If a language has SOV word order, then if the adjective precedes the noun, the genitive precedes the noun. If a language has dominant SOV order and the genitive follows the governing noun, then the adjective likewise follows the noun.	SOV & Adj-N ⇒ Gen-N	Hawkins (1983)
0067_or_68KA	nw	If a language has VSO word order, then if the adjective follows the noun, the genitive follows the noun.	VSO & N-Adj ⇒ N-Gen	Hawkins (1983)

0069KA	nw	If a language has Prep word order, then if the adjective follows the noun, the genitive follows the noun.	Adp-N & N-Adj ⇒ N-Gen	Hawkins (1983)
0070KA	nw	If a language has prepositions and any verb position other than SVO, then if the adjectives follows the noun, the genitive follows the noun.	Adp-N & no SVO & N-Adj ⇒ N-Gen	Hawkins (1983)
0071KA	nw	If a language has Postp word order, and if the adjective precedes the noun, then the genitive precedes the noun.	N-Adp & Adj-N ⇒ Gen-N	Hawkins (1983)
0072KA	nw	If a language has Prep word order, then if the demonstrative determiner follows the noun, the adjective follows the noun.	Adp-N & N-Dem ⇒ N-Adj	Hawkins (1983)
0073KA	nw	If the demonstrative determiner follows the noun, then the adjective follows the noun.	N-Dem ⇒ N-Adj	Hawkins (1983)
0074KA	nw	If a language has Prep word order, then if the numeral follows the noun, the adjective follows the noun.	Adp-N & N-Num ⇒ N-Adj	Hawkins (1983)
0075KA	nw	If a language has noun before numeral, then it has noun before adjective.	N-Num ⇒ N-Adj	Hawkins (1983)
0084KA	h	If in a language the verb agrees with anything, it agrees with some or all subjects.	verb agr ⇒ agr S	Moravcsik (1974)

0087KA	nw	IF a language has Prep word order, then if the demonstrative follows the noun, the relative clause follows the noun.	Adp-N & N-Dem ⇒ N-Rel	Hawkins (1983)
0088KA	nw	If a language has noun before demonstrative, then it has noun before relative clause.	N-Dem ⇒ N-Rel	Hawkins (1983)
0091KA	nw	IF a language has Prep word order, then if the adjective follows the noun, the relative clause follows the noun.	Adp-N & N-Adj ⇒ N-Rel	Hawkins (1983)
0093KA	h	IF a language has pronominal gender in the 2nd person singular, THEN it has a greater probability of having this distinction in the 3rd person singular than of not having it.	gender 2SG ⇒ gender 3SG	Greenberg, Osgood, & Jenkins (1963)
0095aKA	nw	IF there are postpositions, THEN (IF adjectives precede nouns or relatives precede nouns, THEN demonstratives preced nouns and numerals precede nouns) and (IF demonstratives precede nouns or numerals precede nouns, THEN genitives precede nouns).	N-Adp & (Adj-N|Rel-N) ⇒ Dem-N	Hawkins (1983)
0095bKA	nw	same as in previous row	N-Adp & (Adj-N|Rel-N) ⇒ Num-N	Hawkins (1983)

0096KA	nw	IF there are postpositions, THEN (IF adjectives precede nouns or relatives precede nouns, THEN demonstratives preced nouns and numerals precede nouns and possessives preced nouns) and (IF demonstratives precede nouns or numerals precede nouns or possessives precede nouns, THEN genitives precede nouns).	N-Adp & (Adj-N|Rel-N) ⇒ Pron-N	Hawkins (1983)Agr
0101KA	nw	If the possessive adjective follows the noun, then the descriptive adjective follows the noun.	N-Gen ⇒ N-Adj	Hawkins (1983)
0107aKA	nw	Nominal modifiers (such as relative, adjectival, and attributive expressions) follow nouns in VO languages and precede nouns in OV languages	VO ⇒ N-Rel	Greenberg (1963)
0107bKA	nw	same as in previous row	OV ⇒ Adj-N	Greenberg (1963)
0107cKA	nw	same as in previous row	VO ⇒ N-Gen	Greenberg (1963)
0108KA	nw	In consistent OV languages, interrogative markers follow verbs, occupying final position in the sentence.	OV ⇒ Sent-Q	W. P. Lehmann (1973)
0110KA	nw	There is a clear tendency for Rel N languages to be Adj N and for N Rel languages to be N Adj.	N-Rel ⇒ N-Adj	Dryer (1988)

0116KA	h	number is, as has been seen, closer to the stem and generally present when case is present, while the opposite relation holds far more rarely.	case ⇒ number	Greenberg (1963)
0121KA	nw	If a language is prepositional, then if the numeral follows the noun, the relative clause follows the noun.	Adp-N & N-Num ⇒ N-Rel	Hawkins (1983)
0125KA	nw	If a language has both SOV in a full sentence and determining N+determined N in a Noun Phrase, then it has at least some postpositions, which historically come either from verbs (ex. Bengali), or from nouns (ex. Mande languages (W. Africa)).	SOV & Det-N ⇒ N-Adp	Hagège (1982)
0126nKA	nw	If a language has noun before numeral, then it has noun before relative clause.	N-Num ⇒ N-Rel	Hawkins (1983)
0144KA	bw	If a language has no adjective agreement, then the genitive will precede the noun.	no Adj Agr ⇒ Gen-N	LINFER*
0145KA	bw	SOV correlates positively with non-verbal status of predicative adjectives, whereas SVO and VSO have a tendency to favour verbal predicative adjectives.	SVO/VSO ⇒ V-like Pred Adj	Stassen (1992)
0147KA	bw	If attributive adjectives have the form of relative clauses, there is a positive correlation with SVO.	V-like Attr Adj ⇒ SVO	Stassen (1992)

0166KA	h	If a verb agrees in prefix with the direct object, then at least some of the verbs agree with the subject.	pref-V Obj ⇒ V agr Sbj	Kozinsky (1981)
0173KA	nw	If a language is prepositional, then if the demonstrative follows the noun, the genitive follows the noun.	Adp-N & N-Dem ⇒ N-Gen	Hawkins (1983)
0174KA	nw	If a language is prepositional, then if the numeral follows the noun, the genitive follows the noun.	Adp-N & N-Num ⇒ N-Gen	Hawkins (1983)
0175KA	nw	If a language has Prep word order, then if the genitive follows the noun, the relative clause follows the noun.	Adp-N & N-Gen ⇒ N-Rel	Hawkins (1983)
0176KA	nw	If the genitive follows the noun, then the relative clause follows the noun.	N-Gen ⇒ N-Rel	Hawkins (1983)
0177KA	nw	If a language has Postp word order, then if the demonstrative precedes the noun, the genitive precedes the noun.	N-Adp & Dem-N ⇒ Gen-N	Hawkins (1983)
0178KA	nw	If a language has Postp word order, then if the numeral precedes the noun, the genitive precedes the noun.	N-Adp & Num-N ⇒ Gen-N	Hawkins (1983)
0179aKA	h	There is a hierarchy: Number > Gender > Case, such that if some target agrees in one category on the hierarchy then it will also agree in all categories higher on the hierarchy.	gender Adj ⇒ number Adj	LINFER*
0179bKA	h	same as in previous row	gender Dem ⇒ number Dem	LINFER*

0179cKA	h	same as in previous row	gender Art ⇒ number Art	LINFER*
0183KA	h	If there is case-inflection on nouns, there is also case-inflection on some pronouns.	case Nouns ⇒ case PronN	Moravcsik (1993)*
0228KA	o	If nouns inflect for case, then verbs inflect for some inflectional category.	nom case ⇒ verbal infl	Campanella (1638)
0229KA	h	If nominals inflect for number, they also inflect for case.	nom number ⇒ nom case	Campanella (1638)
0230aKA	bw	IF constituent order is rigid, THEN cases are absent, and vice versa; ; If cases are absent, THEN the use of prepositions is extensive, and vice versa;; IF order is rigid, THEN the use of prepositions is extensive, and vice versa. : IF constituent order is flexible, THEN cases are present, and vice versa; ; IF cases are present, THEN the use of prepositions is sparse, and vice versa; ; IF order is flexible, THEN the use of prepositions is sparse, and vice versa.	rigid order ⇒ no case	Girard (1747)
0230bKA	bw	same as in previous row	no case ⇒ Adp-N	Girard (1747)
0230cKA	bw	same as in previous row	rigid order ⇒ Adp-N	Girard (1747)
0230dKA	bw	same as in previous row	free order ⇒ case	Girard (1747)
0230eKA	bw	same as in previous row	case ⇒ no Adp-N	Girard (1747)
0230fKA	bw	same as in previous row	free order ⇒ no Adp-N	Girard (1747)

0231aKA	bw	If constituent order is rigid (or if there is no case inflection), then the gradation of adjectives is expressed by function words; if constituent order is flexible (or if there is case inflection), then the gradation of adjectives is expressed inflectionally.	rigid|no case ⇒ grad adj word	Girard (1747)
0231bKA	bw	same as in previous row	free|case ⇒ gradation adj infl	Girard (1747)
0232KA	bw	If constituent order is rigid and there is no case inflection, then there is a definite article, but not vice versa.	rigid & no case ⇒ def Art	Girard (1747)
0237aKA	h	If adjectives inflect for any category, nouns inflect for this category as well.	number adj ⇒ number noun	Smith (1761) [1983]
0238aKA	bw	If constituent order is rigid, all determined constituents precede their respective determining constituents; in particular, subjects precede their predicates, verbs their complements (objects, prepositional phrases), prepositions their complements (noun phrases), head nouns their attributive adjectives, relative clauses, and complements (prepositional phrases).	rigid order ⇒ SV	Beauzée (1767)
0238bKA	bw	same as in previous row	rigid order ⇒ N-Adj	Beauzée (1767)
0238dKA	bw	same as in previous row	rigid order ⇒ N-Rel	Beauzée (1767)

0239aKA	bw	If constituent order is free, all potentially declinable kinds of nominal words in relationships of determination – nouns, pronouns, adjectives (including articles) – are declined at least for case, the cardinal relational category, but presumably for number and gender as well.	free order ⇒ case noun|Pron	Beauzée (1767)
0239bKA	bw	same as in previous row	free ⇒ num noun adj art	Beauzée (1767)
0239cKA	bw	same as in previous row	free ⇒ gen (pro)noun adj art	Beauzée (1767)
0240aKA	bw	If a language has ergative case marking, it will with far more than chance frequency also have divergent ordering of genitive and adjective attributes (with genitives preceding and with adjectives following their head nouns); if it has divergent genitive and adjective ordering, it will [supposedly with somewhat lower frequency] also have ergative case marking.	ergative case ⇒ Gen-N & N-Adj	Gabelentz (1894)*
0245aKA	bw	If constituent order is free without limits, then nominal words are inflected, but not vice versa; if constituent order is rigid without inversions, then there is no nominal inflection, but again not vice versa.	free order ⇒ infl noun	Beauzée (1767)
0245bKA	bw	same as in previous row	rigid & no inv ⇒ no infl noun	Beauzée (1767)

0247KA	o	The presence of the category of gender is connected with the development of the morphological opposition of nominative/accusative. In those systems where the special form of accusative is attested, the category of gender exists.	accusative ⇒ gender	Palmaitis (1978)
0260KA	o	In ergative languages personal conjugation is of prefixal or prefix-suffixal nature.	ergative ⇒ pref pers conj	Klimov (1973)
0276KA	h	If there is a regular number distinction in the forms of the adjective or of the verb, there is the same distinction in the forms of the noun of the given language.	num (adj|verb) ⇒ num (noun)	Vardul' (1969)
0320KA	h	If personal pronouns have an opposition of the syntactic forms of nominative and accusative, then this opposition is relevant for nouns as well.	nom/acc pron ⇒ nom/acc noun	Kozinsky (1981)
0328KA	h	If a verb agrees with a subject in prefix, then verbs have affixes or clitics which agree with the direct object.	verb agr S prefix ⇒ verb agr P	Kozinsky (1981)
0331aKA	h	If the prefix position is the only possible one for a subject-affix, then the object-affix also occupies this position in all forms (with the exception of Wolio) or at least in some of them (no exceptions).	S pref ⇒ O pref & no O suff (strong)	Kozinsky (1981)

0331bKA	h	same as in previous row	S pref ⇒ O pref | O suff (weak)	Kozinsky (1981)
0332KA	h	If the suffix position is the only possible one for an object-affix, then the subject-affix also occupies this position at least in some cases.	o suffix ⇒ s suffix	Kozinsky (1981)
0344KA	nw	If the order VS is dominant with transitive verbs, then it will be dominant with intransitive verbs as well.	VS (tr. verb) ⇒ VS (intr. verb)	Kozinsky (1981)
0356KA	bw	In the languages where the verb agrees with subject and object, if forms like V-s are the only possible ones, then the dominant order is SV.	verb agr S&P suff ⇒ SV	Kozinsky (1981)
0357KA	bw	If in a dominant word order VS the only possible forms are the forms of the V-s conjugation, then the forms like V-o are also the only possible ones.	VS & suff agr S ⇒ suff agr P	Kozinsky (1981)
0365KA	bw	If the verb agrees with the subject and object and there is at least one o-V form, then the dominant order is SV.	agr S & pref verb P ⇒ SV	Kozinsky (1981)
0386KA	h	If a language has nominal inflection, it also has verbal inflection.	nominal infl ⇒ verbal infl	Moravcsik (1994)
0423KA	nw	If a language has Prep word order, then if the verb position is not SOV, the relative clause follows the noun.	Adp-N & no SOV ⇒ N-Rel	Hawkins (1983)

0439KA	bw	Non-accusative alignment may be associated with verb-initial order.	non-acc align ⇒ V-initial	Nichols (1992)
0446KA	bw	If a language has Verb-final (rigid, or non-rigid) or free word order as (one of) its basic word order(s), then it has postpositions or case affixes.	V-final | free ⇒ N-Adp | case	Rijkhoff (1992)
0454KA	bw	Personal possessive prefixes always imply a GN order, but not the converse; for personal possessive suffixes there is no such rule. Alternative formulation Ultan 1978: 26: "An examination of the 12 languages in the sample having inalienable pronominal prefixes shows that all have a GN constituent order in possessive constructions. In other words, a prefix implies a constituent order of GN in cases of inalienable possession by pronominal affix."	inalien poss pref ⇒ Gen-N	Ultan (1978e)
0489KA	nw	Universal 4. With overwhelmingly greater than chance frequency, languages with normal SOV order are postpositional.	SOV ⇒ N-Adp	Greenberg (1963)

0493aKA	nw	Universal 9. With well more than chance frequency, when question particles or affixes are specified in position by reference to the sentence as a whole, if initial, such elements are found in prepositional languages, and, if final, in postpositional.	Q-Sent ⇒ Adp-N	Greenberg (1963)
0493bKA	nw	same as in previous row	Sent-Q ⇒ N-Adp	Greenberg (1963)
0504aKA	bw	Universal 27. If a language is exclusively suffixing, it is postpositional; if it is exclusively prefixing, it is prepositional.	prefixes ⇒ Adp-N	Greenberg (1963)
0504bKA	bw	same as in previous row	suffixes ⇒ N-Adp	Greenberg (1963)
0506KA	o	If a language has inflection, it always has derivation.	inflection ⇒ word formation	Greenberg (1963)
0507KA	o	If the verb has categories of person-number or if it has categories of gender, it always has tense-mood categories.	(person)-number ⇒ tense-mood	Greenberg (1963)
0511aKA	h	Universal 34. No language has a trial number unless it has a dual. No language has a dual unless it has a plural.	trial ⇒ dual	Greenberg (1963)
0511bKA	h	same as in previous row	dual ⇒ plural	Greenberg (1963)
0513KA	h	Universal 36. If a language has the category of gender, it always has the category of number.	gender ⇒ number	Greenberg (1963)

0516KA	h	Universal 43. If a language has gender categories in the noun, it has gender categories in the pronoun.	gender noun ⇒ gender pron	Greenberg (1963)
0517KA	h	If a language has gender distinctions in the 1st person, it always has gender distinctions in the 2nd or 3rd person, or in both.	gender (1) ⇒ gender (2 &| 3)	Greenberg (1963)
0521KA	h	In more than one language, and possibly in all, if the adnominal adjective agrees with the noun, so does the adnominal demonstrative.	Adj agr noun ⇒ Dem agr noun	Moravcsik (1997)
0564KA	h	If the Dual extends to nouns, it also extends to pronouns.	Du (noun) ⇒ Du (pronoun)	Cuny (1906)
0569KA	o	No (Indo-European) language does without both morphological case marking and articles.	no case (noun) ⇒ article	Renzi (1992)
0572KA	o	If a language has obligatory marking of (in)definiteness, then it has obligatory marking of nominal plurality (but not vice versa).	obli (in)Def mark ⇒ obli PL mark	Gil (1987)
0573KA	o	If a language has obligatory marking of (in)definiteness, then it has no obligatory marking of numeral classification (but not vice versa).	obli (in)Def mark ⇒ no obli NumCl	Gil (1987)
0577KA	h	If any inflectional category is expressed through suppletion in nouns, it is also expressed suppletively in pronouns or verbs.	suppl infl noun ⇒ suppl infl Pron|verb	Moravcsik (1993)*, Sugomoto (1989)*

0582KA	h	If there is any inflection in nouns, there are also some inflections in pronouns.	infl (noun) ⇒ infl (pronoun)	Moravcsik (1993)*, data from Hurford & Kirby (1995)*
0612KA	bw	If there is Switch-Reference marking, languages will mostly be verb-final.	switch-reference ⇒ V-final	Haiman & Munro (1983)
0668KA	nw	Most question particles occur in sentence-initial (or enclitic to the initial constituent) or in sentence-final position. Question particles almost always occur finally in SOV languages and show a greater tendency to occur initially in other types.	SOV ⇒ Sent-Q	Ultan (1978c)
0798KA	o	The Tensedness Universals of Adjective Encoding: A. If a language is tensed, it will have nouny adjectives.	tensed ⇒ N-like Pred Adj	Stassen (1997)
0822KA	bw	Correlatives are limited to verb-final languages, and in fact, are largely limited to "loose" verb-final ones, namely which permit some NPs, especially "heavy" ones to occur to the right of the verb without any special effect of foregrounding or backgrounding.	correlative Rel ⇒ V-final;	Keenan (1985)
0837KA	bw	VSO languages tend to have Person-Number inflection. Standardized: IF basic order is VSO, THEN there is very likely to be verbal person-number inflection or both person-number and tense-aspect-mood inflection, but not verbal tense-aspect-mood	VSO ⇒ person & number infl	Bybee (1985)

		without person-number inflection.		
0889aKA	bw	OV languages tend to have suffixes and VO languages prefixes.	OV ⇒ suffixes	W.P.Lehmann (1978)
0889bKA	bw	same as in previous row	VO ⇒ prefixes	W.P.Lehmann (1978)
0917aKA	nw	Consistent OV languages are consistently left-branching (placing phrasal constituents before nonphrasal ones) while consistent VO languages are consistently right-branching.	VO ⇒ Qpart-S	Dryer (1988a)
0917bKA	nw	same as in previous row	OV ⇒ Gen-N	Dryer (1988a)
0917cKA	nw	same as in previous row	OV ⇒ Rel-N	Dryer (1988a)
0964aKA	nw	VO languages exhibit a statistically greater tendency to place articles before the noun than OV languages do. To the opposite, the tendency to place the noun before the articles is higher among OV languages than it is among VO languages.	VO ⇒ Art-N	Dryer (1989a)
0964bKA	nw	same as in previous row	OV ⇒ N-Art	Dryer (1989a)

0982KA	bw	If a language has a locative comparative, then it is either SOV or VSO.	loc compa ⇒ SOV|VSO	Stassen (1984)
0983KA	bw	If a language has an exceed comparative, then it is SVO.	exceed compa ⇒ SVO	Stassen (1984)
1027aKA	bw	There are correlations between following parameters: VO word order, Non-tensedness, Non-casedness, WITH-Construction, Non-Absolute Converb. Standardized IF VO, THEN Non-tensedness, Non-casedness, WITH-Construction, and Non-Absolute Converb. IF Non-tensedness, THEN VO, Non-casedness, WITH-Construction, and Non-Absolute Converb. IF Non-casedness, THEN VO, Non-tensedness, WITH-Construction, and Non-Absolute Converb. IF AND-construction, THEN VO, Non-tensedness, Non-casedness, and Non-Absolute Converb. IF Non-Absolute Converb, THEN VO, Non-tensedness, Non-casedness, and WITH-Construction.	VO ⇒ nomorph tense | no case	Stassen (1997)
1027bKA	bw	same as in previous row	no morph tense ⇒ no case | VO	Stassen (1997)
1027cKA	bw	same as in previous row	no case ⇒ no morph tense | VO	Stassen (1997)

1028aKA	bw	There are correlations between following parameters: OV word order, Tensedness, Casedness, AND-Construction, Absolute Converb. IF basic word order OV, THEN Tensedness, Casedness, AND-Construction, and Absolute Converb. IF Tensedness, THEN basic word order OV, Casedness, AND-Construction, and Absolute Converb. IF Casedness, THEN basic word order OV, Tensedness, AND-Construction, and Absolute Converb. IF AND-construction, THEN basic word order OV, Tensedness, Casedness, and Absolute Converb. IF Absolute Converb, THEN basic word order OV, Tensedness, Casedness, and AND-Construction.	OV ⇒ morph tense | case	Stassen (1997)
1028bKA	bw	same as in previous row	morph tense ⇒ case | OV	Stassen (1997)
1028cKA	bw	same as in previous row	case ⇒ morph tense | OV	Stassen (1997)
1053KA	h	If determiners agree within NPs, modifiers are likelier also to agree than not to agree.	agr Dets ⇒ agr Mods	Plank (1994)
1111KA	bw	Lack of inflectional morphology implies fixed word order of direct nominal arguments. The converse is	no infl morph ⇒ fixed order args	Kiparsky (1997)

		not true, hardly even a tendency.		
1114KA	nw	VO languages are exceptionlessly Comp-initial. OV languages exemplify both final complementizers and initial complementizers.	VO ⇒ Comp-S	Hawkins (1990)
1142aKA	bw	Passivization is prominent in SVO languages, but not at all in OV languages.	passive ⇒ SVO	W.P.Lehmann (1978)
1142bKA	bw	same as in previous row	no passive ⇒ SOV	W.P.Lehmann (1978)
1152KA	nw	For any language, preposed relative clause implies postposed relative clause.	Rel-N ⇒ N-Rel	Moravcsik (1969)
1163KA	h	If a language has a grammaticalized indefinite article, it is likely to also have a definite article, while the reverse does not necessarily hold true.	indefinite Art ⇒ definite Art	Moravcsik (1969)
1216KA	bw	If there are (local) postpositions, then there will also be nominal case inflection (not necessarily including local cases, though).	N-Adp ⇒ case (noun)	Stolz (1992)
1267aKA	bw	Ergative systems are found only in SOV and VSO languages. SVO languages are never ergative.	ergative ⇒ VSO | SOV	Schwartz (1972) & Trask (1979)

1267bKA	bw	same as in previous row	SVO ⇒ not ergative	Schwartz (1972) & Trask (1979)
1334aKA	nw	Prepositional Noun-Modifier Hierarchy (PrNMH): If a language is prepositional, then if Relative clause Noun order then Genitive Noun order, if Genitive Noun order then Adjective Noun order, and if Adjective Noun order then Demonstrative Noun order.	Adp-N & Rel-N ⇒ Gen-N	Hawkins (1983)
1334bKA	nw	same as in previous row	Adp-N & Rel-N & Gen-N ⇒ Adj-N	Hawkins (1983)
1334cKA	nw	Prepositional Noun-Modifier Hierarchy (PrNMH): If a language is prepositional, then if Relative clause Noun order then Genitive Noun order, if Genitive Noun order then Adjective Noun order, and if Adjective Noun order then Demonstrative Noun order.	Ap-N & RC-N & Ge-N & Aj-N ⇒ Dm-N	Hawkins (1983)
1341KA	o	If a language has a verbal reciprocal/reflexive, it must have an overt AGR marker. Standardized IF there is a verbal reciprocal/reflexive, THEN there must be verb agreement at least with subject.	verb reci/refl ⇒ verb agr S	Subbarao (1997), Subbarao (1998)
1349KA	o	Numeral classifiers occur in the languages where plural is weakly developed.	weak PL ⇒ Num Cl	Serebrennikov (1974)

1372KA	bw	A lexically distinct form of verb HAVE is generally missing in verb peripheral languages (i.e. SOV, VOS). That is, a verb HAVE is generally confined to SVO languages.	verb HAVE ⇒ SVO	Mahajan (1997)
1415KA	o	If heads of possessive constructions agree with their possessors in a given language then verbs agree with subjects in that language. Standardized IF heads of possessive constructions (=possessees) agree with their possessors, THEN verbs agree with subjects.	poss agr ⇒ verb agr S	Keenan (1974)
1427KA	o	If in a language there are no prefixes, then there will be inflectional (i.e. not prepositional) local cases.	no prefix ⇒ inflectional local cases	Serebrennikov (1974)
1430KA	o	If a language has possessive suffixes on nouns, most commonly it will lack overt copula form (of 'to be') in the present tense.	N-poss suff ⇒ no PRS Cop	Serebrennikov (1974)
1431mKA	o	The more developed a case system is, the less is its system of verbal tenses.	less case ⇒ more tense	Serebrennikov (1974)
1431nKA	o	same as in previous row	more case ⇒ less tense	Serebrennikov (1974)
1434KA	o	A large number of tense oppositions correlates with the absence of aspect differentiation.	many tenses ⇒ no aspect	Serebrennikov (1974)

1436KA	bw	If in a language the adjective follows the noun, there is an overt copula form (of 'to be') in the present tense form.	N-Adj ⇒ PRS overt cop	Serebrennikov (1974)
1499KA	o	Languages with serial verb constructions tend to have VO ordering with verb serialization and one object per verb (but some permit two objects); they tend to be isolating, with little inflectional morphology. Syllable structure is typically simple, and many have lexical and/or grammatical tone distinctions.	SVC ⇒ little infl	Lord (1993)
1531KA	bw	Verb-initial languages evidence significant prefixing, though normally there is some suffixing as well. There is a possibility of ambi-fixing (discontinuous affixes), and a somewhat greater than chance tendency for discontinuous demonstratives.	V-initial ⇒ prefixes	unpublished statements of Keenan's, reproduced in Payne (1990)
1534KA	nw	In verb-initial languages, the question particle, if any, occurs sentence initial in yes-no questions.	V-initial ⇒ Q-Sent	unpublished statements of Keenan's, reproduced in Payne (1990)
1537KA	nw	In verb-initial languages, subordinate markers such as complementizers, nominalizers, and subordinate conjunctions precede their clauses.	V-initial ⇒ C-Sent	unpublished statements of Keenan's, reproduced in Payne (1990)

1544KA	bw	In verb-initial languages, verbal case marking is attested to a very significant degree. IF word order is verb-initial, THEN there is relational marking (agreement, applicative, etc.) associated with the verb. Standardized IF word order is verb-initial, THEN there is relational marking (agreement, applicative, etc.) associated with the verb.	V-initial ⇒ verbal case	unpublished statements of Keenan's, reproduced in Payne (1990)
1545KA	nw	In verb-initial languages, the demonstrative, numeral, and qualifying adjective follow the common noun in that order or its mirror image (Adj + Num + Dem).	V-initial ⇒ N-Dem-Num-Adj	unpublished statements of Keenan's, reproduced in Payne (1990)
1546aKA	bw	In verb-initial languages, there is probably less agreement of adjectives with common nouns than in verb-final languages, especially case agreement.	V-final ⇒ more Adj-N agr	unpublished statements of Keenan's, reproduced in Payne (1990)
1546bKA	bw	same as in previous row	V-initial ⇒ less Adj-N agr	unpublished statements of Keenan's, reproduced in Payne (1990)
1548aKA	bw	In verb-initial languages, the existence of several articles (definite, indefinite, specific, plural, proper noun) is much more common than in verb-final languages.	V-final ⇒ no several Art	unpublished statements of Keenan's, reproduced in Payne (1990)

1548bKA	bw	same as in previous row	V-initial ⇒ several Art	unpublished statements of Keenan's, reproduced in Payne (1990)
1549KA	nw	With great regularity possessor NPs follow the head NP in verb-initial languages.	V-initial ⇒ N-Gen	unpublished statements of Keenan's, reproduced in Payne (1990)
1550KA	nw	In verb-initial languages the dominant order of relative clauses is always postnominal.	V-initial ⇒ N-Rel	unpublished statements of Keenan's, reproduced in Payne (1990)
1559KA	bw	Verb-initial languages always have a passive voice and it is almost always marked in the verbal morphology (rather than by a serial verb construction as in Chinese, for example).	V-initial ⇒ passive voice	unpublished statements of Keenan's, reproduced in Payne (1990)
1560KA	bw	With possibly greater than chance frequency, the verb in verb-initial languages either agrees with no NPs, or with two NPs (both subject and direct object, or sometimes subject and indirect object).	V-ini ⇒ no verb agr | agr 2 NPs	unpublished statements of Keenan's, reproduced in Payne (1990)
1561KA	bw	Verb-initial languages normally have no overt copula.	V-initial ⇒ no overt copula	unpublished statements of Keenan's, reproduced in Payne (1990)

1586KA	o	An opposition of "alienable" and "inalienable" possession is almost inevitable in languages where possession is regularly head-marked. The alienable-inalienable opposition is almost never signaled by dependent-marked morphology.	Poss head-m ⇒ alien-ina opp	Nichols (1992)
1589KA	bw	Verb-final languages exhibit a very strong postposing tendency which leads to their preponderance of suffixes.	V-final ⇒ postp & suff	Bybee Pagliuca & Perkins (1990)
1591KA	bw	Clear cases of internal relative clauses are present only in languages whose basic word order is SOV.	int Rel ⇒ SOV	Keenan (1985b)
1592KA	bw	The restriction of internally headed relative clauses to OV languages implies that these relative clauses are found in languages having left-branching rather than (in addition to) right-branching NP structure.	int Rel ⇒ left-branching NP	Cole (1987)
1593KA	o	Internally headed relative clauses occur only in languages manifesting null anaphora (that is, the use of null NPs in place of lexical pronouns etc. in most argument positions).	int Rel ⇒ null anaphora	Cole (1987)

1610KA	bw	In languages with verb agreement, patient verb agreement appears more frequently in verb-peripheral languages [i.e. verb-initial and verb-final languages] than in verb-medial languages.	patient agr ⇒ V-initial|V-final	Foster & Hofling (1987)
1611KA	h	All languages with verb-patient agreement, regardless of type, also have verb agreement with the agent as well.	P agr verb ⇒ A agr verb	Foster & Hofling (1987)
1612aKA	bw	In SOV languages, when the expected order of Modifier-Noun does not occur, agreement [between noun and modifier] tends to appear.	SOV & N-Art ⇒ N-Art agr	Foster & Hofling (1987)
1612bKA	bw	same as in previous row	SOV & N-Adj ⇒ N-Adj agr	Foster & Hofling (1987)
1612cKA	bw	same as in previous row	SOV & N-Num ⇒ N-Num agr	Foster & Hofling (1987)
1612dKA	bw	same as in previous row	SOV & N-Dem ⇒ N-Dem agr	Foster & Hofling (1987)
1621KA	o	If a language has adjectives, then the numeral tends to modify the noun directly (that is, in most languages that have adjectives the numeral does not require the occurrence of a sortal classifier), but not vice versa.	Adj ⇒ no sortal Cl	Rijkhoff (2000)
1653KA	o	If a language is non-tensed, the nominal predicate does not require a copula.	non-tensed ⇒ no cop nom Pred	Kalinina (1998)

1667aKA	nw	There is a tendency for conjunctions (including comlementizers) to occur in clause-final position in SOV languages and in clause-initial position in VSO languages.	S-Comp ⇒ SOV	Kuno (1974)
1667bKA	nw	same as in previous row	Comp-S ⇒ VSO	Kuno (1974)
1741KA	o	There is more prefixing on verb than on noun. If a language has any prefixes on noun, it will also have prefixes on verb with considerably more than chance frequency.	Gen pref ⇒ verbal prefs	Cutler, Hawkins, & Gilligan (1985); Hawkins & Gilligan (1988), C. Lehmann & Moravcsik (2000)
1827KA	nw	If a language shows the order RelNoun or AdjNoun in its noun phrase, it is very likely to also show PossNoun, but not vice versa.	Rel-N | Adj-N ⇒ Gen-N	Givón (1971)

1994aKA	bw	Hier nun war es möglich, scheinbar sich sogar auf einen einzigen Gegenstand zu beschränken, nämlich DIE STELLUNG DES AFFIXLOSEN GENITIVS in bezug auf den Nominativ, den er näher bestimmt. Scheinbar, denn in Wirklichkeit schließen an diesen Gegenstand in psychologischer Notwendigkeit eine ganze Reihe von grundlegenden Elementen der Satzbildung sich an und werden durch ihn in weitgehendem Maße bestimmt und beeinflußt. Es steht mit der Genitivstellung in engstem Zusammenhang die Bildung und Stellung des vom Personalpronomen abgeleiteten POSSESSIVS. Bei dessen tieferer Behandlung stößt man auch auf das Problem der Form und Stellung des PERSONALPRONOMENS ALS SUBJEKT DER SÄTZE, insbesondere der Verbalsätze. Nachdem in diesen beiden wichtigen Punkten die weitgehende Bedeutung der Genitivstellung zutage getreten ist, wirft sich bald auch die Frage auf, ob nicht auch bei einem anderen wichtigen Kasus, dem AKKUSATIV, seine Stellung	non-affix Gen-N ⇒ N-Adp	Schmidt (1926)

		zu dem Verb, von dem er abhängt, in enger Beziehung zu der des Genitiv stehe oder gar von ihr maßgebend bestimmt werde. Die gleiche Frage drängt sich dann auch auf in bezug auf die Stellung des ADJEKTIVS zu dem Substantiv, das es näher bestimmt. Standardized IF the attributive noun (genitive, esp. when morphologically unmarked) precedes the head noun, THEN (i) nominal affixes (for case, number, gender/class) and adpositions are postposed (suffixes, postpositions), (ii) pronominal possessives precede the head noun, (iii) attributive adjectives precede the head noun, (iv) verbal affixes (person, number) and pronouns (for subject) precede the verb, (v) direct objects (accusative, esp. when morphologically unmarked) precede the verb.		

1994bKA	bw	same as in previous row	non-affixed Gen-N ⇒ OV	Schmidt (1926)
1994cKA	bw	same as in previous row	non-aff Gen-N ⇒ verb pref pers num	Schmidt (1926)
1994dKA	bw	same as in previous row	non-aff Gen-N ⇒ Adj-N	Schmidt (1926)
2012KA	o	A system of switch-reference marking is found only in languages with an accusative syntax.	switch-reference ⇒ accusitive	Aikhenvald & Dixon (1998)

Supplementary Information Text 3: Implementation of universals using Grambank features

All implementations and assessments of implementations are provided in the Supplementary Information Data 1 file, results.txt. Implementation issues that apply across several universals are summarized here.

Word order universals. There are several issues that we encounter in implementing word order universals from the *Universals Archive* using Grambank features. First of all, many word order universals revolve around the order of subject, verb, and object, or around the order of verb and object. We can only approximate this using Grambank features, because the feature questionnaire does not explicitly track the position of all three arguments. Instead, there are the following binary features which cover the position of the verb:

1. GB131 Is a pragmatically unmarked constituent order verb-initial for transitive clauses?
2. GB132 Is a pragmatically unmarked constituent order verb-medial for transitive clauses?
3. GB133 Is a pragmatically unmarked constituent order verb-final for transitive clauses?

Hence, we implement common word order patterns using Grambank features as follows:

Verb-initial / VSO:	GB131:1 & GB132:0 & GB133:0
Verb-final / SOV:	GB131:0 & GB132:0 & GB133:1
Verb-medial / SVO:	GB131:0 & GB132:1 & GB133:0
OV:	GB131:0 & (GB132:1 | GB133:1)
VO:	(GB131:1 | GB132:1) & GB133:0

Regarding the order of S, V, and O; this implementation means that VSO languages are grouped with VOS languages; and SVO languages are grouped with OVS languages; both VOS and OVS order are exceedingly rare and this choice should not affect our results. Regarding the order of V and O, we took a more lenient implementation in order to include VSO and SVO languages in a single type for VO. The implementation of OV is the mirror image of the VO implementation.

Secondly, many word order universals deal with 'basic' or 'dominant' word order (Greenberg 1963: Sect 2. 'The Basic Order Typology'). Both Hawkins (1983: 13) and Dryer (2013) make reference to frequency:

"Where one doublet (e.g., NAdj) occurs with greater frequency than the other (AdjN) in attested samples of the relevant language, then, all things being equal, the more frequent doublet is the basic one." Hawkins (1983: 13)

"The rule of thumb employed is that if text counts reveal one order of a pair of elements to be more than twice as common as the other order, then that order is considered dominant, while if the frequency of the two orders is such that the more frequent order is less than twice as common as the other, the language is treated as lacking a dominant order for that pair of elements. For sets of three elements, one order is considered dominant if text counts reveal it to be more than twice as common as the next most frequent order; if no order has this property, then the language is treated as lacking a dominant order for that set of elements." Dryer (2013)

Since the Grambank classification does not base itself on such a frequency-based classification, our operationalization cannot capture this aspect of these generalizations. However, the relevant Grambank features clearly specify "pragmatically unmarked constituent order", and while this is not the same as 'dominant' or 'basic', it could be said that this means the relevant word order is at least not rare or marked.

Thirdly, while most Grambank features are binary, a handful are not, they may take three values. These are:

GB024 on numeral-noun order:	1 = Num-N, 2 = N-Num, 3 = both
GB025 on demonstrative-noun order:	1 = Dem-N, 2 = N-Dem, 3 = both
GB065 on genitive-noun order:	1 = Possessor-Possessed,<br>2 = Possessed-Possessor, 3 = both
GB193 on adjective-noun order:	1 = ANM-N, 2 = N-ANM, 3 = both
GB024 on universal quantifier-noun order:	1 = UQ-N, 2 = N-UQ, 3 = both
GB130 on subject verb order:	1 = SV, 2 = VS, 3 = both

When these Grambank features are used, we have chosen to not include value 3, 'both', anywhere, which we believe is a conservative approach. This has the downside that we lose some languages in which the relevant order is actually attested; but the advantage is that we do not include languages which also display the opposite word order.

Other types of universals. Some grammatical categories appear across a wide range of universals. Hence, we have unified our implementation of these categories wherever this was possible:

Case: sum(GB070:1,GB071:1,GB072:1,GB073:1) => 1
No case: GB070:0
Where:

1. GB070 Are there morphological cases for non-pronominal core arguments (i.e. S/A/P)?
2. GB071 Are there morphological cases for pronominal core arguments (i.e. S/A/P)?
3. GB072 Are there morphological cases for oblique non-pronominal NPs (i.e. not S/A/P)?

4. GB073 Are there morphological cases for independent oblique personal pronominal arguments (i.e. not S/A/P)?

Gender:
sum(GB030:1,GB051:1,GB052:1,GB053:1,GB054:1,GB192:1,GB321:1,GB198:1) > 0
Where for gender:

1. GB030 Is there a gender distinction in independent 3rd person pronouns?
2. GB051 Is there a gender/noun class system where sex is a factor in class assignment?
3. GB052 Is there a gender/noun class system where shape is a factor in class assignment?
4. GB053 Is there a gender/noun class system where animacy is a factor in class assignment?
5. GB054 Is there a gender/noun class system where plant status is a factor in class assignment?
6. GB192 Is there a gender system where a noun's phonological properties are a factor in class assignment?
7. GB321 Is there a large class of nouns whose gender/noun class is not phonologically or semantically predictable?
8. GB198 Can an adnominal numeral agree with the noun in gender/noun class?

Number:
sum(GB041:1,GB042:1,GB043:1,GB044:1,GB165:1,GB166:1,GB316:1,GB317:1,GB318:1,GB319:1,GB320:1) => 1
Where for number:

1. GB041 Are there several nouns (more than three) which are suppletive for number?
2. GB042 Is there productive overt morphological singular marking on nouns?
3. GB043 Is there productive morphological dual marking on nouns?
4. GB044 Is there productive morphological plural marking on nouns?
5. GB165 Is there productive morphological trial marking on nouns?
6. GB166 Is there productive morphological paucal marking on nouns?
7. GB316 Is singular number regularly marked in the noun phrase by a dedicated phonologically free element?
8. GB317 Is dual number regularly marked in the noun phrase by a dedicated phonologically free element?
9. GB318 Is plural number regularly marked in the noun phrase by a dedicated phonologically free element?
10. GB319 Is trial number regularly marked in the noun phrase by a dedicated phonologically free element?
11. GB320 Is paucal number regularly marked in the noun phrase by a dedicated phonologically free element?

Supplementary Information Text 4: Outcome of *brms* and *BayesTraits* analyses

The main text discusses results from four analyses:

1. *brms* analyes without controlling for genealogical and geographical relations, depicted by Figure 1 and S4;
2. *brms* analyses controlling for genealogical and geographical relations. These are conducted over 100 phylogenetic trees taken from Bouckaert et al. (2022) (see Methods), hence we take medians for the estimate and its 95% Credible Interval. They are depicted in Figure 1 and S4;
3. *brms* analyses using family as a categorical control, reported in the section of the Results entitled "Robustness" and depicted in Figure S6;
4. *BayesTraits* analyses controlling for genealogical relations, reported in the section of the Results entitled "Evolutionary Dynamics" and depicted in Figure 2 and S6. In the main text and the Table below we present Natural log Bayes Factors (BF) and their 95% High Density Intervals (95% HDI). Universals with the lower bound of the 95% HDI on the distribution of BF > 10 are considered supported.

In this section, tables of the full results are presented (Table S3 and S4), including sample sizes, Bayes' Factors and estimates of the relevant effects, their CIs and HDIs, and Boolean values indicating whether the universal is supported yes/no. Sample size (the number of languages included in the analysis) is identical for analyses 1, 2, and 4, and indicated with the column 'm_n' in both tables. The sample size for analysis 3 is smaller and listed in column 'f_n' in Table S3. The same is presented in a machine-readable version in SI Data 1. SI Data 1 also includes the additional *brms* analyses where distances between languages were calculated differently (see SI Section 5, subsection 'PGLMMs in *brms*: spatiophylogenetic correlations'). These control for geographical and genealogical relations; the latter using MCC tree from Bouckaert et al. (2022). The relevant columns in SI Data 1 are 'COVAR_Estimate'; 'COVAR_low_95_CI'; 'COVAR_upp_95_CI'; and 'COVAR_SIG'; these do not feature in Table S3 and S4.

Table S3: Results from the *brms* analyes without controlling for genealogical and geographical relations (columns 3-6; sample size (*n* languages) in column 2) and the *brms* analyses using family as a categorical control (columns 8-11; sample size in column 7).

code	m_n	UNCON_Estimate	UNCON_low_95_CI	UNCON_upp_95_CI	UNCON_SIG	f_n	FAM_Estimate	FAM_low_95_CI	FAM_upp_95_CI	FAM_SIG
0001_or_2aKA	1958	2.68	2.43	2.94	SIG	1659	3.22	1.47	4.94	SIG
0001_or_2bKA	1881	2.31	2.10	2.52	SIG	1591	1.39	-0.31	2.82	NOT SIG
0005KA	1787	-0.74	-0.94	-0.53	SIG	1512	-0.29	-1.99	1.48	NOT SIG
0008KA	1772	1.26	1.04	1.49	SIG	1484	0.96	-0.52	2.57	NOT SIG
0015aKA	1941	1.83	1.51	2.17	SIG	1635	1.96	0.34	3.52	SIG
0015bKA	1926	0.92	0.72	1.12	SIG	1620	1.13	-0.32	2.68	NOT SIG
0045KA	2031	2.23	2.01	2.45	SIG	1720	3.18	1.88	4.52	SIG
0055KA	1939	1.49	1.17	1.82	SIG	1627	1.50	-0.32	3.53	NOT SIG
0056KA	1834	-0.63	-0.90	-0.36	SIG	1560	-0.89	-2.94	1.00	NOT SIG
0057aKA	1809	2.35	2.09	2.62	SIG	1554	2.58	1.54	3.62	SIG
0057bKA	1840	2.29	2.04	2.56	SIG	1565	2.18	0.40	4.00	SIG
0062aKA	1410	1.49	1.24	1.75	SIG	1206	1.22	-0.26	2.81	NOT SIG
0062bKA	1336	2.07	1.80	2.35	SIG	1140	2.03	0.77	3.30	SIG
0065_or_66KA	1787	2.11	1.75	2.48	SIG	1512	1.15	-1.10	3.28	NOT SIG
0067_or_68KA	1787	1.46	1.03	1.91	SIG	1512	1.08	-0.70	3.00	NOT SIG
0069KA	1754	2.03	1.81	2.24	SIG	1486	2.12	0.44	3.82	SIG
0070KA	1695	0.96	0.43	1.45	SIG	1434	0.30	-1.70	1.95	NOT SIG
0071KA	1679	1.90	1.58	2.24	SIG	1415	1.23	-0.96	3.26	NOT SIG
0072KA	1729	1.73	1.50	1.97	SIG	1472	1.43	-0.15	2.83	NOT SIG
0073KA	1840	2.07	1.85	2.29	SIG	1565	2.34	1.19	3.58	SIG
0074KA	1702	1.77	1.51	2.04	SIG	1467	1.85	0.22	3.68	SIG
0075KA	1809	2.00	1.78	2.21	SIG	1554	3.47	1.89	5.46	SIG
0084KA	1663	1.75	1.53	1.97	SIG	1407	2.37	1.28	3.54	SIG
0087KA	1473	3.06	2.46	3.75	SIG	1252	2.28	-0.17	4.83	NOT SIG
0088KA	1540	1.77	1.45	2.11	SIG	1308	2.06	0.38	3.78	SIG
0091KA	1466	2.71	2.20	3.25	SIG	1248	2.18	0.29	3.94	SIG
0093KA	1948	3.02	2.52	3.58	SIG	1651	3.76	1.44	6.41	SIG
0095aKA	1277	2.25	1.97	2.54	SIG	1087	1.80	0.39	3.38	SIG
0095bKA	1262	0.90	0.66	1.15	SIG	1080	0.72	-0.76	2.26	NOT SIG
0096KA	1278	2.66	2.34	2.99	SIG	1087	2.13	0.68	3.50	SIG
0101KA	1872	1.06	0.86	1.26	SIG	1587	0.76	-0.79	2.25	NOT SIG
0107aKA	1517	2.64	2.30	2.99	SIG	1288	1.88	0.11	3.90	SIG
0107bKA	1834	-0.21	-0.47	0.07	NOT SIG	1560	1.06	-1.04	3.46	NOT SIG
0107cKA	1990	2.58	2.34	2.83	SIG	1675	2.79	0.89	5.02	SIG
0108KA	1541	1.11	0.82	1.41	SIG	1287	0.41	-1.19	1.87	NOT SIG
0110KA	1530	1.19	0.92	1.47	SIG	1302	1.64	0.80	2.58	SIG

0116KA	2226	0.67	0.49	0.85	SIG	1894	1.39	0.29	2.55	SIG
0121KA	1438	2.61	2.04	3.28	SIG	1234	0.95	-1.51	3.12	NOT SIG
0125KA	1265	2.67	2.07	3.34	SIG	1058	3.05	1.30	4.97	SIG
0126nKA	1500	0.48	0.23	0.75	SIG	1287	1.21	-1.02	3.48	NOT SIG
0144KA	1680	0.59	0.39	0.78	SIG	1423	0.03	-0.75	0.82	NOT SIG
0145KA	1697	0.84	0.63	1.03	SIG	1403	0.29	-0.97	1.49	NOT SIG
0147KA	1643	0.41	0.18	0.65	SIG	1343	-0.04	-0.83	0.79	NOT SIG
0166KA	2021	3.11	2.51	3.87	SIG	1714	2.76	1.11	4.40	SIG
0173KA	1872	2.11	1.90	2.32	SIG	1584	2.20	0.72	3.90	SIG
0174KA	1840	1.46	1.24	1.67	SIG	1572	1.62	-0.41	3.82	NOT SIG
0175KA	1477	3.21	2.56	3.96	SIG	1253	1.86	-0.32	4.04	NOT SIG
0176KA	1545	2.83	2.31	3.42	SIG	1310	1.38	-0.38	3.11	NOT SIG
0177KA	1801	2.52	2.26	2.81	SIG	1521	2.46	0.93	3.81	SIG
0178KA	1779	1.74	1.46	2.03	SIG	1517	1.34	-0.05	2.70	NOT SIG
0179aKA	1730	3.43	3.09	3.77	SIG	1464	2.64	0.55	4.49	SIG
0179bKA	1770	2.81	2.52	3.11	SIG	1497	2.92	1.16	4.51	SIG
0179cKA	1783	3.72	3.31	4.16	SIG	1523	4.95	2.61	7.37	SIG
0183KA	1871	3.17	2.83	3.51	SIG	1587	3.44	2.32	4.66	SIG
0228KA	1993	2.85	1.95	3.94	SIG	1691	3.55	1.48	6.03	SIG
0229KA	1993	1.13	0.94	1.31	SIG	1691	0.81	-0.24	1.89	NOT SIG
0230aKA	1688	0.62	0.42	0.82	SIG	1394	0.42	-0.30	1.08	NOT SIG
0230bKA	1872	1.91	1.70	2.11	SIG	1581	1.53	0.34	2.73	SIG
0230cKA	1738	0.52	0.33	0.71	SIG	1441	0.07	-0.90	0.81	NOT SIG
0230dKA	1688	0.62	0.42	0.81	SIG	1394	0.44	-0.30	1.16	NOT SIG
0230eKA	1872	2.26	2.02	2.50	SIG	1581	1.53	0.58	2.44	SIG
0230fKA	1738	0.52	0.33	0.72	SIG	1441	0.09	-0.75	0.88	NOT SIG
0231aKA	1688	0.22	-0.01	0.45	NOT SIG	1394	0.23	-0.56	1.00	NOT SIG
0231bKA	995	0.96	0.58	1.37	SIG	820	1.17	-0.04	2.51	NOT SIG
0232KA	1588	0.57	0.36	0.78	SIG	1315	0.44	-0.60	1.39	NOT SIG
0237aKA	1766	1.82	1.59	2.06	SIG	1493	1.91	1.17	2.72	SIG
0238aKA	1781	0.86	0.63	1.09	SIG	1476	1.50	0.68	2.39	SIG
0238bKA	1608	0.61	0.41	0.81	SIG	1345	0.37	-0.33	1.14	NOT SIG
0238dKA	1377	0.42	0.15	0.70	SIG	1149	0.21	-0.68	1.22	NOT SIG
0239aKA	1580	0.61	0.41	0.81	SIG	1300	0.19	-0.76	1.01	NOT SIG
0239bKA	1387	-0.31	-0.76	0.14	NOT SIG	1154	-0.25	-1.34	0.86	NOT SIG
0239cKA	1550	-0.25	-0.74	0.22	NOT SIG	1292	-0.99	-2.44	0.26	NOT SIG
0240aKA	1485	0.41	0.13	0.69	SIG	1244	-0.02	-1.52	1.36	NOT SIG
0245aKA	1835	0.40	0.18	0.62	SIG	1522	0.53	-0.13	1.20	NOT SIG
0245bKA	879	0.33	0.00	0.65	SIG	705	0.28	-0.67	1.32	NOT SIG
0247KA	1617	-0.06	-0.27	0.14	NOT SIG	1363	-0.11	-0.79	0.58	NOT SIG

0260KA	1609	-0.40	-0.65	-0.15	SIG	1359	-1.00	-2.86	0.57	NOT SIG
0276KA	1766	1.78	1.56	2.02	SIG	1493	1.87	1.10	2.73	SIG
0320KA	1529	2.32	2.08	2.57	SIG	1281	3.27	1.69	5.05	SIG
0328KA	2045	2.03	1.82	2.24	SIG	1733	2.71	1.55	3.99	SIG
0331aKA	2031	-0.31	-0.53	-0.10	SIG	1720	0.62	-0.85	2.40	NOT SIG
0331bKA	2044	1.53	1.32	1.75	SIG	1740	1.70	-0.23	3.50	NOT SIG
0332KA	2037	0.80	0.61	1.00	SIG	1726	2.69	1.12	4.38	SIG
0344KA	2071	4.53	4.15	4.91	SIG	1745	6.03	4.39	7.91	SIG
0356KA	1946	-0.12	-0.47	0.24	NOT SIG	1627	-0.33	-1.84	1.12	NOT SIG
0357KA	1943	1.37	0.88	1.90	SIG	1624	1.60	-0.68	3.85	NOT SIG
0365KA	1956	0.04	-0.24	0.34	NOT SIG	1644	-0.55	-2.07	0.98	NOT SIG
0386KA	2226	2.54	2.17	2.91	SIG	1894	2.65	1.57	3.93	SIG
0423KA	1470	2.73	2.33	3.16	SIG	1250	2.58	0.10	5.48	SIG
0439KA	1516	0.33	-0.08	0.71	NOT SIG	1274	0.14	-2.31	1.93	NOT SIG
0446KA	1603	2.14	1.91	2.36	SIG	1314	2.79	1.40	4.29	SIG
0454KA	1407	0.48	0.16	0.79	SIG	1181	-0.05	-1.27	1.12	NOT SIG
0489KA	1870	3.06	2.80	3.32	SIG	1560	2.70	1.29	4.04	SIG
0493aKA	1515	0.60	0.29	0.90	SIG	1253	0.95	-0.30	2.22	NOT SIG
0493bKA	1457	0.43	0.22	0.64	SIG	1202	0.59	-0.13	1.29	NOT SIG
0504aKA	1381	0.75	-0.01	1.62	NOT SIG	1169	1.16	-0.75	3.14	NOT SIG
0504bKA	1348	1.35	1.04	1.68	SIG	1137	-0.02	-1.66	1.42	NOT SIG
0506KA	329	1.65	0.35	2.92	SIG	233	2.27	-0.35	5.00	NOT SIG
0507KA	1297	1.94	1.60	2.32	SIG	1075	2.05	0.04	4.30	SIG
0511aKA	1788	3.68	2.44	5.28	SIG	1524	1.86	-1.97	5.06	NOT SIG
0511bKA	1753	1.97	1.43	2.60	SIG	1485	2.98	1.04	4.76	SIG
0513KA	2226	1.11	0.91	1.31	SIG	1894	0.87	0.27	1.49	SIG
0516KA	2226	2.50	2.27	2.73	SIG	1894	2.66	1.58	3.82	SIG
0517KA	1947	2.67	1.78	3.68	SIG	1650	2.38	0.40	4.59	SIG
0521KA	1627	2.29	2.06	2.54	SIG	1380	1.86	1.12	2.59	SIG
0564KA	1777	2.72	2.36	3.08	SIG	1505	3.44	2.27	4.81	SIG
0569KA	1993	0.49	0.31	0.66	SIG	1691	0.41	-0.52	1.38	NOT SIG
0572KA	2226	0.36	0.17	0.55	SIG	1894	0.16	-0.46	0.67	NOT SIG
0573KA	1745	-0.39	-0.62	-0.17	SIG	1475	-0.37	-1.18	0.49	NOT SIG
0577KA	1422	1.60	1.17	2.01	SIG	1172	1.74	0.36	3.20	SIG
0582KA	2226	0.81	0.60	1.02	SIG	1894	1.02	-0.29	2.46	NOT SIG
0612KA	1579	1.92	1.63	2.23	SIG	1318	1.31	0.23	2.37	SIG
0668KA	1541	0.27	0.07	0.49	SIG	1287	0.55	-0.87	1.95	NOT SIG
0798KA	1780	-1.08	-1.27	-0.88	SIG	1495	-0.50	-1.22	0.40	NOT SIG
0822KA	1237	0.89	0.55	1.25	SIG	1025	0.78	-0.51	1.95	NOT SIG
0837KA	1412	-0.03	-0.36	0.29	NOT SIG	1189	-0.07	-1.99	1.91	NOT SIG

0889aKA	2086	-0.61	-1.00	-0.23	SIG	1752	0.27	-1.58	2.15	NOT SIG
0889bKA	2086	0.86	0.67	1.06	SIG	1752	1.02	-0.24	2.25	NOT SIG
0917aKA	1551	0.56	0.26	0.87	SIG	1289	-0.32	-1.98	1.17	NOT SIG
0917bKA	1990	1.61	1.33	1.89	SIG	1675	3.29	1.55	5.44	SIG
0917cKA	1408	0.90	0.58	1.24	SIG	1201	0.73	-0.92	2.44	NOT SIG
0964aKA	1911	1.10	0.88	1.33	SIG	1610	0.49	-1.29	1.96	NOT SIG
0964bKA	1904	0.84	0.53	1.15	SIG	1602	0.80	-0.76	2.30	NOT SIG
0982KA	1108	0.67	0.41	0.94	SIG	935	1.14	0.06	2.18	SIG
0983KA	1140	1.62	1.35	1.88	SIG	953	1.19	-0.38	2.48	NOT SIG
1027aKA	1883	2.17	1.92	2.42	SIG	1589	2.02	0.38	3.75	SIG
1027bKA	1883	1.45	1.21	1.68	SIG	1589	1.73	0.38	3.30	SIG
1027cKA	1883	2.17	1.94	2.41	SIG	1589	1.58	0.65	2.52	SIG
1028aKA	1883	0.34	0.12	0.57	SIG	1589	0.37	-1.02	1.96	NOT SIG
1028bKA	1883	0.62	0.36	0.87	SIG	1589	0.58	-0.39	1.79	NOT SIG
1028cKA	1883	0.96	0.60	1.35	SIG	1589	0.42	-0.95	2.44	NOT SIG
1053KA	2226	2.24	2.03	2.45	SIG	1894	1.86	1.14	2.61	SIG
1111KA	1688	0.62	0.42	0.81	SIG	1394	0.25	-0.67	1.06	NOT SIG
1114KA	1003	1.84	1.54	2.15	SIG	828	1.92	0.24	3.75	SIG
1142aKA	1144	-0.14	-0.38	0.10	NOT SIG	946	-0.26	-1.45	1.05	NOT SIG
1142bKA	1144	0.28	0.03	0.52	SIG	946	1.03	-0.35	2.47	NOT SIG
1152KA	1426	-3.11	-3.45	-2.78	SIG	1219	-3.40	-5.10	-1.65	SIG
1163KA	1905	1.94	1.65	2.25	SIG	1622	1.89	0.94	2.74	SIG
1216KA	1838	1.38	1.19	1.58	SIG	1551	0.68	-0.59	2.05	NOT SIG
1267aKA	1548	1.58	1.29	1.89	SIG	1299	0.52	-1.59	2.34	NOT SIG
1267bKA	1548	2.23	1.86	2.62	SIG	1299	2.34	0.27	4.31	SIG
1334aKA	1367	0.29	-0.14	0.74	NOT SIG	1163	-0.90	-3.03	0.87	NOT SIG
1334bKA	1333	0.71	0.05	1.33	SIG	1134	-0.04	-2.37	1.79	NOT SIG
1334cKA	1311	2.21	0.97	3.71	SIG	1117	1.44	-0.73	3.78	NOT SIG
1341KA	1599	0.95	0.74	1.17	SIG	1335	1.61	0.54	2.62	SIG
1349KA	1638	0.83	0.59	1.06	SIG	1386	0.03	-1.30	1.32	NOT SIG
1372KA	1348	0.52	0.31	0.73	SIG	1139	0.57	-1.30	2.17	NOT SIG
1415KA	1595	1.14	0.92	1.35	SIG	1357	1.87	0.48	3.34	SIG
1427KA	1386	0.31	0.06	0.56	SIG	1159	0.10	-1.67	2.04	NOT SIG
1430KA	1404	0.45	0.23	0.67	SIG	1184	0.02	-0.78	0.74	NOT SIG
1431mKA	1744	-0.95	-1.15	-0.76	SIG	1493	-1.12	-2.20	-0.06	SIG
1431nKA	1744	-0.60	-0.83	-0.35	SIG	1493	-0.60	-1.38	0.17	NOT SIG
1434KA	1547	0.95	0.70	1.21	SIG	1309	0.74	-0.09	1.48	NOT SIG
1436KA	1628	-0.15	-0.35	0.05	NOT SIG	1381	0.00	-0.95	0.87	NOT SIG
1499KA	1447	1.21	0.67	1.78	SIG	1191	0.11	-1.82	2.23	NOT SIG
1531KA	1469	0.07	-0.23	0.34	NOT SIG	1235	0.77	-1.37	3.10	NOT SIG

1534KA	1551	0.76	0.41	1.10	SIG	1289	0.49	-1.31	2.27	NOT SIG
1537KA	1003	0.67	0.34	1.02	SIG	828	-0.01	-1.79	1.86	NOT SIG
1544KA	1543	0.69	0.28	1.15	SIG	1289	0.21	-2.28	2.59	NOT SIG
1545KA	1677	-1.87	-2.43	-1.37	SIG	1436	-1.49	-3.73	0.83	NOT SIG
1546aKA	1662	-0.62	-0.83	-0.41	SIG	1404	-0.87	-2.28	0.51	NOT SIG
1546bKA	1662	0.24	-0.06	0.53	NOT SIG	1404	-0.07	-1.56	1.50	NOT SIG
1548aKA	2086	0.74	0.50	0.99	SIG	1752	0.66	-0.73	2.13	NOT SIG
1548bKA	2086	0.80	0.53	1.08	SIG	1752	0.52	-1.07	1.95	NOT SIG
1549KA	1990	1.33	1.05	1.61	SIG	1675	1.27	-0.61	3.32	NOT SIG
1550KA	1517	2.48	1.65	3.51	SIG	1288	2.66	0.12	5.62	SIG
1559KA	1675	0.80	0.53	1.08	SIG	1400	1.05	-0.38	2.46	NOT SIG
1560KA	1697	0.30	0.00	0.59	SIG	1425	-0.15	-2.29	1.85	NOT SIG
1561KA	1776	-1.19	-1.48	-0.90	SIG	1496	-0.16	-1.70	1.28	NOT SIG
1586KA	1422	1.46	1.24	1.69	SIG	1200	1.00	-0.05	1.96	NOT SIG
1589KA	1453	0.87	0.64	1.10	SIG	1222	-0.50	-2.46	1.31	NOT SIG
1591KA	1259	1.43	1.09	1.79	SIG	1049	0.66	-0.70	1.90	NOT SIG
1592KA	1274	1.10	0.77	1.44	SIG	1068	0.89	-0.29	2.19	NOT SIG
1593KA	1017	0.56	0.10	1.05	SIG	834	0.48	-0.59	1.80	NOT SIG
1610KA	1973	0.02	-0.16	0.20	NOT SIG	1649	0.27	-1.04	1.49	NOT SIG
1611KA	2031	2.22	2.00	2.44	SIG	1720	3.20	1.93	4.57	SIG
1612aKA	1649	1.01	0.12	1.86	SIG	1407	-0.16	-2.96	1.89	NOT SIG
1612bKA	1614	-0.37	-0.63	-0.11	SIG	1360	0.12	-1.08	1.39	NOT SIG
1612cKA	1699	-1.53	-2.01	-1.09	SIG	1450	-2.18	-4.18	-0.48	SIG
1612dKA	1648	-0.63	-0.98	-0.33	SIG	1393	-1.00	-2.66	0.39	NOT SIG
1621KA	1296	0.65	-0.92	2.13	NOT SIG	1056	-0.11	-2.86	2.31	NOT SIG
1653KA	1761	0.53	0.34	0.73	SIG	1494	-0.02	-0.79	0.79	NOT SIG
1667aKA	1012	1.36	0.96	1.76	SIG	833	1.45	0.23	2.62	SIG
1667bKA	1003	0.67	0.32	1.02	SIG	828	0.30	-1.23	2.15	NOT SIG
1741KA	1523	1.66	1.30	2.05	SIG	1289	1.28	0.06	2.77	SIG
1827KA	1386	1.83	1.60	2.07	SIG	1181	0.79	-0.90	2.30	NOT SIG
1994aKA	1451	1.30	1.06	1.56	SIG	1214	1.51	-0.17	3.38	NOT SIG
1994bKA	1550	-0.57	-0.87	-0.27	SIG	1301	-1.17	-2.61	0.15	NOT SIG
1994cKA	1553	0.49	0.27	0.73	SIG	1306	0.46	-1.07	1.81	NOT SIG
1994dKA	1550	0.91	0.58	1.29	SIG	1299	1.59	0.31	3.00	SIG
2012KA	1223	0.51	0.21	0.83	SIG	1018	0.55	-0.54	1.76	NOT SIG

Table S4: Results from the *BayesTraits* analyses (columns 3-6) and the *brms* analyes controlling for genealogical and geographical relations (columns 7-10). Column 2 gives the sample size (n languages) and column 11 the support across both analyses.

code	m_n	BT_median_BF	BT_low_95_HDI	BT_upp_95_HDI	BT_HDI_SIG	SP_m_Estimate	SP_m_low_95_CI	SP_m_upp_95_CI	SP_SIG	supported
0001_or_2aKA	1958	49.89	39.65	61.07	SIG	3.00	1.79	4.29	yes	SIG
0001_or_2bKA	1881	52.88	45.26	68.88	SIG	1.03	-0.92	2.58	no	NOT SIG
0005KA	1787	-2.61	-15.68	8.15	NOT SIG	-0.04	-1.18	1.22	no	NOT SIG
0008KA	1772	11.42	3.16	27.01	NOT SIG	1.44	-0.08	2.90	no	NOT SIG
0015aKA	1941	30.05	24.91	43.32	SIG	2.12	1.02	3.28	yes	SIG
0015bKA	1926	20.92	13.79	38.68	SIG	1.01	-0.40	2.60	no	NOT SIG
0045KA	2031	120.12	106.94	132.43	SIG	3.81	2.32	5.36	yes	SIG
0055KA	1939	-0.34	-10.31	7.78	NOT SIG	1.38	-0.55	3.36	no	NOT SIG
0056KA	1834	-6.71	-14.88	11.66	NOT SIG	-1.25	-2.22	-0.24	yes	NOT SIG
0057aKA	1809	53.53	42.42	71.05	SIG	2.66	1.86	3.54	yes	SIG
0057bKA	1840	63.68	55.75	81.17	SIG	1.70	0.35	2.88	yes	SIG
0062aKA	1410	7.09	-0.02	17.08	NOT SIG	0.95	-0.10	2.00	no	NOT SIG
0062bKA	1336	16.34	9.19	31.75	NOT SIG	1.90	0.81	2.94	yes	NOT SIG
0065_or_66KA	1787	17.23	9.51	34.34	NOT SIG	2.00	0.52	3.39	yes	NOT SIG
0067_or_68KA	1787	18.14	10.33	24.70	SIG	1.02	-0.08	2.30	no	NOT SIG
0069KA	1754	40.96	31.14	56.97	SIG	1.78	0.48	3.20	yes	SIG
0070KA	1695	-7.67	-12.34	-2.90	NOT SIG	1.03	-0.73	2.57	no	NOT SIG
0071KA	1679	14.63	4.31	30.56	NOT SIG	2.17	0.90	3.54	yes	NOT SIG
0072KA	1729	21.43	13.18	34.98	SIG	1.61	0.12	2.83	yes	SIG
0073KA	1840	88.11	78.82	98.10	SIG	2.20	1.47	3.02	yes	SIG
0074KA	1702	31.52	23.11	39.78	SIG	1.99	0.70	3.36	yes	SIG
0075KA	1809	87.16	77.87	105.10	SIG	3.55	2.11	5.29	yes	SIG
0084KA	1663	60.92	51.43	77.07	SIG	2.68	1.23	4.15	yes	SIG
0087KA	1473	21.98	15.55	28.97	SIG	3.17	0.92	5.45	yes	SIG
0088KA	1540	15.89	10.03	22.70	SIG	1.52	-0.01	2.87	no	NOT SIG
0091KA	1466	19.01	9.75	31.45	NOT SIG	2.56	1.09	4.10	yes	NOT SIG
0093KA	1948	37.53	28.40	51.23	SIG	4.51	2.73	6.79	yes	SIG
0095aKA	1277	34.63	27.21	54.85	SIG	1.73	0.81	2.65	yes	SIG
0095bKA	1262	-4.66	-15.35	15.39	NOT SIG	0.66	-0.48	1.79	no	NOT SIG
0096KA	1278	39.68	31.76	46.11	SIG	2.06	1.02	3.03	yes	SIG
0101KA	1872	4.93	-3.12	18.83	NOT SIG	1.02	0.18	1.93	yes	NOT SIG
0107aKA	1517	33.40	25.59	59.01	SIG	2.36	0.96	3.86	yes	SIG
0107bKA	1834	4.74	-1.17	20.33	NOT SIG	0.03	-1.37	1.69	no	NOT SIG
0107cKA	1990	44.99	32.36	56.29	SIG	2.35	0.53	4.03	yes	SIG
0108KA	1541	19.46	10.78	27.38	SIG	0.83	-0.36	1.97	no	NOT SIG
0110KA	1530	-1.16	-6.40	14.30	NOT SIG	0.95	0.08	1.74	yes	NOT SIG

0116KA	2226	8.61	-7.50	29.84	NOT SIG	0.92	-0.31	2.17	no	NOT SIG
0121KA	1438	8.80	1.49	22.89	NOT SIG	1.74	-0.32	3.67	no	NOT SIG
0125KA	1265	24.14	15.72	40.10	SIG	2.00	0.62	3.32	yes	SIG
0126nKA	1500	-12.81	-21.05	6.49	NOT SIG	0.14	-1.98	2.19	no	NOT SIG
0144KA	1680	-2.47	-9.90	18.95	NOT SIG	-0.02	-0.62	0.58	no	NOT SIG
0145KA	1697	2.98	-3.73	11.94	NOT SIG	0.48	-0.98	1.65	no	NOT SIG
0147KA	1643	1.27	-4.51	20.12	NOT SIG	-0.14	-0.69	0.39	no	NOT SIG
0166KA	2021	40.65	31.19	49.47	SIG	3.06	1.83	4.45	yes	SIG
0173KA	1872	62.24	50.77	70.54	SIG	2.38	1.00	3.92	yes	SIG
0174KA	1840	13.17	0.80	31.78	NOT SIG	1.63	-0.15	3.72	no	NOT SIG
0175KA	1477	25.12	18.69	37.74	SIG	3.00	1.21	5.09	yes	SIG
0176KA	1545	27.24	18.07	43.81	SIG	2.33	0.99	3.88	yes	SIG
0177KA	1801	72.29	61.70	83.77	SIG	2.57	0.79	4.14	yes	SIG
0178KA	1779	13.43	5.70	30.98	NOT SIG	1.33	0.42	2.27	yes	NOT SIG
0179aKA	1730	141.92	130.15	153.33	SIG	3.83	2.79	5.00	yes	SIG
0179bKA	1770	125.13	110.16	147.14	SIG	4.08	2.96	5.44	yes	SIG
0179cKA	1783	170.62	162.09	179.93	SIG	5.51	3.54	7.69	yes	SIG
0183KA	1871	185.30	177.06	205.36	SIG	3.96	2.94	5.10	yes	SIG
0228KA	1993	8.09	-4.12	14.30	NOT SIG	2.28	0.41	4.12	yes	NOT SIG
0229KA	1993	18.90	9.86	29.43	NOT SIG	0.91	0.03	1.80	yes	NOT SIG
0230aKA	1688	13.59	2.14	22.72	NOT SIG	0.16	-0.64	0.69	no	NOT SIG
0230bKA	1872	41.05	30.81	52.28	SIG	1.38	0.46	2.34	yes	SIG
0230cKA	1738	8.12	-0.36	15.78	NOT SIG	-0.02	-0.89	0.64	no	NOT SIG
0230dKA	1688	13.29	3.17	23.14	NOT SIG	0.23	-0.40	0.75	no	NOT SIG
0230eKA	1872	26.31	17.60	44.42	SIG	1.64	0.79	2.52	yes	SIG
0230fKA	1738	8.00	0.14	19.25	NOT SIG	0.04	-0.67	0.68	no	NOT SIG
0231aKA	1688	-2.86	-7.88	6.12	NOT SIG	-0.03	-0.61	0.48	no	NOT SIG
0231bKA	995	-3.47	-10.40	2.46	NOT SIG	0.95	0.04	1.97	yes	NOT SIG
0232KA	1588	-1.52	-8.01	13.55	NOT SIG	0.26	-0.45	0.97	no	NOT SIG
0237aKA	1766	70.24	61.70	82.44	SIG	1.72	1.04	2.46	yes	SIG
0238aKA	1781	24.93	18.23	35.74	SIG	1.38	0.79	2.02	yes	SIG
0238bKA	1608	12.77	4.74	25.54	NOT SIG	0.46	-0.20	1.16	no	NOT SIG
0238dKA	1377	3.34	-5.51	10.06	NOT SIG	-0.16	-0.78	0.42	no	NOT SIG
0239aKA	1580	11.36	3.43	21.96	NOT SIG	0.15	-0.50	0.70	no	NOT SIG
0239bKA	1387	-3.42	-7.54	1.69	NOT SIG	-0.16	-1.17	0.80	no	NOT SIG
0239cKA	1550	-6.43	-11.16	-0.56	NOT SIG	-0.38	-1.44	0.68	no	NOT SIG
0240aKA	1485	-10.41	-16.86	2.02	NOT SIG	0.17	-1.03	1.30	no	NOT SIG
0245aKA	1835	-0.08	-8.07	4.13	NOT SIG	0.52	0.07	1.04	yes	NOT SIG
0245bKA	879	-3.56	-6.32	0.85	NOT SIG	0.27	-0.50	0.99	no	NOT SIG
0247KA	1617	0.41	-6.95	12.41	NOT SIG	-0.16	-0.73	0.39	no	NOT SIG

0260KA	1609	-15.47	-22.37	-7.28	NOT SIG	-0.20	-1.51	1.00	no	NOT SIG
0276KA	1766	68.36	58.94	80.60	SIG	1.68	0.98	2.43	yes	SIG
0320KA	1529	92.21	82.68	104.40	SIG	3.65	2.14	5.27	yes	SIG
0328KA	2045	87.47	77.04	102.33	SIG	3.48	2.13	5.11	yes	SIG
0331aKA	2031	-10.55	-16.86	-0.38	NOT SIG	0.56	-1.00	2.32	no	NOT SIG
0331bKA	2044	30.41	19.19	49.91	SIG	3.20	1.01	5.48	yes	SIG
0332KA	2037	18.58	10.29	33.53	SIG	2.87	1.38	4.45	yes	SIG
0344KA	2071	195.42	184.14	212.91	SIG	4.99	3.84	6.30	yes	SIG
0356KA	1946	-10.16	-14.52	9.03	NOT SIG	-0.05	-1.52	1.44	no	NOT SIG
0357KA	1943	7.68	-0.22	21.27	NOT SIG	1.94	-0.11	4.02	no	NOT SIG
0365KA	1956	-15.48	-24.47	-4.28	NOT SIG	-0.03	-1.17	1.14	no	NOT SIG
0386KA	2226	56.54	48.05	70.47	SIG	2.30	1.53	3.09	yes	SIG
0423KA	1470	31.54	23.01	44.93	SIG	3.13	1.24	5.16	yes	SIG
0439KA	1516	4.08	-1.89	12.05	NOT SIG	-0.13	-2.05	1.31	no	NOT SIG
0446KA	1603	69.51	62.21	84.36	SIG	1.96	0.73	3.25	yes	SIG
0454KA	1407	-7.93	-26.10	17.50	NOT SIG	-0.17	-1.11	0.68	no	NOT SIG
0489KA	1870	115.88	105.37	134.06	SIG	3.25	2.09	4.43	yes	SIG
0493aKA	1515	-8.60	-14.50	1.46	NOT SIG	1.08	0.15	2.09	yes	NOT SIG
0493bKA	1457	-2.05	-8.15	5.32	NOT SIG	0.64	0.14	1.20	yes	NOT SIG
0504aKA	1381	-8.50	-13.60	-4.73	NOT SIG	0.65	-0.75	2.06	no	NOT SIG
0504bKA	1348	-4.69	-13.03	6.74	NOT SIG	0.04	-1.05	1.02	no	NOT SIG
0506KA	329	1.74	0.52	2.48	NOT SIG	1.80	-0.49	4.05	no	NOT SIG
0507KA	1297	17.29	11.67	31.82	SIG	1.15	-0.72	2.71	no	NOT SIG
0511aKA	1788	16.66	14.49	19.65	SIG	2.06	-1.96	5.21	no	NOT SIG
0511bKA	1753	41.94	31.46	50.20	SIG	3.94	2.68	5.29	yes	SIG
0513KA	2226	36.53	20.99	65.13	SIG	1.04	0.45	1.70	yes	SIG
0516KA	2226	138.47	127.48	149.55	SIG	2.82	1.58	4.03	yes	SIG
0517KA	1947	16.10	12.90	18.82	SIG	3.01	1.66	4.77	yes	SIG
0521KA	1627	108.36	100.29	118.99	SIG	1.90	1.40	2.42	yes	SIG
0564KA	1777	106.67	99.14	115.02	SIG	3.31	2.49	4.22	yes	SIG
0569KA	1993	10.59	0.74	29.03	NOT SIG	-0.19	-0.83	0.45	no	NOT SIG
0572KA	2226	3.50	-19.67	54.26	NOT SIG	0.33	-0.08	0.72	no	NOT SIG
0573KA	1745	-7.47	-12.73	1.64	NOT SIG	-0.24	-0.82	0.34	no	NOT SIG
0577KA	1422	14.47	9.44	22.91	NOT SIG	1.54	0.43	2.48	yes	NOT SIG
0582KA	2226	14.96	7.41	22.58	NOT SIG	0.82	-0.32	1.87	no	NOT SIG
0612KA	1579	26.35	13.93	36.52	SIG	2.03	1.11	3.00	yes	SIG
0668KA	1541	-6.38	-12.82	7.80	NOT SIG	0.70	-0.38	1.83	no	NOT SIG
0798KA	1780	20.42	11.02	33.17	SIG	-0.41	-1.11	0.52	no	NOT SIG
0822KA	1237	5.07	0.62	8.64	NOT SIG	0.96	0.30	1.65	yes	NOT SIG
0837KA	1412	-13.70	-19.09	6.47	NOT SIG	0.28	-0.98	1.66	no	NOT SIG

0889aKA	2086	-4.65	-10.61	5.51	NOT SIG	-0.02	-1.49	1.49	no	NOT SIG
0889bKA	2086	-6.31	-14.96	6.73	NOT SIG	0.89	-0.24	2.10	no	NOT SIG
0917aKA	1551	-6.16	-16.97	4.39	NOT SIG	0.56	-0.79	1.91	no	NOT SIG
0917bKA	1990	6.68	-1.02	26.05	NOT SIG	2.63	1.42	4.10	yes	NOT SIG
0917cKA	1408	-0.15	-11.51	14.99	NOT SIG	0.32	-1.07	1.73	no	NOT SIG
0964aKA	1911	-0.70	-7.15	20.78	NOT SIG	0.63	-0.71	1.99	no	NOT SIG
0964bKA	1904	-5.94	-11.06	8.90	NOT SIG	0.92	-0.36	2.06	no	NOT SIG
0982KA	1108	0.92	-6.34	7.68	NOT SIG	0.94	0.13	1.82	yes	NOT SIG
0983KA	1140	21.62	12.85	36.90	SIG	0.79	-0.73	1.86	no	NOT SIG
1027aKA	1883	22.49	14.40	35.41	SIG	2.26	0.93	3.66	yes	SIG
1027bKA	1883	13.36	3.32	21.76	NOT SIG	0.97	0.15	1.74	yes	NOT SIG
1027cKA	1883	43.97	36.35	59.91	SIG	1.34	0.46	2.14	yes	SIG
1028aKA	1883	0.63	-7.74	12.80	NOT SIG	0.08	-1.77	1.44	no	NOT SIG
1028bKA	1883	-2.31	-10.67	6.21	NOT SIG	0.60	-0.23	1.44	no	NOT SIG
1028cKA	1883	18.15	6.41	30.99	NOT SIG	0.36	-0.89	1.69	no	NOT SIG
1053KA	2226	200.15	185.45	215.49	SIG	2.01	1.42	2.65	yes	SIG
1111KA	1688	13.93	4.22	23.95	NOT SIG	0.07	-0.77	0.69	no	NOT SIG
1114KA	1003	21.61	13.07	31.51	SIG	1.98	0.75	3.39	yes	SIG
1142aKA	1144	-10.72	-18.51	3.37	NOT SIG	-0.18	-1.04	0.76	no	NOT SIG
1142bKA	1144	-8.89	-19.16	3.55	NOT SIG	0.85	-0.09	1.85	no	NOT SIG
1152KA	1426	79.87	72.48	89.46	SIG	-2.62	-4.30	-0.83	yes	SIG
1163KA	1905	76.74	65.29	86.84	SIG	1.96	1.05	2.78	yes	SIG
1216KA	1838	15.59	7.14	30.19	NOT SIG	0.53	-0.38	1.40	no	NOT SIG
1267aKA	1548	8.99	1.96	15.64	NOT SIG	1.10	-0.37	2.40	no	NOT SIG
1267bKA	1548	28.30	21.60	39.41	SIG	2.39	0.82	3.88	yes	SIG
1334aKA	1367	-2.32	-9.58	6.06	NOT SIG	0.03	-1.65	1.61	no	NOT SIG
1334bKA	1333	-11.23	-15.58	-7.38	NOT SIG	0.53	-1.14	1.96	no	NOT SIG
1334cKA	1311	-0.96	-5.61	4.57	NOT SIG	1.74	-0.29	4.03	no	NOT SIG
1341KA	1599	4.58	-5.21	14.78	NOT SIG	1.22	0.26	2.13	yes	NOT SIG
1349KA	1638	-5.37	-13.07	7.70	NOT SIG	-0.11	-1.14	0.91	no	NOT SIG
1372KA	1348	-6.91	-12.17	7.79	NOT SIG	0.34	-1.07	1.54	no	NOT SIG
1415KA	1595	24.56	17.54	40.92	SIG	1.60	0.75	2.53	yes	SIG
1427KA	1386	9.66	1.69	20.03	NOT SIG	-0.65	-1.52	0.39	no	NOT SIG
1430KA	1404	-11.73	-20.71	2.17	NOT SIG	0.08	-0.59	0.72	no	NOT SIG
1431mKA	1744	30.99	22.19	43.41	SIG	-0.98	-1.78	-0.26	yes	SIG
1431nKA	1744	-1.44	-9.18	6.15	NOT SIG	-0.91	-1.59	-0.24	yes	NOT SIG
1434KA	1547	1.38	-3.85	12.02	NOT SIG	0.27	-0.69	1.00	no	NOT SIG
1436KA	1628	-16.59	-23.40	-3.35	NOT SIG	0.31	-0.94	1.55	no	NOT SIG
1499KA	1447	3.77	-3.99	13.82	NOT SIG	-0.07	-1.76	1.63	no	NOT SIG
1531KA	1469	-7.33	-20.10	5.00	NOT SIG	0.44	-0.90	1.99	no	NOT SIG

1534KA	1551	-5.99	-12.53	3.32	NOT SIG	0.29	-1.09	1.70	no	NOT SIG
1537KA	1003	4.83	-6.60	12.58	NOT SIG	0.39	-1.09	1.93	no	NOT SIG
1544KA	1543	-8.89	-13.28	0.96	NOT SIG	0.98	-0.58	2.68	no	NOT SIG
1545KA	1677	8.35	0.56	18.11	NOT SIG	-2.16	-4.23	-0.20	yes	NOT SIG
1546aKA	1662	-4.47	-10.79	7.27	NOT SIG	-0.54	-1.66	0.50	no	NOT SIG
1546bKA	1662	-13.66	-19.40	1.62	NOT SIG	-0.16	-1.29	1.01	no	NOT SIG
1548aKA	2086	4.67	-6.69	14.18	NOT SIG	0.22	-1.17	1.59	no	NOT SIG
1548bKA	2086	-8.14	-12.38	-0.83	NOT SIG	0.14	-1.12	1.37	no	NOT SIG
1549KA	1990	17.62	10.15	28.00	SIG	1.37	-0.01	2.85	no	NOT SIG
1550KA	1517	2.84	-3.71	9.01	NOT SIG	2.62	0.67	4.89	yes	NOT SIG
1559KA	1675	6.07	-1.77	18.05	NOT SIG	0.81	-0.17	1.82	no	NOT SIG
1560KA	1697	-15.70	-22.64	-5.33	NOT SIG	0.08	-1.25	1.29	no	NOT SIG
1561KA	1776	-5.86	-11.59	3.18	NOT SIG	-0.63	-1.74	0.44	no	NOT SIG
1586KA	1422	16.22	10.02	27.39	SIG	1.20	0.28	1.96	yes	SIG
1589KA	1453	-9.54	-16.17	6.14	NOT SIG	-0.28	-1.82	1.07	no	NOT SIG
1591KA	1259	22.02	9.66	29.11	NOT SIG	1.34	0.23	2.38	yes	NOT SIG
1592KA	1274	7.69	0.02	16.29	NOT SIG	0.86	0.09	1.71	yes	NOT SIG
1593KA	1017	-1.58	-5.56	3.34	NOT SIG	0.48	-0.33	1.47	no	NOT SIG
1610KA	1973	-8.16	-17.08	7.02	NOT SIG	0.45	-0.70	1.58	no	NOT SIG
1611KA	2031	120.04	107.21	134.03	SIG	3.80	2.31	5.36	yes	SIG
1612aKA	1649	-1.96	-4.59	0.50	NOT SIG	0.82	-0.90	2.14	no	NOT SIG
1612bKA	1614	0.67	-8.38	9.23	NOT SIG	0.15	-0.89	1.16	no	NOT SIG
1612cKA	1699	-7.47	-19.04	2.96	NOT SIG	-1.84	-3.59	-0.30	yes	NOT SIG
1612dKA	1648	-7.13	-14.70	3.60	NOT SIG	-1.19	-2.88	0.33	no	NOT SIG
1621KA	1296	-5.50	-9.55	-3.01	NOT SIG	0.84	-1.01	2.65	no	NOT SIG
1653KA	1761	-11.42	-18.01	6.03	NOT SIG	0.28	-0.41	1.02	no	NOT SIG
1667aKA	1012	18.23	9.40	25.29	NOT SIG	1.49	0.37	2.45	yes	NOT SIG
1667bKA	1003	3.98	-4.85	11.79	NOT SIG	0.39	-1.01	1.72	no	NOT SIG
1741KA	1523	28.74	18.22	37.95	SIG	1.87	0.64	3.33	yes	SIG
1827KA	1386	9.23	1.18	27.71	NOT SIG	0.71	-0.88	2.05	no	NOT SIG
1994aKA	1451	15.27	8.88	33.57	NOT SIG	1.05	-0.12	2.11	no	NOT SIG
1994bKA	1550	-10.32	-16.39	-2.03	NOT SIG	-0.94	-1.85	-0.08	yes	NOT SIG
1994cKA	1553	-8.43	-18.59	-0.99	NOT SIG	0.15	-1.40	1.38	no	NOT SIG
1994dKA	1550	-9.87	-16.49	9.56	NOT SIG	1.37	0.44	2.37	yes	NOT SIG
2012KA	1223	-10.93	-17.59	5.84	NOT SIG	0.65	-0.41	1.79	no	NOT SIG

Supplementary Information Text 5: Extended statistical methods

This paper features two kinds of statistical models: (i) generalized linear mixed effects models (GLMMs; implemented using the *R*-package *brms,* Bürkner 2017) and (ii) covarion models (implemented in *BayesTraits*, Pagel 1994, Pagel and Meade 2006). GLMMs allow us to test the strength of the relationship between the Grambank features that are implicated by the suggested universal, all the while introducing controls for phylogenetic and spatial non-independence. We included three different GLMMs per universal: a) with control for spatial and phylogenetic non-independence (estimated by language locations and a global phylogeny), b) without any controls (henceforth 'naïve') and c) with control for phylogenetic non-independence via family membership only. Secondly, the covarion modeling in *BayesTraits* allows us to test not only if the relationship between the traits holds strong also when other relevant factors are accounted for, but also explicitly models the traits diachronically on the trees and compares the fit when the model assumes they are dependent on each other to a model assuming that they are independent. This section of the Supplementary Information outlines more details of these models.

PGLMMs in *brms*: spatiophylogenetic correlations.

We constructed phylogenetic generalized linear mixed effects models (PGLMMs) using the package *brms* (Bürkner 2017) in R (R Core Team 2023) in order to test whether the proposed universals hold up after controlling for genealogical and geographical relations. The *brms* package allows us to fit Bayesian multilevel models in *R* using the probabilistic programming language STAN (Carpenter et al. 2017). We choose *brms* because it allows us to include categorical (binary) variables as well as random effects that control for genealogical and spatial relations (Bürkner 2023). Both the response variable (the condition part of the universal) and the main fixed effect (the result part of the universal) are binary. We use the following formula with the logistic link function:

$$y_i \sim Bernoulli(p_i)$$
$$logit(p_i) = b_0 + b_1 x + b_{macroarea} x + \gamma_{macroarea} + e_p + e_s + e_r$$
$$e_p \sim N(0, \sigma_p^2 P)$$
$$e_s \sim N(0, \sigma_s^2 S)$$
$$e_r \sim N(0, \sigma_r^2 R)$$

This formula represents a PGLMM (phylogenetic generalized linear mixed model), where b_0 is an intercept, b_1 is a slope, and x is a predictor variable. Macroarea (a categorical variable; taken from Glottolog v. 4.3 (Hammarström et al. 2020)) is treated as a random effect, whereby its intercepts ($\gamma_{macroarea}$) and x slopes ($b_{macroarea}x$) are estimated (c.f. Sinnemäki 2010). These models have three types of random effects (residual errors): phylogenetic effects (e_p), spatial effects (e_s), and residual (e_r) effects. Each of these follow a multivariate normal distribution with mean 0 and variance equal to the product of the respective standard deviation value (σ^2)

and the variance-covariance matrix. *R* represents an identity matrix, where all diagonal elements are equal to 1 and non-diagonal elements equal to zero. Phylogenetic and spatial variance-covariance matrices are built based on the Brownian motion evolution and Matérn covariance function, respectively (see also Claessens et al. 2020, Guzmán Naranjo and Becker 2022, Shcherbakova et al. 2023 for details). The phylogenetic variance-covariance matrix was calculated using the posterior trees from Bouckaert et al. (2022); the spatial variance-covariance matrix was calculated using longitude and latitude from Glottolog v. 4.3 (Hammarstöm et al. 2020). Our approach is similar to that of Guzmán Naranjo & Becker (2022), who also use a correlation matrix in their Bayesian GLMM to deal with genealogical autocorrelation. They deal with spatial autocorrelation in a different fashion, however, namely through modeling Gaussian Processes on macro-areas.

Further details on the models can be found in SI Data 3. We used default priors for the main independent variable (the second part of the universal); `student_t(3, 0, 2.5)` for the coefficient and `exponential(1)` for its standard deviation. One of the sampling parameters was slightly modified to facilitate convergence (adapt_delta = 0.99). Convergence was assessed by checking parameters generated by *brms*, Rhat and ESS (Effective Sample Size), making sure that they reach values that indicate chain convergence (also for the other *brms* models described below). In order to account for phylogenetic uncertainty, we ran the model 100 times, each time using a different phylogeny taken from Bouckaert et al.'s (2022) posterior sample (originally, their posterior has 902 trees) to calculate the phylogenetic variance-covariance matrix. We then summarize the relevant statistics from the model (such as the main fixed effect and its 95% Credible Interval (95% CI)) using the median.

During analysis, we discovered a problem with the function from the *R*-package which computes the spatial variance-covariance matrix (vcv; *geoR*; Ribeiro et al 2024). The issue concerns how the antimeridian and curvature of the earth is handled when the original distance matrix is calculated, which is then the input for creating the vcv. When users choose to let the function *geor::varcov.spatial()*, compute the distance matrix directly and then perform subsequent calculations for the vcv, the function treats the longitude and latitude points as if they are on a two-dimensional rectangular surface. This means that the three-dimensional nature of the globe is ignored. This has consequences primarily for pairs of points which straddle the antimeridian. For example, Fiji and Samoa are on either side of the antimeridian in the Pacific Ocean and the function calculates a distance that is almost the entire circumference of the earth - instead of the as-the-crow-flies distances of approximately 1,200 km. Another consequence of treating longitude and latitude positions as two-dimensional is that the curvature of the earth is not taken into account. These two problems are both mitigated by the spatial decay function of the later vcv-calculation which downweights covariance of pairs further apart. We assume (similarly to Skirgård et al 2023; Dinnage et al 2020) that the covariance effect of geographic pairwise distance is not linear. For example, points which are 1,000 km apart are having a similar effect

on each other as points which are 10,000 km apart (that is to say, very little effect). The calculation of the spatial vcv by *geoR* takes this into account by including a smoothness parameter, which dictates the decay of the effect of spatial distance. Because of this, the problems with the distance-calculation in *geoR* are mainly an issue for pairs of islands which straddle the antimeridian (such as Fiji and Samoa).

To understand the effect of this problem we re-ran the *brms* analyses again using the MCC tree from Bouckaert et al. (2022) but with corrected distances (using the function *rdist.earth()* in the R-package *fields*, Nychka et al 2015). The analyses are included in SI data 1, columns 'COVAR_Estimate'; 'COVAR_low_95_CI'; 'COVAR_upp_95_CI'; 'COVAR_SIG'. We found no effect on the ultimate outcome of the analysis, as only very few language pairs are affected. In terms of absolute support for the universals, these analyses match the phylospatial *brms* analyses using 100 trees exactly except for three universals that are supported in these *COVAR* analyses. This is simply due to the fact that using 100 trees instead of the MCC posits a more stringent test of support. Correlation analyses of the main effect (coefficient) estimates of the phylospatial *brms* analyses and these *COVAR* analyses reveal that they are highly similar (Spearman's $r = 0.997$ (189 degrees of freedom), $P < 0.001$, 95% CI 0.997–0.998).

GLMMs in *brms*: naïve analyses.
In order to show the need for the spatiophylogenetic models central to our study, we wished to demonstrate the level of support for the tested universals without any considerations for autocorrelation. To this end, we constructed another set of GLMMs with *brms* (Bürkner 2017) in R (R Core Team 2023), which did not include any random effects (part of SI Data 3):

$$y_i \sim Bernoulli(p_i)$$
$$logit(p_i) = b_0 + b_1 x + e_r$$
$$e_r \sim N(0, \sigma_r^2 R)$$

GLMMs in *brms*: robustness analyses.
In order to demonstrate the need for modeling which controls for spatiophylogenetic effects, we test whether the supported universals hold without the maximum clade credibility tree from Bouckaert et al. (2022). To this end, we constructed another set of GLMMs with *brms* (Bürkner 2017) in *R* (R Core Team 2023), which used language family membership as a categorical control instead of the full continuous phylogeny (part of SI Data 3). This is common in modern typology (Bentz and Winter 2013, Coupé 2018, Sinnemäki and Di Garbo 2018, Josserand et al. 2021, Dediu 2023). For these analyses, data on language families was taken from Glottolog v. 4.3 (Hammarstöm et al. 2020). Language family membership is a categorical variable construed as a random effect, both intercepts and slopes were estimated (c.f. Sinnemäki 2010). Only

languages from families with five or more members were included. The model aims to capture the potential effects of language families: (γ_{family}) and x slopes ($b_{family}x$):

$$logit(p_i) = b_0 + b_1 x + b_{macroarea} x + \gamma_{macroarea} + b_{family} x + \gamma_{family} + e_s + e_r$$
$$e_s \sim N(0, \sigma_s^2 S)$$
$$e_r \sim N(0, \sigma_r^2 R)$$

***BayesTraits*: Evolutionary dynamics on a phylogenetic tree.**

To model the evolutionary dynamics of the universals that were found to be relevant in the spatiophylogenetic PGLMMs, we used *BayesTraits* (Pagel 1994, Pagel and Meade 2006). We mapped our grammatical data onto the Bouckaert et al. (2022) phylogenies using a continuous-time Markov model of trait evolution implemented as the *Discrete* model in *BayesTraits* (c.f. Dunn et al. 2011). All code and results can be found in SI Data 2, Table S4 presents the main statistics). This model is specifically used to test if two binary traits are correlated. Our data is binary: in testing Greenberg's (1963) universal, "In languages with prepositions, the genitive almost always follows the governing noun", we have two binary traits that take two states each:

1. Adposition-Noun order (coded 1) and its inverse, ¬Adposition-Noun order (coded 0);
2. And Noun-Genitive order (coded 1) and its inverse, ¬Noun-Genitive order (coded 0).

While the inverse of adposition-noun order will nearly always be noun-adposition order, we have to account for languages with no adpositions, for example, so this is formally how the Grambank data were treated (see Supplementary Information Text 2 and 3). The *Discrete* model in *BayesTraits* operates by comparing two models: one is called the independent model, and the other, the dependent model (see Figure S3). In the independent model, the two traits evolve independently, in other words, the transition from state 1 to state 0 (and vice versa) in trait A is independent of the state of trait B. Likewise, change in trait B is independent of change in trait A. Trait A is the condition part of the universal, in the example above, whether the language has adposition noun order (state 1) or not (state 0), and the result part of the universal is trait B, relating the order of noun and genitive in the example above. Since the two traits evolve (change) on the branches of the phylogenetic tree separately, the independent model has four rate parameters (in Figure S3 they are labeled with q_{01} and q_{10}, but they are estimated separately).

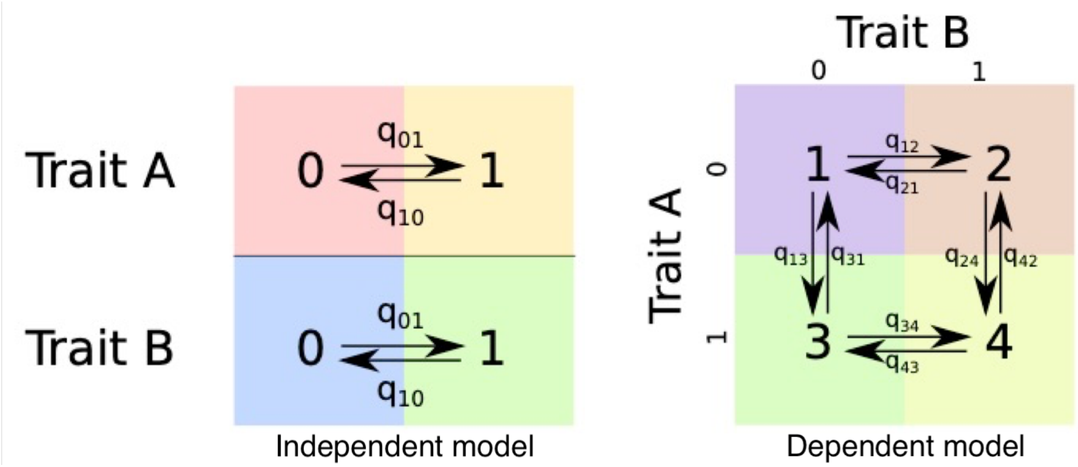

Figure S3: Illustration of the evolutionary modeling of traits in Discrete*'s independent and dependent model (see Pagel and Meade 2024). Colors refer to distinct states.*

In the dependent model, in contrast, the two traits evolve in a dependent manner, that is, the transition from state 1 to state 0 (and vice versa) in trait A is dependent on the state of trait B, and vice versa. Instead of four rate parameters, the dependent model has eight: two rate parameters each to change between four states 1, 2, 3, and 4. State 1 is when both traits A and B are in state 0, so in our example, ¬adposition-noun order & ¬noun-genitive order. State 4 is when both traits A and B are in state 1, so in our example, the proposed configuration of the universal: adposition-noun order & noun-genitive order. In states 2 and 3, either trait A or trait B is 1, while the other is 0. Changes in which two changes happen at the same time, for example from state 1 (trait A: 0; trait B: 0) to state 4 (trait A: 1; trait B: 1) are not allowed. In the dependent model, estimates for these eight rate parameters and ancestral state estimates are generated. These rate parameters are often written down in a cross-tabulation (called a Q-matrix), with state combinations on the columns and rows, with rate parameters listed in cells so that the association between state combinations and rate parameters is made clear (see Pagel and Meade 2024).

The statistical test that *Discrete* allows us to do, then, is to compare the evolution of two binary traits: once as if both traits have nothing to do with one another, in the independent model, and once as if both traits are correlated, in the dependent model. The two models are compared to see which fits the data better, hence forming a formal test of the correlation of two traits as their evolution is modeled on the branches of the phylogenetic tree set.

In order to make sure that *BayesTraits* assesses both models on a big sample of trees, and hence captures the uncertainty present in the tree sample, we force BayesTraits to burn-in on all trees with the *EqualTrees* command. In order to keep this computationally tractable, we randomly downsample the set of phylogenies from Bouckaert et al. (2022) from 902 posterior trees to 100 trees. Convergence of the Markov chains is assessed by looking at the so-called 'caterpillar plot' of iteration number (x axis) against log-likelihood (y axis). The reason for not using the Pearson

or Spearman correlation between iteration number and log-likelihood is that the log file holds information about all phylogenies, and is hence not stable across them. Model fit is assessed using log marginal likelihoods, estimated using stepping stone sampling (Xie et al. 2011). We calculate the 95% High posterior Density Interval (95% HDI) and the median of the posterior marginal likelihoods; model fits of the dependent and independent model of trait evolution are then compared using natural log Bayes Factors (BF) (Raftery 1996: 165):

log Bayes Factors = log marginal Lh dependent model – log marginal Lh indep. model

A positive Bayes factor indicates that the dependent model performs better than the independent model, providing evidence for correlation of the two traits. Given that we are testing for multiple correlations, we use a conservative cut-off for evaluating the 95% HDI for Bayes Factor support. When the lower bound of the 95% HDI > 10, we consider that definitive support for the dependent model, and hence, for correlated evolution.

***BayesTraits*: Analysis of harmonicity.**

We next tested if the state configuration predicted by the universal is an evolutionary attractor. If so, rates of change should show a bias for changing towards predicted state configurations. This idea is rooted in, but not identical to, ideas about so-called 'harmony' expressed by Greenberg (1963) and further discussed by Croft (2003) and others. The idea is that for a universal such as "In languages with prepositions, the genitive almost always follows the governing noun" (Greenberg 1963), adposition-noun order is harmonic with noun-genitive order, as adposition-noun order hardly occurs with genitive-noun order. Hence, harmony is mostly frequency-based in the sense that correlated word orders in the world's languages are said to be harmonic; however, note that the correlated orders also have the same structure: the head is followed by the dependent. Because these word order patterns engage with others, for instance the order of noun and other modifiers, and the order of verb and object, a complex set of harmonic word order universals emerges, that has been investigated often during the last decades (see Croft 2003).

We use the terms 'harmony' and 'harmonic' to refer to the state configuration predicted by the universal, both for word order universals as for hierarchical universals. We do this in order to (indirectly) refer to the large body of literature that has reflected on the special (interconnected) status of these generalizations. The use of the terms 'harmony' and 'harmonic' easily extends to hierarchical universals as here we also have 'disharmonic' states: states in which one of the traits is lacking.

We tested if rates of change show a bias for changing towards predicted state configurations – harmonic ones – as compared to disharmonic ones. Figure S3 above illustrates how these analyses are implemented in *BayesTraits*. In the dependent model, state 4 (1,1) is harmonic, while state 2 (0,1) and state 3 (1,0) are disharmonic. State 1 (0,0) does not concern us here, as

diachronic change to state 4 always goes through state 2 or 3. We take those universals (n=60) for which the dependent model is a better fit with the lower bound of the 95% HDI interval > 10, and disregard the rest of the universals. To identify if these showed a tendency towards evolving into harmonic states vs. disharmonic states we extracted rate estimates from these analyses. We categorized a subset of the rates in the Q matrix as either leading to a harmonic state (i.e.to state 4) or to a disharmonic state (i.e. to state 2 or 3). As shown in Figure S3, for example, rate q34 is the rate at which languages evolve from having trait A without trait B (i.e. 1,0) into having both features (1,1) and is therefore harmonic. The opposite rate, q43, is the rate at which languages evolve from having both features (1,1) to having just the first feature (1,0) and is therefore disharmonic. For each of these pairs of rates, in each generation of the MCMC chain, we asked if the harmonic rate was larger than the disharmonic rate i.e. if there is a stronger tendency towards harmonic state patterns. The results are displayed in Figure 4 in the main text and discussed in the relevant section of the Results.

Methodological conceptualization.

In this paper, we demonstrate the usage of two models, one that accounts for both spatial and phylogenetic autocorrelation (PGLMMs in *brms* in R) and one that controls for phylogenetic autocorrelation by explicitly modeling change on the branches of a tree (*BayesTraits*). However, it should be noted that these two models are very different.

Given the nature of our binary response and predictor variables, the PGLMMs use the *Bernoulli* distribution and the *logit* link; conceptually, this is very close to logistic regression. This implies that the relationship between the response and predictor variables is assessed in terms of log-odds that, during estimation, are allowed to take a continuous random walk. Additionally, it is abundantly clear that phylogenetic and spatial autocorrelation need to be taken into account (Cysouw 2005, Ladd et al. 2014, Bromham et al. 2018, Hartmann 2022, Bromham 2023, Bromham and Yaxley 2023, Shcherbakova et al. 2023). Languages tend to share features due to shared histories, either due to genealogical relatedness or through language contact, the change of which increases with spatial proximity. Hence, phylogenetic and spatial autocorrelation are assessed in the error terms in our *brms* analyses.

The analysis in *BayesTraits,* however, models discrete trait evolution as a continuous time Markov chain (CTMC; Dunn et al. 2011, Cysouw et al. 2011, Cathcart 2018, Jäger and Wahle 2021). Change in the discrete variables is modeled on the branches of the phylogenetic tree, with each change being independent of past or future states the variable was in. The association between the two variables is assessed through model comparison of the dependent and independent models (see Figure S3), not through an direct assessment of their association in terms of log-odds, as in the PGLMMs. The other main difference between the analyses is that they take into account phylogenetic autocorrelation in very different ways, and that the *BayesTraits* analyses do not take spatial autocorrelation into account at all.

We strongly believe that the fact that these analyses, despite their conceptual differences, find support for a set of universals with considerable overlap is indicative of their appropriateness and, more importantly perhaps, of the validity of our results.

Supplementary Information Text 6: Comparison of different models

Naïve model vs. spatiophylogenetic model.
In the main text, we show that a naïve model, a Bayesian generalized linear mixed effects model without controlling for genealogical and geographical relations, finds support for 174/191 (91%) of the universals. In Figure 1 in the main text and Figure S4 below, these results are compared to the *brms* spatiophylogenetic models, which correct for spatial and phylogenetic confounds using covariance matrices (see Methods and Supplementary Information Text 5; see SI Data 1 and Tables S3 and S4 for sample sizes and estimates). The number of supported universals decreases substantially to 89/191 (47%). This is shown for all universals in Figure S4. Figure S5 additionally shows all the main fixed effect estimates and their 95% CI intervals; for all the 100 analyses conducted on individual trees from Boouckaert et al. (2022).

Interestingly, some universals that are supported in the spatiophylogenetic models (i.e. the median of the 95% credible interval of the main coefficient estimate excludes zero) fall entirely below zero, implying a negative relationship between the condition and the implication of the universal. This is the case for six universals:

1) V-initial ⇒ N-Dem-Num-Adj (1545KA, "In verb-initial languages, the demonstrative, numeral, and qualifying adjective follow the common noun in that order or its mirror image (Adj + Num + Dem)", unpublished statements of Keenan's, reproduced in Payne 1990). Does not hold in the coevolution analyses performed in *BayesTraits*.
2) VSO ⇒ N-Adj (0056KA, "With overwhelmingly more than chance frequency, languages with dominant order VSO have the adjective after the noun", Greenberg 1963). Does not hold in the coevolution analyses performed in *BayesTraits*.
3) Rel-N ⇒ N-Rel (1152KA, "For any language, preposed relative clause implies postposed relative clause", Moravcsik 1969). Holds in the coevolution analyses performed in *BayesTraits*.
4) SOV& N-Num ⇒ N-Num agr (1612cKA, "In SOV languages, when the expected order of Modifier-Noun does not occur, agreement [between noun and modifier] tends to appear", Foster & Hofling 1987). Does not hold in the coevolution analyses performed in *BayesTraits*.
5) less case ⇒ more tense & more case ⇒ less tense (1431mKA & 1431nKA, "The more developed a case system is, the less is its system of verbal tenses", Serebrennikov 1974). Only 1431mKA, less case ⇒ more tense, holds in the coevolution analyses performed in *BayesTraits*.
6) non-affixed Gen-N ⇒ OV (1994bKA, standardized "IF the attributive noun (genitive, esp. when morphologically unmarked) precedes the head noun, THEN [...] (v) direct objects (accusative, esp. when morphologically unmarked) precede the verb", original in

German; Schmidt 1926: 381 et sqq, Schmidt 1903: 381-383). Does not hold in the coevolution analyses performed in *BayesTraits*.

As for (1); we tested a weaker version of the universal, namely whether the three mentioned modifiers follow the noun, as Grambank does not have information on word order dependencies between modifiers. The support for this universal implies that there is evidence that in fact these three modifiers are NOT all placed after the noun in verb-initial languages. This relates to the second universal for which we find support for a negative coefficient, VSO ⇒ N-Adj, showing that verb-initial languages tend to NOT have noun-adjective order; (1) and (2) can be tentatively interpreted as evidence for the association of verb-initial languages with adjective-noun order and possibly other prenominal modifiers. As for (3), here we refute Moravcsik's (1969) strongly formulated claim 'For any language, preposed relative clause implies postposed relative clause' - there is in fact evidence for the opposite direction, i.e., languages with preposed relative clauses tend to NOT have postposed relative clauses.

In (4), we test one implementation of Foster & Hofling's (1987) claim, 'In SOV languages, when the expected order of Modifier-Noun does not occur, agreement [between noun and modifier] tends to appear'. We implemented this for four adnominal agreement targets, and it holds for none, suggesting that adnominal agreement is not linked to noun-modifier order in SOV languages. However, for this specific implementation, the negative coefficient suggests there is evidence for SOV and noun-numeral languages to not have agreement between the noun and the numeral; reflecting that such agreement is not common anyway and especially not so in SOV languages. In (5) we have two implementations of the same universal, both have supported negative coefficients, suggesting that the relationship between case marking and tense marking is inverse from Serebrennikov's (1974) proposal. We still count these universals among those we find support for, because a correlation is attested, albeit in the opposite direction than proposed. In (6), there is limited evidence for non-affixed Gen-N phrases to be associated NOT with with OV order, but rather with verb-inital or verb-medial order.

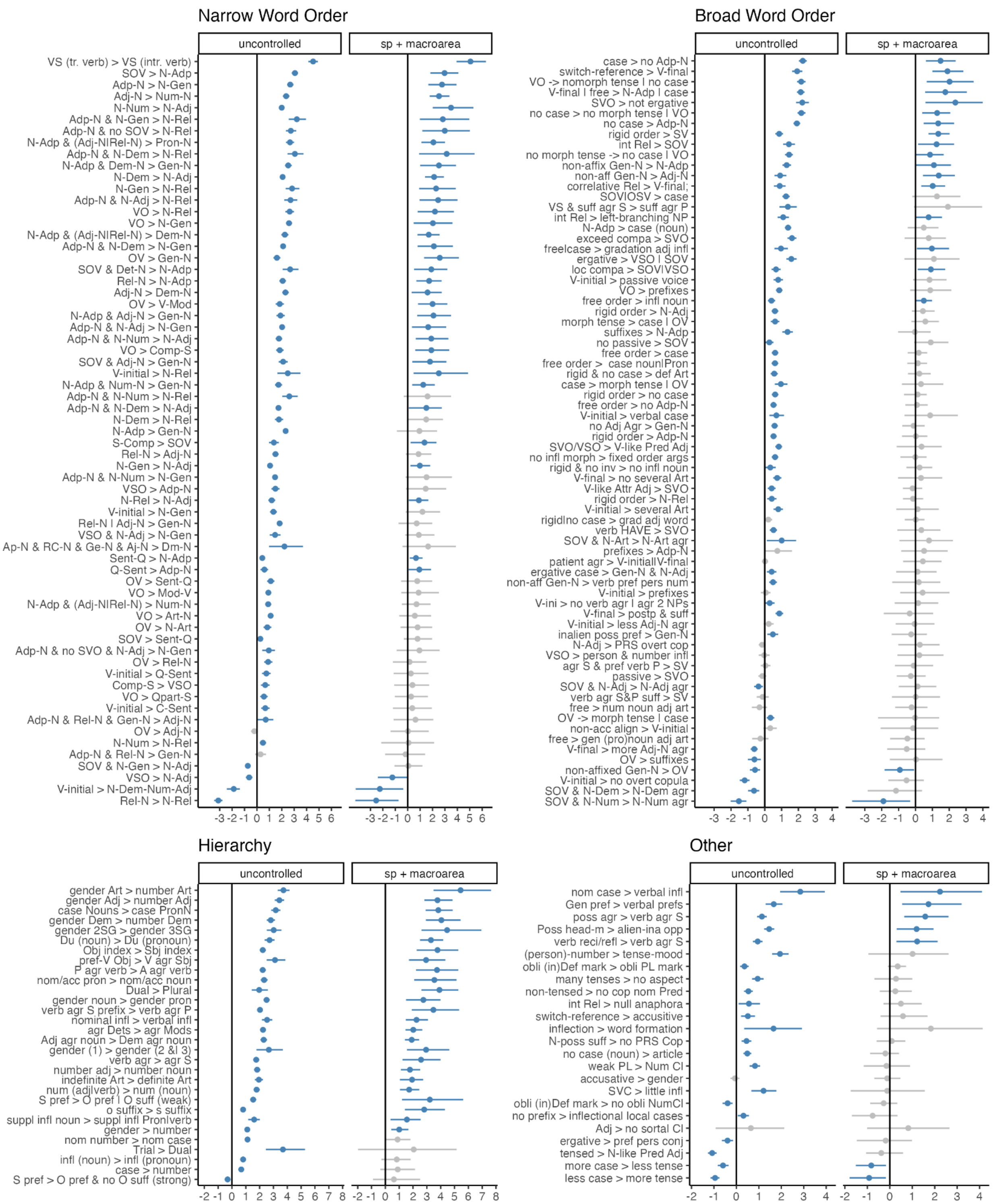

Figure S4. Summary outcomes of the naïve model and the spatiophylogenetic model for 191 implicational statements. Models that do not control for genealogical and geographical relations are on the left, displaying main fixed effect coefficient estimates and their 95% credible intervals. Models controlling for space and phylogeny are on the right, with median estimates of the main fixed effect and their 95% credible intervals. Coefficients from supported universals are colored in blue while those for non-supported universals are colored in gray.

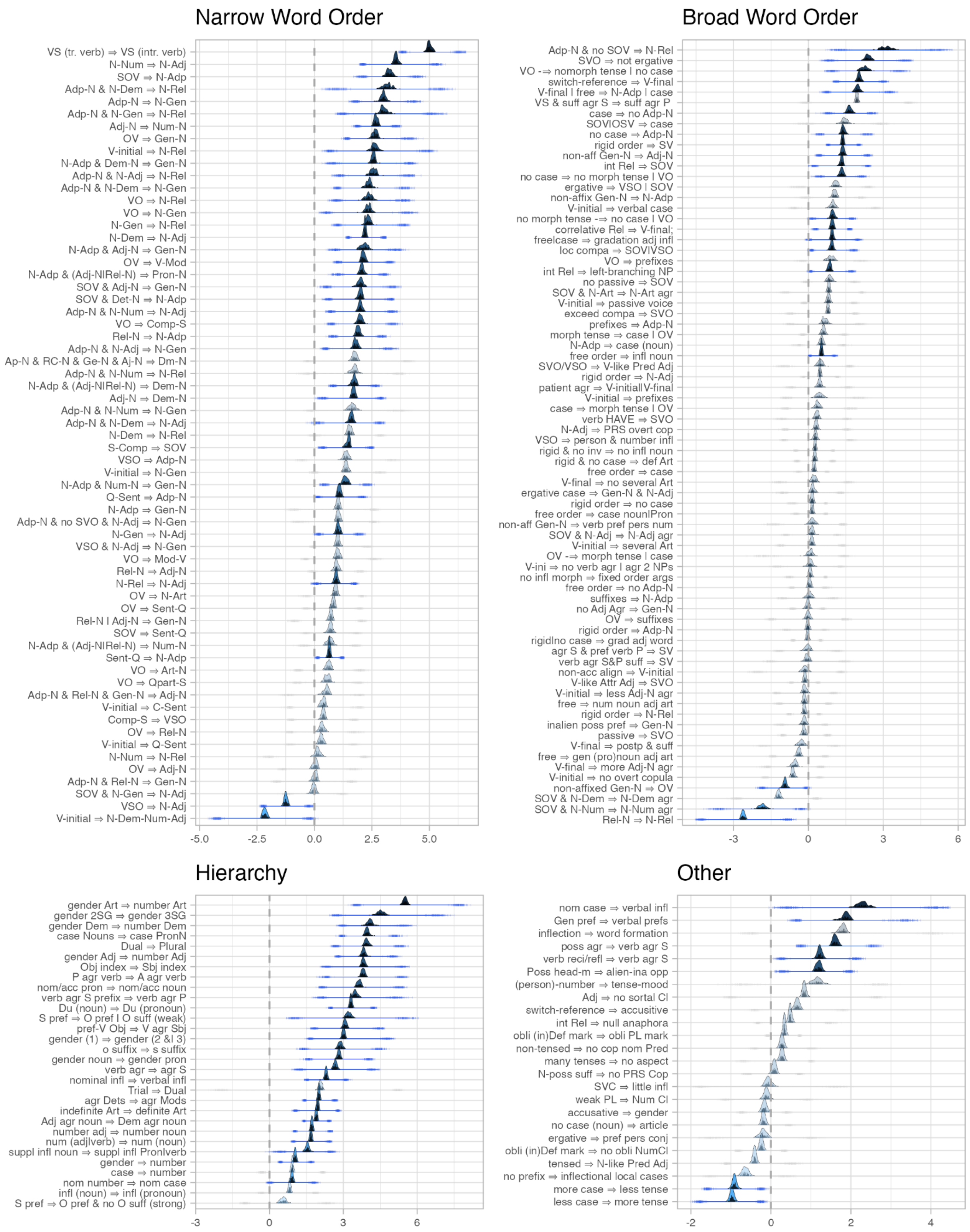

Figure S5: More details on the spatiophylogenetic brms *models over the 100 random posterior trees. Main fixed effect estimates (ridges) and their 95% CIs (error bars) are plotted for each of the 100 analyses. Supported universals (whose median 95% CI does not straddle zero) have 95% CI error bars marked in blue, whereas unsupported universals are drawn with grey error bars and transparent ridgeplots. The colour of the ridgeplots reflects strength of the main fixed effect coefficient. The universals are separated per domain and ordered by median main fixed effect estimate.*

Spatiophylogenetic model vs. language family model
Results from the robustness analysis are included in Figure S6, where we compare our spatiophylogenetic models to an alternative model, which corrects for spatial confounds using a covariance matrix, but with a categorical variable for family relations for genealogical confounds (see Methods and Supplementary Information Text 5). As shown in Figure S6, the results of Bayesian GLMM analyses that use language family as a categorical control for relatedness are qualitatively quite similar to the results obtained by controlling for relatedness using phylogenetic distance calculated on the EDGE phylogeny 100 tree sample. These two sets of results show differences in the support of feature associations for only 25 of the 191 tested universals, as characterized by a 95% credible interval that does/does not include zero. The correlation between the strength of these analyses is high, as reported in the main text (Spearman's r degrees of freedom = 189) = 0.93, $p < 0.001$, 95% Confidence Interval = 0.91 - 0.95).

Three universals that are not supported in models that control for genealogy using phylogenetic distance *do* show supported associations when a categorical family variable is instead used to control for relatedness. One of these is a narrow word order universals involving the relative order of elements in the noun phrase (N-Dem ⇒ N-Rel). The other two universals which are supported only by the GLMMs that implement a categorical control for language family are one hierarchical universal (case ⇒ number) and one 'other' universal ((person)-number ⇒ tense-mood). The relatively small number of universals that are supported by GLMMs with categorical family controls but not those with phylogeny-derived controls for genealogy suggests that the phylogeny we use to represent relationships between languages is not interfering with our ability to detect true universal patterns.

In contrast, twenty-two universals are supported by GLMMs that control for relatedness using a MCC tree from the EDGE phylogeny, but not by those that control for this using a categorical language family variable. Many of these involve word order (twelve narrow word order, five broad word order, two hierarchical, two other universal). Several involve the relative order of adjectives, whose participation in word order universals has previously been questioned on the basis of smaller, sparser samples and categorical representations of genealogy and geography (Dryer 1988, 1992). Our large global sample of languages and use of detailed information about the phylogenetic relationships within language families may enhance our ability to detect universal associations that might otherwise be obscured by lineage-specific and regional trends.

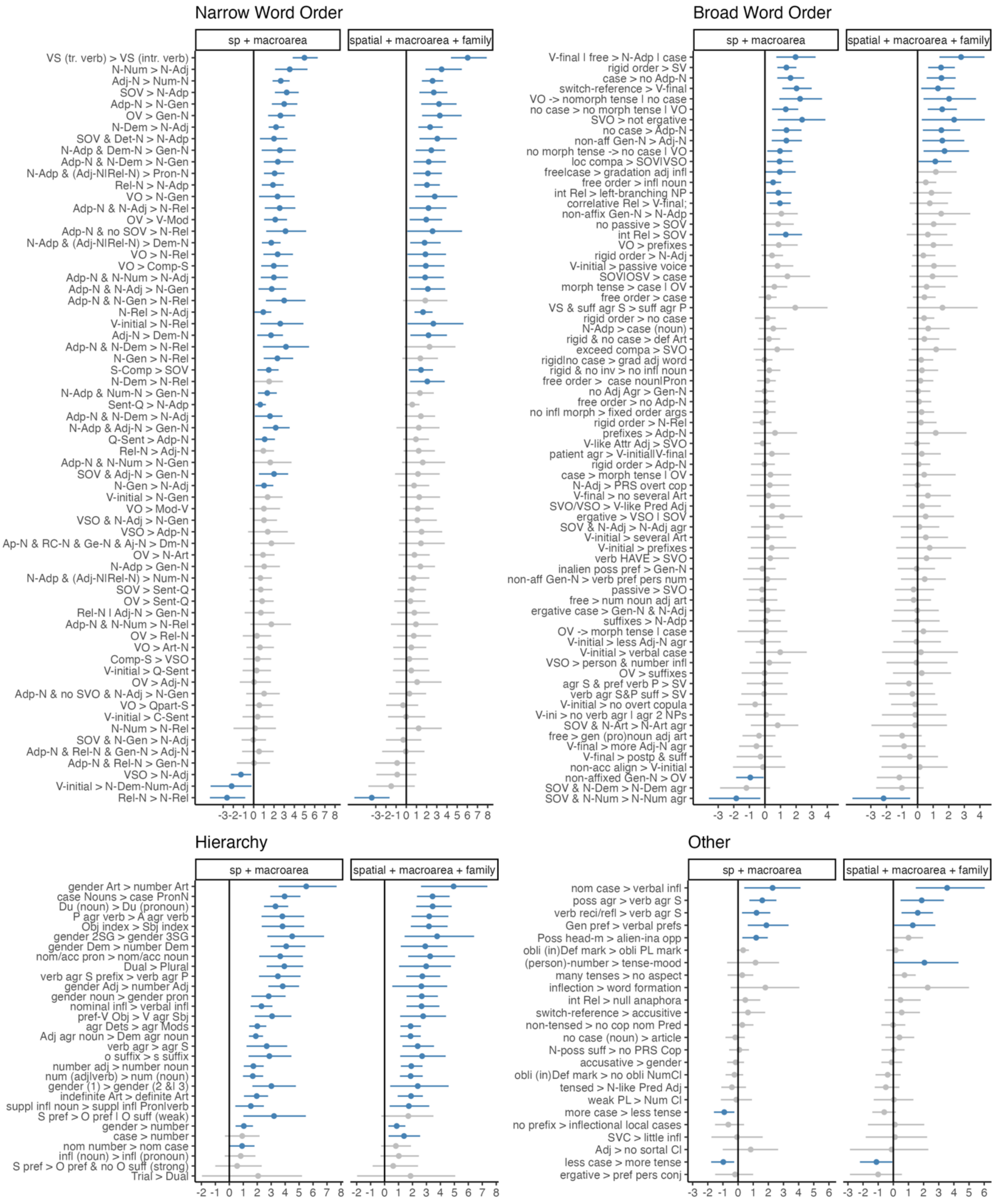

Figure S6. 95% credible intervals of main coefficient estimates from the spatiophylogenetic and language family correlations for 191 implicational statements. Models controlling for space and phylogeny *using matrices are on the left (represented by medians of the main fixed effect estimates and their 95% CIs), while models controlling for genealogical autocorrelation using categorical controls with language families are on the right. Coefficients from supported universals are colored in blue while those for non-supported universals are colored in gray.*

Spatiophylogenetic model vs. coevolution in *BayesTraits*

In the main text, we show that the *brms'* PGLMMs, which correct for spatial and phylogenetic confounds using covariance matrices (see Methods and Supplementary Information Text 5) finds support for 89/191 (47%) of the universals. In Figure 2 in the main text and Figure S7 below, these results are compared to the coevolution analyses performed in *BayesTraits* (see Methods and Supplementary Information Text 5). Taking the intersection of supported universals in the spatiophylogenetic models and the coevolution analyses leaves us with 60/89 (67%) universals that are supported in both analyses, and conversely, 29 universals (33%) that are supported in the spatiophylogenetic models but not in the coevolution analyses.

Figure S7 shows that the effect size of the support for universals across the two analyses is very similar. For example, we find some universals supported in the spatiophylogenetic models that are close to the margin, meaning that either the upper or lower bound of the 95% credible interval of the main coefficient estimates is close to zero. Consequently, the universal is not supported in the *BayesTraits* analyses, as demonstrated by the lower bound of the 95% High Density Interval (95% HDI) on the distribution of Bayes Factors (BF) *not* exceeding 10. An example for that is narrow word order universal 0110KA, "There is a clear tendency for Rel N languages to be Adj N" (Dryer 1988a : 200), with the median 95% CI 0.08 - 1.74 in the spatiophylogenetic model, and a High Density Interval of -6.40 - 14.30 in the coevolution model.

On the other end of the scale, we have some universals that narrowly miss the conservative criterion of the 95% HDI on the distribution of Bayes Factors (BF) exceeding 10. An example of that is "If a language has SOV word order, then if the adjective precedes the noun, the genitive precedes the noun" (Hawkins 1983: 64), whose High Density Interval is 9.51 - 34.34, narrowly missing the cut-off for support.

Other mismatches between the models, where the spatiophylogenetic models show support and the coevolution analyses do not, can be blamed on differences between the two types of analyses. The spatiophylogenetic models include spatial distance as a random effect while the coevolution analyses do not control for spatial autocorrelation at all. In addition, the two types of analyses are conceptually very different (see Supplementary Information Text 5).

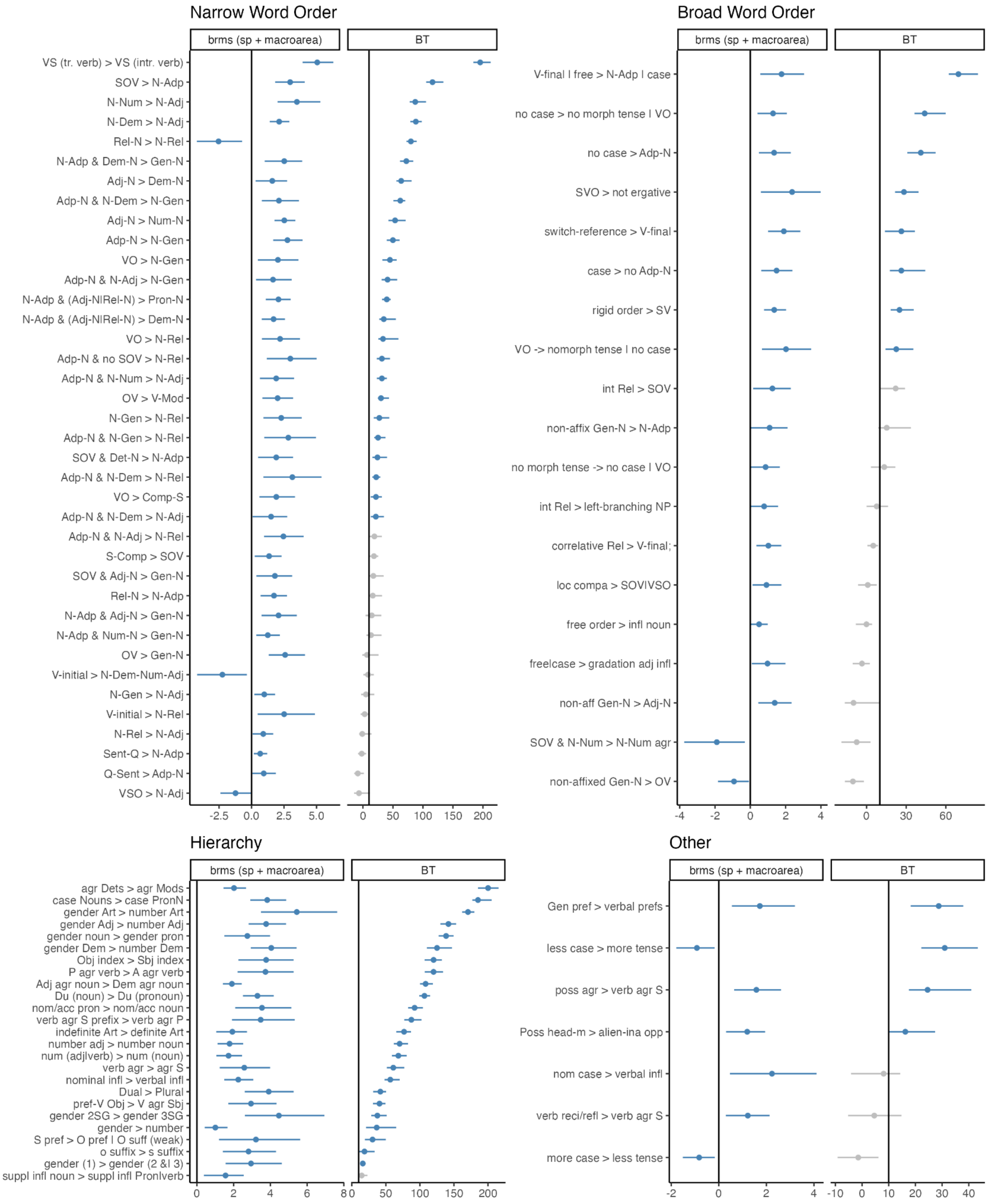

Figure S7. Comparison of the spatiophylogenetic brms *models and the coevolution analyses from* BayesTraits. *On the left, medians of the main fixed effect estimates and their 95% CIs from the spatiophylogenetic models for 89 supported universals. On the right, the 95% High posterior Density Intervals (95% HDI) over the 100 Bayes Factors from the coevolution analyses from* BayesTraits. *Supported universals are colored in blue while non-supported universals are colored in gray. Universals are classified as supported in the spatiophylogenetic analysis if the median 95% CI over the 100 phylogenies excludes zero; for the* BayesTraits *analysis, if the lower bound of 95% HDI exceeds 10.*

Supplementary Information Text 7: Typology of hierarchical universals

As described in the main text, hierarchical universals are those that express dependencies between features in the same morphosyntactic paradigm. In this section, we motivate this category more clearly by listing and discussing each universal and which well-known hierarchy it relates.

The relational hierarchy

This hierarchy was proposed by Croft (1988): "If there is a construction in which the verb agrees with some member of the relational hierarchy Subject > DO > IO > Oblique, then there are at least some constructions in which the verb agrees with members higher on that hierarchy". This was later extended to 'DO > IO > Oblique > [Genitive]'. We investigate a subset of the hierarchy given limitations of the Grambank questionnaire, namely:

1. 0045KA **Obj indexing ⇒ Sbj indexing**

Several other universals that deal with argument marking on the verb are connected conceptually with Croft's (1988) relational hierarchy:

2. 0084KA **verb agreement ⇒ agreement S** - "If in a language the verb agrees with anything, it agrees with some or all subjects" Moravcsik (1974: 27)
3. 0166KA **pref-V Obj ⇒ V Agr Sbj** - "If a verb agrees in prefix with the direct object, then at least some of the verbs agree with the subject" Kozinsky (1981)
4. 0328KA **verb agr S prefix ⇒ verb agr P** - "If a verb agrees with a subject in prefix, then verbs have affixes or clitics which agree with the direct object" Kozinsky (1981)
5. 0331aKA **s prefix ⇒ o prefix & no o suffix** (strong version) - "If the prefix position is the only possible one for a subject-affix, then the object-affix also occupies this position in all forms (with the exception of Wolio) or at least in some of them (no exceptions)" Kozinsky (1981)
6. 0331bKA **s prefix ⇒ o prefix | o suffix** (weak version) - "If the prefix position is the only possible one for a subject-affix, then the object-affix also occupies this position in all forms (with the exception of Wolio) or at least in some of them (no exceptions)" Kozinsky (1981)
7. 0332KA **o suffix ⇒ s suffix** - "If the suffix position is the only possible one for an object-affix, then the subject-affix also occupies this position at least in some cases" Kozinsky (1981)
8. 1611KA **verb-patient agreement ⇒ verb-agent agreement** - "All languages with verb-patient agreement, regardless of type, also have verb agreement with the agent as well" Foster & Hofling (1987: 480)

The definiteness hierarchy

Croft (2003: 132) proposes a definiteness hierarchy (Definite > Specific > Non-specific) based on a scale of accessibility, or in other words how identifiable the referent of a noun phrase is to the hearer. This echoes the definiteness hierarchy (Personal pronoun > Proper noun > Definite NP > Indefinite specific NP > Non-specific NP) introduced by Aissen (2003) to explain differential object marking patterns, where the relationship with the verb is morphologically marked on some objects but not others. We test one universal that reflects the relative ranking of definites and indefinites in both of these formulations of the Definiteness Hierarchy.

9. 1163KA **indefinite article ⇒ definite article** "If a language has a grammaticalized indefinite article, it is likely to also have a definite article, while the reverse does not necessarily hold true." Heine (1997: 69)

The nominal hierarchy

One of the hierarchies in linguistic typology that is most frequently evoked to explain grammatical phenomena is the nominal hierarchy, also called the referentiality hierarchy or the extended animacy hierarchy (Croft 2003). This hierarchy ranks nominals in terms of their semantic, discourse, and morphosyntactic features, where nominal referents that are more salient to speakers or prominent in the discourse, or in other words those that evoke more empathy from speaker and listener, are ranked higher (Haude & Witzlack-Makarevich 2016). This hierarchy, where 1st person pronouns > 2nd person pronouns > Demonstratives and 3rd person pronouns > Proper nouns > Human common nouns > Animate common nouns > Inanimate common nouns, was introduced by research in the 1970s (Silverstein 1976, Dixon 1979). In the intervening decades it has become an indispensable tool for understanding morphosyntactic patterns such as differential argument marking, indexing of arguments on the verb, and alignment of case marking (i.e. how language mark subjects and objects in transitive and intransitive clauses).

Several of the universals we examine involve the referentiality rankings of this hierarchy:

10. 0093KA **gender 2SG ⇒ gender 3SG** - "IF a language has pronominal gender in the 2nd person singular, THEN it has a greater probability of having this distinction in the 3rd person singular than of not having it." Greenberg, Osgood, & Jenkins (1963: xxi)

11. 0183KA **case Nouns ⇒ case PronN** - "If there is case-inflection on nouns, there is also case-inflection on some pronouns." (attributed to Moravscik 1993, but not retrieved)

12. 0320KA **nom/acc pronouns ⇒ nom/acc nouns** - "If personal pronouns have an opposition of the syntactic forms of nominative and accusative, then this opposition is relevant for nouns as well." Kozinsky (1981)

13. 0516KA **Gender Nouns ⇒ Gender ProN** - "If a language has gender categories in the noun, it has gender categories in the pronoun." Greenberg (1963: 96)

14. 0517KA **gender (1) ⇒ gender (2 &| 3)** - "If a language has gender distinctions in the 1st person, it always has gender distinctions in the 2nd or 3rd person, or in both." Greenberg (1963: 96)

15. 0564KA **Du (noun) ⇒ Du (pronoun)** - "If the Dual extends to nouns, it also extends to pronouns." Cuny (1906: 2), Gray (1939: 181), Jensen (1952: 9)

16. 0582KA **inflection (noun) ⇒ inflection (pronoun)** - "If there is any inflection in nouns, there are also some inflections in pronouns." attributed to Moravcsik (1993), data from Hurford & Kirby (1995), but these sources could not be retrieved

The agreement hierarchy

This hierarchy, proposed by Corbett in 1979, describes the likelihood of semantic agreement (and conversely, syntactic agreement), across four targets:

attributive > predicate > relative pronoun > personal pronoun

Where for controllers to the right of the hierarchy, the chance of semantic agreement increases both cross-linguistically as within an individual language. Corbett (2022) uses the word *family* as an example. Subject-verb agreement with *family* can be singular (*My family is* …, this is syntactic agreement) or plural (*My family are* …, this is semantic agreement, reflecting that family refers to a group of individuals). However, this option is not possible for attributive modifiers, i.e. *this family*, **these family*. Several universals relate to the agreement hierarchy, although some make finer-grained distinctions between targets:

17. 0521KA **Adj agree noun ⇒ Dem agree noun** - "In more than one language, and possibly in all, if the adnominal adjective agrees with the noun, so does the adnominal demonstrative" Moravcsik (1997)

18. 1053KA **agreement determiners ⇒ agreement modifiers** - "If determiners agree within NPs, modifiers are likelier also to agree than not to agree" Plank (1994)

We add two universals that relate target and controller. Controllers exist within the same constructions as the targets of agreement involved in Corbett's agreement hierarchy (1979). If gender and number are expressed overtly, expression on the controller itself is relevant to the overall marking patterns that are central to this hierarchy.

19. 0276KA **number (adjective | verb) ⇒ number (noun)** - "If there is a regular number distinction in the forms of the adjective or of the verb, there is the same distinction in the forms of the noun of the given language" Uspensky (1968: 8 [Uspensky 1972: 60]); Vardul (1969)
20. 0237aKA **number Adj ⇒ number noun** - "If adjectives inflect for any category, nouns inflect for this category as well" Smith (1761 [1983]), as interpreted in Plank (1992: 44)

The feature hierarchy

Through his exploration of inflectional morphology, Noyer (1992) proposed a Feature Hierarchy (Person > Number > Gender) to explain which distinctions in inflectional paradigms tend to be neutralized. Harley and Ritter (2002) propose that this hierarchy could be extended to include case, though they do not offer an exact implementation of this extended hierarchy. We include universals that follow the original Feature Hierarchy in this category, as well as universals that include case inflection as either the implicatum or the implicans in combination with number inflection.

21. 0116KA **case ⇒ number** - "number is, as has been seen, closer to the stem and generally present when case is present, while the opposite relation holds far more rarely" Uspensky (1965: 211), inferred from Greenberg (1963: 103)
22. 0513KA **gender ⇒ number** - "If a language has the category of gender, it always has the category of number" Greenberg (1963)
23. 0179aKA **gender Adj ⇒ number Adj** - "There is a hierarchy: Number > Gender > Case, such that if some target agrees in one category on the hierarchy then it will also agree in all categories higher on the hierarchy" (Linfer 1992)
24. 0179bKA **gender Dem ⇒ number Dem** - "There is a hierarchy: Number > Gender > Case, such that if some target agrees in one category on the hierarchy then it will also agree in all categories higher on the hierarchy" (Linfer 1992)
25. 0179cKA **gender Art ⇒ number Art** - "There is a hierarchy: Number > Gender > Case, such that if some target agrees in one category on the hierarchy then it will also agree in all categories higher on the hierarchy" (Linfer 1992)
26. 0229KA **nominal number ⇒ nominal case** - "If nominals inflect for number, they also inflect for case" Campanella (1638), as interpreted in the Universals Archive (Plank and Filimonova 2006).

The number hierarchy

Greenberg's original (1963) proposal concerning universal patterns in grammatical number has led to widespread adoption of a number hierarchy (Corbett 2000: 44): Singular > Plural > Dual > Paucal. As in Greenberg's 1963 formulation, other implementations of this hierarchy (e.g. Miestamo 2009) include trial number (Singular > Plural > Dual > Trial). More recent literature has suggested that the number inflection paradigms that result from this hierarchy may be

partially explained by numerical cognition (Franzon et al. 2019). Two of Greenberg's original universals are central to this hierarchy.

27. 0511aKA **Trial ⇒ Dual** - "No language has a trial number unless it has a dual. No language has a dual unless it has a plural." Greenberg (1963)
28. 0511bKA **Dual ⇒ Plural** - "No language has a trial number unless it has a dual. No language has a dual unless it has a plural." Greenberg (1963)

Then there are two universals left. Although these are not formalized as hierarchies in large bodies of literature, they still rely on relative frequency of related morphological marking patterns and hence we believe their categorization as 'hierarchical' is justified.

29. 0386KA **nominal inflection ⇒ verbal inflection** - "If a language has nominal inflection, it also has verbal inflection" Moravcsik (1994a: 36)
30. 0577KA **suppl noun ⇒ suppl pron|verb** - "If any inflectional category is expressed through suppletion in nouns, it is also expressed suppletively in pronouns or verbs." attributed to Moravcsik (1993) and Sugomoto (1989), but note that these sources could not be retrieved, so it must be a case of mistaken attribution. True source unknown.

Supplementary Information Text 8: Assessment of word order universals

Word order universals have been intensively researched since Greenberg (1963), some examples being Lehmann (1973), Steele (1978), Dryer (1988, 1992, 2009, 2011), Hawkins (1983, 1990, 1994), Dunn et al. (2011), Jäger and Wahle (2021), Hartung et al. (2022); of course, the chapters by Dryer in WALS (Dryer & Haspelmath 2013) contain a wealth of data that has been used by several of these studies. In this section, we present a comparison of our results with four key papers; 1) Dryer (1992), the seminal paper that showed a set of correlation pairs across a sample of 625 languages (Dryer 2009 posits that these claims still hold up with a sample of over 1500 languages), 2) Dunn et al. (2011), who made the case against word order correlations and proposed lineage-specific dependencies, 3) Jäger and Wahle (2021), who tested explicitly whether a universal or a lineage-specific model of word order correlations fit the data better, ad 4) Hartung et al. (2022), who apply a similar approach to Jäger and Wahle (2021) but use logistic Brownian Motion models instead of a continuous time Markov process.

Table S5 presents the results from these three papers and the current study. Dryer (1992) presents no statistical correlations; he proposes correlations between pairs of features if one pairing of feature states is more common than the others in all six continents (Dryer 1989). Here, we indicate support for the correlation as presented by Dryer (1992) with '**sup**' (for 'supported), if the correlation is rejected by Dryer (1992), we mark it with 'not sup'. For correlation pairs that are supposedly also correlated, because they are intercorrelated with the order of verb and object, we note 'sup ind' (for 'supported indirectly'). For Dunn et al. (2011), we summarize over the four language families that they investigated (Austronesian, Bantu, Indo-European, Uto-Aztecan) by stating in how many families the correlation is supported. From Jäger & Wahle (2021), we take BFs from their Table 5, which presents explicit testing against zero-correlation using the Savage-Dickey method. Lastly, for Hartung et al. (2022), we give the number of families for which each trait pair was estimated to be correlated, taken from their Figure 2. In total, the authors took into account 34 language families .

In the final two columns, support for dependent evolution in median BFs from the current phylogenetic coevolution analyses are given. These results are closest in method to Dunn et al. (2011) and Jäger & Wahle (2021), who also employed Bayesian phylogenetics. In some cases, we tested associations between both alternatives of a word order; for the association between the order of adposition and noun and the order of genitive and noun, for example, we test Adp-N ⇒ N-Gen, where both heads are first; and N-Adp ⇒ Gen-N, where both heads are final. These are in effect explicit tests of the bidirectionality of these universals.

Table S5 orders word order correlations by BF as presented in Jäger & Wahle (2021), from highly supported to those having poorest support. Dunn et al. (2011), Jäger & Wahle (2021), and Hartung et al. (2022) test all possible correlation pairs of eight word orders, namely the order of verb and subject (VS), verb and object (VO), adposition and noun (AdpN), noun and genitive (NGen), noun and adjective (NAdj), noun and demonstrative (NDem), noun and numeral (NNum), noun and relative clause (NRel). Since the current study restricts itself to

universals covered in the *Universals Archive*, we do not have results for all of these twenty-eight correlations. All correlations with significant support are marked **in bold**, according to the criterion the respective authors chose, except for Hartung et al. (2022), where support in one or two families is considered as no support, and support in three or more families is considered as support for that correlation.

Jäger & Wahle (2021) present support for thirteen word order universals (the top thirteen rows in Table S5); fifteen other putative universals are not supported by their findings. Of the initial thirteen, Dunn et al. (2011) present support for thirteen family-specific correlations; four are explicitly supported by Dryer (1992), for four more we can assume a correlation given that all involved word orders correlate with the order of object and verb. Hartung et al. (2022) find support for all of the universals in at least one out of 34 tested families, hence their findings are harder to compare to the other studies. In Table S5 we mark support in three or more families as general support for that correlation; if only supported in one or two families, that correlation is not supported. When assessed in this way, Jäger & Wahle's (2021) thirteen supported word order universals are all supported in Hartung et al. (2022), except for VS & AdpN.

Of the fifteen putative universals not supported in Jäger & Wahle (2021), Dunn et al. (2011) present six supported family-specific correlations; none of these are explicitly supported by Dryer (1992), although we may assume a correlation between VS & NRel and NGen & NRel, as all four word orders involved in these are presented by Dryer (1992) as correlated with the order of object and verb. Hartung et al. (2022) report correlations in three or more families for eight out of these fifteen putative universals not supported in Jäger & Wahle (2021); three of them overlap with Dunn et al. (2011). Across analyses, there does seem to be more support for the thirteen word order pairings which Jäger & Wahle (2021) find support for as opposed to the fifteen for which they find no support. However, there are inconsistencies between the analyses as well.

When it comes to the current results from the phylogenetic coevolution analyses, we find support for all eleven universals that we test out of Jäger & Wahle's (2021) well-supported pairings. We also find support for some universals that have not found statistical support elsewhere: NGen & NRel and NDem & NRel (both from Hawkins 1983). Notably, we find differences in how the universal is phrased for three pairings: VO & NGen; VO & NRel; and NAdj & NRel. For VO & NGen, 'VO ⇒ N-Gen' is supported (median BF = 44.99), while 'OV ⇒ Gen-N' is not (BF = 6.68). This is remarkable, because in Dryer (1992), 'OV ⇒ Gen-N' shows much more intra-genus stability than 'VO ⇒ N-Gen'. For VO & NRel, 'VO ⇒ N-Rel' is supported (BF = 33.40), while 'OV ⇒ Rel-N' is not (BF = -0.15). It makes sense that there is no strong support for 'OV ⇒ Rel-N' as prenominal relative clauses are an areal feature of Eurasia and are not strongly represented elsewhere (Dryer 1992). This can be readily observed in the relevant chapters of WALS (Dryer and Haspelmath 2013).

Table S5: Word order correlations across Dryer (1992), Dunn et al. (2011), Jäger & Wahle (2021), Hartung et al. (2022), and the current study

	**Dryer (1992)**	**Dunn et al. (2011)**	**Jäger & Wahle (2021)**	**Hartung et al. (2022)**	**phylo coevo head first**	**phylo coevo head final**
VO & AdpN	**sup**	**sup 2/4**	**19.25**	**5/34**	-	**115.88***
VS & VO	**sup**	**sup 1/4**	**15.82**	**8/34**	-	-
VS & NGen	sup ind	**sup 1/4**	**14.12**	**3/34**	-	-
AdpN & NGen	sup ind	**sup 1/4**	**12.07**	**6/34**	**49.89**	**52.88**
VS & AdpN	sup ind	-	**12.03**	1/34	-	-
NAdj & NNum	-	**sup 1/4**	**10.93**	**7/34**	**87.16**	**53.53**
NDem & NumN	-	**sup 1/4**	**9.98**	**6/34**	-	-
VO & NGen	**sup**	**sup 2/4**	**8.85**	**4/34**	**44.99**	6.68
VO & NRel	**sup**	-	**8.15**	**3/34**	**33.40**	-0.15
NAdj & NRel	-	**sup 2/4**	**7.43**	**3/34**	-1.16	7.09
NAdj & NDem	-	**sup 2/4**	**4.72**	**6/34**	**88.11**	**63.68**
NNum & NRel	-	-	**4.07**	**3/34**	-12.81	-
AdpN & NRel	sup ind	-	**3.83**	**4/34**	-	$16.34^{\$}$
AdpN & NAdj	-	-	3.17	2/34	-	-
NGen & NRel	sup ind	-	2.64	**3/34**	**27.24**	-
VS & NNum	-	-	2.47	2/34	-	-

	**Dryer (1992)**	**Dunn et al. (2011)**	**Jäger & Wahle (2021)**	**Hartung et al. (2022)**	**phylo coevo head first**	**phylo coevo head final**
VO & NNum	-	-	2.01	**4/34**	-	-
VS & NRel	sup ind	-	1.93	1/34	-	-
VO & NAdj	not sup	-	1.60	**3/34**	-	4.74
NDem & NRel	-	-	0.55	**3/34**	**15.89**	-
NGen & NAdj	-	**sup1/4**	-0.03	**4/34**	4.93	-
AdpN & NNum	-	**sup 1/4**	-0.43	2/34	-	-
VS & NAdj	-	**sup 1/4**	-0.51	**3/34**	-	-
NGen & NDem	-	**sup 1/4**	-1.17	**3/34**	-	-
NGen & NNum	-	**sup 1/4**	-1.32	2/34	-	-
AdpN & NDem	-	-	-1.52	2/34	-	-
VO & NDem	not sup	**sup 1/4**	-1.56	2/34	-	-
VS & NDem	-	-	-1.64	**5/34**	-	-
VO & ArtN	**sup**	NA	NA		-0.70	-5.94
SQ & AdpN	sup ind	NA	NA		-8.60	-2.05
VO & CompS	**sup**	NA	NA		**21.61**	-
VO & NegV	not sup	NA	NA		**20.92**	**30.05**

* we investigated Greenberg's (1963) claim "With overwhelmingly greater than chance frequency, languages with normal SOV order are postpositional", 'SOV' is implemented differently than 'OV', see Supplementary Information Text 3.

$ this universal is counted as not supported because the lower bound of its 95% HDI interval (9.19, 31.75) is not > 10.

For NAdj & NRel, neither ‘N-Rel ⇒ N-Adj’ (BF = -1.16) nor ‘Rel-N ⇒ Adj-N’ (BF = 13.59) are supported. This result is probably related to the areality of prenominal relative clauses, coupled with the fact that prenominal adjectives are highly common in Eurasia. This idea is supported by the fact that this correlation is not supported by the corresponding spatiophylogenetic test (95% CI -0.095 - 2.00). When it comes to these thirteen highly supported universals, our results suggest that while some are bidirectional (AdpN & NGen, NAdj & NNum, NAdj & NDem), others are not (VO & NGen; VO & NRel; and NGen & NRel). This is a result that merits further investigation.

The four last rows in Table S5 are devoted to universals investigated by Dryer (1992) and predecessors that we investigate, but Dunn et al. (2011), Jäger & Wahle (2021), and Hartung et al. (2022) do not test. These are:

1. ‘OV ⇒ Art-N’ / ‘VO ⇒ N-Art’ (Dryer 1989);
2. Sent-Q ⇒ N-Adp / Q-Sent ⇒ Adp-N (Greenberg 1963: “With well more than chance frequency, when question particles or affixes are specified in position by reference to the sentence as a whole, if initial, such elements are found in prepositional languages, and, if final, in postpositional”);
3. VO ⇒ CompS (Hawkins 1990: 225: “VO languages are exceptionlessly Comp-initial. OV languages exemplify both final complementizers and initial complementizers”);
4. VO & NegV; taken from Lehmann (1983): “Verbal modifiers like those for negation, causation, and reflexive or reciprocal are placed after verb roots in OV languages and before verb roots in VO languages”. Since Grambank only has information on one such ‘verbal modifier’, namely negation, this universal was implemented as investigating the relation between VO & NegV using Grambank feature GB137: ‘Can standard negation be marked clause-finally?’. Both directions are implemented: OV ⇒ V-Mod and VO ⇒ Mod-V.

Our results for these four go directly against Dryer’s (1992) findings, with the exception of VO ⇒ CompS, which is supported in our analysis. Since the word orders involved are syntactically more complex than the eight investigated by Dunn et al. (2011) and Jäger and Wahle (2021), we believe that the reason for this might be related to what the original proposals and Dryer (1992) define as ‘article’, ‘question particle’, and ‘negator’, and how these concepts are implemented in Grambank. We refer to Supplementary Information Text 9, SI Data 1, the overview of the Grambank questionnaire (Table S1 in Supplementary Information Text 1), as well as further information on the Grambank questionnaire in Skirgård et al. (2023).

Supplementary Information Text 9: Explaining support for universals

In this section, we explore the support for the universals tested in more detail, especially linking our results to previous literature. We start by exploring the universals in terms of their historical presentation, namely the language sample on the basis of which they were first proposed and whether that sample was regionally biased, as well as the fit of the original formulation to its implementation in the current study (see Supplementary Information Text 3). As it turns out, none of these seem to impact the support of a given universal. What does explain support for universals is their *type* (see main text and Figure S8 below). Therefore we discuss possible functional explanations for different types of universals in the last part of this section. Supported universals are those that were supported in the spatiophylogenetic *brms* models (median 95% CI excludes zero) and the *BayesTraits* coevolution analyses (lower bound of the 95% HDI on Bayes Factors > 10).

Sample size, bias, and implementation.

In order to explain our findings, we were interested in the conditions under which the universals were first proposed. Many of the strongest supported universals were formulated in the 1960's and 1970's, and the language samples then were often quite small and biased due to lack of language documentation. For example, Greenberg's (1963) famous set of linguistic universals was proposed on the basis of a sample of only thirty languages. Greenberg's sample had clear bias towards Eurasia: fifteen out of thirty languages are Eurasian, and neither a North American nor a New Guinea language was included, in spite of the great linguistic diversity of these two regions. In addition, there is some variability in how well we were able to capture the original wording of the universals given the Grambank questionnaire. Hence, we collected data on sample size, geographical bias, and implementation, this data is included in SI Data 1.

First, we examine language sample size and bias. The universals investigated in this study have been proposed either on the basis of very few languages (in some cases, a pattern observed in one or a few languages is proposed to be universal on the basis of theoretical considerations), or on large samples (350, 389, 410 or 500 languages), and on everything in between. As illustrated in Figure S1, smaller samples are more common. Taking individual sources (the 191 investigated universals stem from sixty unique publications), mean sample size is 86.6 and median sample size is 39. Many of these samples have a regional bias, in particular bias *against* the Pacific, New Guinea, Australia, and/or South America or bias *towards* Eurasia. Some studies exclusively sample one family or area explicitly. In Figure S8 below, these are collapsed under 'regional bias'. Regional biases were common due to restrictions on the documentation of the languages of the world as well as the availability of descriptions. These restrictions, especially the first one (bias *against* the Pacific, New Guinea, Australia, and/or South America), still play a major role

today as these are some of the most diverse and yet under-documented regions linguistically (Hammarström et al. 2018).

Second, when considering how well we were able to implement the universal in terms of Grambank questionnaire questions and combinations thereof, we considered three levels: 1) perfect, 2) OK, and 3) less than OK. The latter two have been merged in Figure S8. Examples of each are:

1. **Perfect**: Hawkins (1983): "If a language has Prep word order, then if the adjective follows the noun, the genitive follows the noun." is implemented as *Adp-N & N-Adj ⇒ N-Gen*, which captures the original wording perfectly.
2. **OK**: Greenberg (1963): "Universal 5. If a language has dominant SOV order and the genitive follows the governing noun, then the adjective likewise follows the noun." is implemented as *'SOV'/'verb-final' & N-Gen ⇒ N-Adj*. We cannot capture SOV word order as the relevant Grambank questions (GB131, GB132, GB133) ask only about the position of the verb (verb-final hence implies SOV and the uncommon OSV order). Nor can we capture 'dominance', which implies that SOV word order is supposed to be the most common one. The Grambank questionnaire asks about pragmatically unmarked constituent orders and does not capture which word order is most common.
3. **Less than OK**: Klimov (1973): "In ergative languages personal conjugation is of prefixal or prefix-suffixal nature" is implemented as *ergative ⇒ prefixal personal conjugation*. The problem with implementing this universal is that its latter part describes two types, "prefixal nature" and "prefix-suffixal nature". However, if we included languages with suffixes, basically almost all languages would be coded as "yes" for "prefixal or prefix-suffixal nature". Hence, we focus exclusively on the presence of prefixes, which means that we are probably testing a too strict version of the universal.

In order to assess whether any of the variables (sample size, bias, implementation) explained whether the universals were supported in the spatiophylogenetic *brms* models and the *BayesTraits* coevolution analysis or not, we conducted a logistic regression in R (R Core Team 2023). The response variable is binary, there is either support in both the *brms* and *BayesTraits* analysis or neither. This subsets the dataset from 191 universals to 162 as those which are supported in the *brms* analysis but not in *BayesTraits* are excluded. Sample size, bias, and implementation (as described above, i.e. binned) are the predictors, the model is a Generalised Linear Model with a binomial response family distribution. The results are presented in Table S6 below. None of the variables affect support significantly.

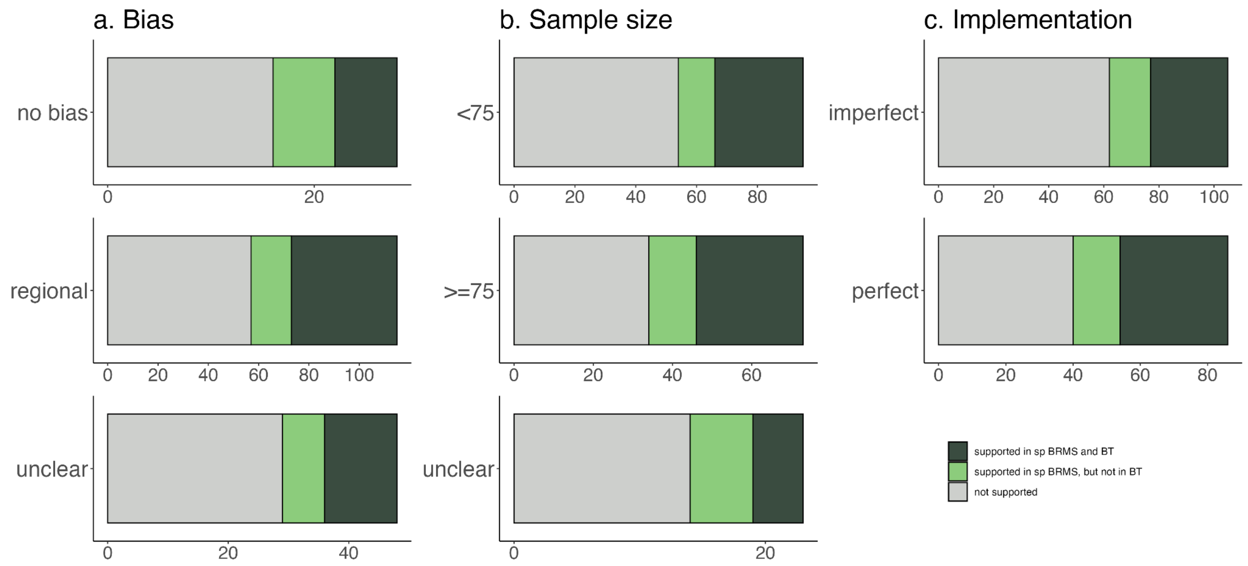

Figure S8. Proportion of universals that were supported in the spatiophylogenetic brms *models and the* BayesTraits (BT) *coevolution analysis (n = 191). Support in spatiophylogenetic brms models is operationalised such that the median of 95% CI should exclude zero; for the* BT *analyses, the 95% HDI of Bayes' Factors should > 10. Grey = no support in* brms *or* BT*, light green = support in spatiophylogenetic* brms only *and dark green = support in spatiophylogenetic* brms *and* BT *analysis. The results are visualised stratified by sample size, bias and assessment of the implementation of the universal in terms of Grambank features. Universals that were not supported in the spatiophylogenetic* brms *models were not analyzed in* BayesTraits*.*

The majority of the investigated universals were suggested based on a sample with regional bias (64%), given that in 18 out of 60 sources, samples are either unclear or too small to be considered biased or unbiased (see SI Data 1). However, as Figure S8a shows, we do not find that universals proposed on the basis of an unbiased sample are better supported in our global analysis. Turning to sample size, Figure S8b shows how many supported and unsupported universals are found depending on whether the sample used to first propose the universal is smaller than 75 language (top bars) or equal to or bigger than 75 language (middle bars). Universals first proposed on the basis of larger samples do slightly better in our global analysis, but the difference is not large (and not significant). This shows that limited representation of world-wide linguistic diversity in 20th century research on universals did not prevent the discovery of universals or development of sound linguistic theory.

We do find that universals that can be implemented in Grambank features better have a larger proportion of support (Figure S8c). The difference is however not large, not significant, and we can conclude that neither original sample size, original sampling bias, and our (admittedly, self-assessed) implementation score do not play a large role in explaining which universals are

supported and which are not. What does matter, however, is the type of the universal (broad word order, narrow word order, hierarchy or other); explanations for different types of universals are discussed in the remainder of this section.

Table S6: Results for modeling support in terms of sample size, bias, and implementation

	Estimate	Std. Error	z value	Pr(>|z|)
(Intercept)	1.35	0.54	2.5	0.01
Sample size: >=75	-0.45	0.36	-1.25	0.21
Sample size: unclear	0.51	0.75	0.68	0.49
Bias: regional	-0.62	0.55	-1.12	0.26
Bias: unclear	-0.4	0.66	-0.6	0.55
Implementation: perfect	-0.45	0.35	-1.27	0.2

Null deviance: 213.57 on 161 degrees of freedom. Residual deviance: 205.98 on 156 degrees of freedom, AIC: 217.98

Narrow word order universals.
Narrow word order universals are those that relate two elements that both concern word order, for example:

- When the descriptive adjective precedes the noun, the demonstrative and the numeral, with overwhelmingly more than chance frequency, do likewise. (Greenberg 1963)
  - operationalised with Grambank features into two separate tests:
    - if GB193 is 1 (yes), then GB025 is 1 (yes)
    - if GB193 is 1 (yes), then GB024 is 1 (yes)
- There is a clear tendency for Rel N languages to be Adj N and for N Rel languages to be N Adj. (Dryer 1988: 200)
  - operationalised with Grambank features into:
    - if GB327 is 1 (yes), then GB193 is 2 (postposed)

In our study, this category contains only universals where both elements concern word order. Universals where one element relates to word order, and the other does not, are instead categorised as broad word order universals. An example is “In verb-initial languages, the existence of several articles (definite, indefinite, specific, plural, proper noun) is much more common than in verb-final languages”; unpublished statements of Keenan, reproduced in Payne 1990: 13). Broad word order universals are discussed later on in this section, here we focus on narrow word order universals.

This group of typological universals has been widely investigated since the foundation of the field of linguistic typology (i.a. Culbertson 2017; Cysouw 2010; Dryer 1988, 1992, 2009, 2018, 2019; Dunn et al. 2011; Futrell, Levy, and Gibson 2020; Gerdes, Kahane, and Chen 2019; Greenberg 1963; Hahn, Jurafsky, and Futrell 2020; Hahn & Xu 2022; Hartung et al. 2022; Hawkins 1983, 1990, 1994, 2014; Lehmann 1973; Jäger & Wahle 2021; Jing, Widmer and Bickel 2011; Payne 1990; Steele 1978). Several different types of explanations for them have been put forward; see the introduction to Good 2008 for a classification into structural, historical, and external types of classifications, which also emerges from the following.

Firstly, what we categorise as narrow word order universals have been at the heart of syntactic theory regarding phrase structure relations and how they interact (Baker 2001; Biberauer & Roberts 2005; contributions in Biberauer and Sheehan 2013; Cinque 2005, 2013, 2014; Kayne 1994; Sheehan et al. 2017; contributions in Svenonius 2000, Svenonius 2025, and many more). For example, Biberauer and Roberts (2005) explain word order correlations in terms of overarching parameter settings; while Sheehan et al. (2017) discuss the Final-over-Final condition, a strong cross-linguistic constraint on headedness within head-final phrases. See Whitman (2008) for a proposal connecting formal accounts to diachronic change.

Secondly, narrow word order universals have also played a central role in functional accounts of language which are rooted in interactional pragmatics, cognitive processing, and language learning. Since the dawn of functional linguistic typology with Greenberg (1963), the field has studied many different word order relationships and found striking patterns in the world's languages. Table S6 illustrates the differences in word order patterns in English and Japanese. The patterns in these different phrases are to a large extent intertwined and illustrate larger trends in languages the world over. The pattern illustrated here, like many narrow word order universals, can be conceptualized in terms of "heads" and "dependents". Simplistically, we can define heads as the most important part of a phrase (*women* in the phrase *wonderful women who whistle*). The head determines the word class of the phrase and it is usually impossible to leave it out. You can say *women* in place of *wonderful women who whistle,* but not **wonderful who whistle.* Dependents can, in a simplified manner, be defined negatively – they are not heads. Dependents can complement the head, which means they cannot be left out and have to be of a certain word class (such as *the table* in the phrase *on the table).* Dependents can alternatively modify the head, as in *wonderful* and *who whistle* in the phrase *wonderful women who whistle*. Dependents of the second type can be left out without rendering the phrase ungrammatical. To summarize these facts about optionality and required word classes, we can say that dependents *depend* on their heads, whereas the reverse is not true. These two types of elements in a phrase, heads and dependents, show cross-linguistic consistent patterns in their ordering.

The heads of phrases (the verb in a clause, the noun in a noun phrase, etc.) have been marked out in bold in Table S6. In English, the heads in each phrase appear first with its dependents (objects, genitive, relative clause, etc.) following. However in Japanese the pattern is the opposite: the dependents appear before the head. The order of verb and object in a given language can often predict the order of adposition (e.g. prepositions, postpositions) and nouns as well as the order of nouns and their relative clauses (Dryer 1992). It is less common for a language to have a mix, with some phrases having heads first and others dependents first. This is one of the patterns that was noticed early in linguistic typology and which falls into the category of narrow word order universals.

Table S6: English and Japanese phrases that illustrate different possible orders of heads and dependents. Taken in part from Dryer (1992: 108).

**1.**	**ate** the sandwich<br>**VERB** OBJECT	サンドイッチを食べた<br>*sandoitchi o* ***tabeta***<br>sandwich OBJ eat.PST<br>OBJECT **VERB**
2.	the book is [**on** the table]<br>**ADPOSITION** NOUN	本が [テーブルの上に] ある<br>*hon ga [te:buru no ue* ***ni****] aru*<br>book SBJ table GEN top on be.PST<br>NOUN **ADPOSITION**
**3.**	**king** of the people<br>**NOUN** GENITIVE	人民の王様<br>*jinmin no* ***o:sama***<br>people GEN king<br>GENITIVE **NOUN**
**4.**	**movies** that we saw<br>NOUN RELATIVE CLAUSE	私達が見た映画<br>*watashitachi ga mita* ***eiga***<br>1PL SBJ see.PST movie<br>RELATIVE CLAUSE **NOUN**
**5.**	**ran** slowly<br>**VERB** MANNER ADVERB	ゆっくり走った<br>*yukkuri* ***hashitta***<br>slowly run.PST<br>MANNER ADVERB **VERB**

Why, then, do we find these striking patterns in cross-linguistic studies? Much of the academic debate has concerned three key facets of this conundrum: (1) exactly which constituent orders enter in such correlations, (2) whether the patterns are rooted in head-dependent structure or in something else, and (3) how to explain these patterns. Our study directly addresses only point (1), but nevertheless we aim to give an overview of point (3) and a discussion of how such explanations speak to the narrow word order correlations we find evidence for.

As for the first of these questions, Dryer (1992, 2009), for example, demonstrated that word orders exemplified in Table S6 above (the order of adposition and noun; noun and genitive, noun and relative clause, and verb and manner adverb) correlate with the word order of object and verb (example 1 in Table S6), but the order of noun and adjective does not (Dryer 1988). Recent quantitative investigations discussed below have further refined the set of constituent orders involved in narrow word order correlations.

Regarding (2), there exists a considerable amount of theoretical work discussing whether these patterns should be understood in terms of the previously defined head-dependent framework, or if there is another approach to the patterns. Hawkins (1983) explains these correlations using slightly different terms: *operator* (similar to heads of phrases) and *operand* (similar to modifiers); languages prefer these to be harmonic (have the same order) across categories (different types of phrases). His ideas are rooted in the works of Lehmann (1973) and Vennemann (1974), who – similar to Dryer – take the order of object and verb in a clause as the central pattern, with the word order within other phrases following from it. Dryer (1992) argues against taking head-dependent structure as the basis for these word order correlations. Instead, he shifts the focus away from dependency relations as the primary explanation and argues instead that languages have a broader, more consistent tendency toward left- or right-branching structures (placing elements consistently before or after the element that determines the category of a phrase). Within this framework, we would say that phrasal categories precede non-phrasal categories (e.g. individual words like verbs or adpositions) in VO languages like English, and vice versa for OV languages such as Japanese.

Lastly, we arrive at our third and perhaps most important point: explanations for narrow word order universals. These have been rooted in ideas about efficient processing since Keenan & Comrie (1977), Corbett (1979), Dryer (1992) and Hawkins (1994, 2004). Hawkins' (1994) principle of Early Immediate Constituents correctly predicts the internal ordering of words within phrases and the placement of phrases within the sentence. It is based on the idea that dependents are placed as closely as possible to their heads (which he calls phrasal mother nodes). This idea has given rise to a huge quantitative literature that investigates what has come to be known as *dependency locality* (see Futrell, Levy, & Gibson 2020 for an overview), which posits that short dependency lengths between heads and their dependents (measured by number of intervening words, morphemes or nodes) are preferred. We can illustrate this idea by taking two examples from Hawkins (1994: 65):

(a) "That Bill was frightened surprised Mary"
(b) "It surprised Mary that Bill was frightened"

(b) is preferred to (a), as (a) has a long dependency relation between *that* and *surprised*. This distance is reduced in (b) by the use of a so-called "cleft" structure. Futrell, Levy, & Gibson (2020), amongst many others, describe how this principle holds across languages, and how short dependencies impact efficiency by reducing working memory load.

Dependency locality is involved with word order correlations because consistent head-dependent direction across constituent phrases shortens dependencies. Below is the same sentence in English (c) and Japanese (d), which illustrate this:

(c)

<table>
<tr><td>English</td><td>The young child</td><td>pushed</td><td>the three blocks</td><td>into the container</td><td>one by one</td></tr>
<tr><td rowspan="2">Grammatical roles</td><td rowspan="2">Subject</td><td>Head of predicate</td><td>Direct object</td><td>Oblique object</td><td>Manner adverbial</td></tr>
<tr><td colspan="4">Predicate</td></tr>
</table>

(d)

<table>
<tr><td>Japanese script</td><td>幼い子供は</td><td>三個のブロックを</td><td>一個ずつ</td><td>容器の中に</td><td>押し込んだ</td></tr>
<tr><td>Latin transliteration</td><td>Osanai kodomo wa</td><td>san-ko no burokku o</td><td>ichi-ko zutsu</td><td>yōki no naka ni</td><td>oshikomima shita</td></tr>
<tr><td rowspan="2">Grammatical roles</td><td rowspan="2">Subject</td><td>Direct object</td><td>Manner adverbial</td><td>Oblique object</td><td>Head of predicate</td></tr>
<tr><td colspan="4">Predicate</td></tr>
<tr><td>English translation</td><td>The young child</td><td>three blocks</td><td>one by one</td><td>into the container</td><td>pushed</td></tr>
</table>

In example (c) and (d), the phrases are delimited by columns and marked for their grammatical role. Heads are marked in bold. In English, sentence (c) illustrates some of the same word orders in Table S6: the verb precedes both objects and the manner adverbial; the adposition precedes the noun. Other heads (*child*, *blocks*) are preceded by dependents, such as the adjective *young* and the numeral *three*. In Japanese, sentence (d) illustrates that several head-dependent orders are inverted. The verb 押し込んだ (oshikomimashita) is placed at the end of the clause with all of the objects and the manner adverbial preceding it. 中 (naka) is a postposition and comes after the noun 容器 'container'. Now just consider the dependency between the verb and the oblique

object (容器の中に yōki no naka ni), headed by the adposition 中 (naka). Imagine that in English, *into* is not a preposition but a postposition: ‘the container into’. The dependency length between the verb ('pushed') and the head of the oblique object just increased. The same applies to Japanese: the postposition 中 (naka) ‘inside’ is closer to its verbal head than if it were to precede its complement noun. If the word order is head-initial for some constituents and head-final for some, the total length of dependencies increases. If the language is instead consistently head-initial or head-final, the dependencies are shorter. This applies to other dependency relations as well, and therefore the proposal has been that word order correlations are rooted in dependency locality, and dependency locality is rooted in a need for efficient processing. Processing principles likewise explain the optimal placement of heavy constituents (Hawkins 1994) and, in part, ordering of modifiers within the noun phrase (Dryer 2018).

Another explanation for the strength of many narrow word order universals is rooted in learnability constraints. Baker (2001) described the *head directionality parameter*, which posits that a single parameter is set (to either ‘head-initial’ or ‘head-final’) that then determines the orders of a range of constituents across different phrase types, such as those in Table S6 above. This makes the entire system easier to learn. Culbertson et al. (2020a) show that even speakers of languages in which the word order of adjective and noun does not align with that of numeral and noun (hence is disharmonic), prefer for these two word orders to align in artificial language learning experiments (see also Martin et al. 2024).

A last type of explanation relates to grammaticalization and other more general pathways of diachronic change (i.a. Bybee 1988; Lehmann 1992; LaPolla 2002, Kuteva and Heine 2008). Grammaticalization is the process by which lexical elements, such as nouns or verbs, over time become semantically bleached, phonologically reduced and more obligatory – that is, they become part of grammar. Explanations of this type draw on specific patterns of collocations in production and likely change over time to describe larger patterns. Aristar (1991) for example, accounts for the strength of the specific narrow word order universals involving the adposition and the noun and the noun and its modifiers in such a way. Dryer (2019) and Collins (2019) give a recent overview of such explanations, showing that the association between the order of object and verb and the order of adposition and noun can been explained through the common process of grammaticalization where adpositions develop from verbs. In such an account, dative adpositions like *for* in a clause like *Carmen made the pie for Anna* may arise from verbs meaning ‘give’. If the word order is such that verbs come before their objects, then the grammaticalization process will give rise to prepositions rather than postpositions (*for Anna* rather than *Anna for*). Marchello-Nizia (2009) also emphasizes particular diachronic pathways and system analogy to explain the transition from head-final order in Latin to head-initial in French. Importantly, such ‘source-oriented’ explanations (Cristofaro 2014, 2017, 2019; Harris 2008, Mansfield and Krapp 2025) imply that explaining word orders is not necessarily rooted in cognitive explanations (e.g.

ease of processing or learnability constraints), but rather falls out of collocations and common diachronic patterns of change (e.g. *give -> for*).

There is as of yet relatively little work that attempts to pull apart different explanations for different word order correlations, or determine their relative strength (see Nichols 2008 for a tentative 'consensus' and Tily and Jaeger 2011 for a proposal on methodology). Our study likewise cannot differentiate which of the previously discussed explanations is more likely to explain the support of the tested universals. Our contribution relates to the strength of the patterns themselves – which universals stand strong when rigorous controls for shared descent and contact are implemented? Our attempts to point out the most appropriate explanations are therefore speculative. We first give an overview of which types of narrow word order universals are indeed significant in our study, and then return to explanations once more.

Bearing in mind that the Grambank implementation of universals does not specifically target the order of object and verb (see Supplementary Information Text 3), our results do not generally support the relationship between the order of object and verb and other clausal elements, such as the order of complementizers (GB421 & GB422) and question markers (GB262 & GB263) relative to the clause. There are two exceptions to this general trend, two universals that relate to verb order and other clausal orders: OV > V-Mod (Lehmann 1973) and VO > Comp-S (Hawkins 1990). A third exception is the strongest supported narrow word order universal, VS (tr. verb) > VS (intr. verb) (Kozinsky 1981). This association is a bit different to the other clausal word order universals as it pertains to a relationship across two clause types (transitive & intransitive) where the relevant elements occur in the same order in both clause types. The same applies to Rel-N > N-Rel ("For any language, preposed relative clause implies postposed relative clause", Moravcsik 1969: 81). This universal actually has negative *brms* coefficients, so the correlation is actually between Rel-N > NOT N-Rel; languages with preposed relative clauses typically do *not* have postposed relative clauses. Our results also do not support the relationship between the order of object and verb and adnominal word orders, such as that of noun and adjective. There are two exceptions, both from Greenberg 1963: VO > N-Rel and VO > N-Gen. However, these are discussed in light of the support for other universals below.

When it comes to the order of adposition and noun, these do not show associations with clausal word orders, such as Greenberg's (1963) universal Q-Sent > Adp-N. Postpositions (N-Adp order) do have a relationship with object-verb order, as attested in two similar universals that are both supported: SOV & Det-N > N-Adp (Hagége 1982) and SOV > N-Adp (Greenberg 1963). However, most universals we tested in relation to the order of adposition and noun deal with adnominal word orders. Of these, about half are not supported and about half are. We also consider predictions regarding the order of various adnominal modifiers and the noun here, as the pattern that emerges there is similar to those involving the order of adposition and noun and adnominal word orders.

In languages with prepositions, we find support for certain modifiers to be placed after the head noun. The strongest of these is Greenberg's (1963) Adp-N > N-Gen. Others are N-Adj, N-Rel, N-Dem and N-Num. These are also supported independently from the order of adposition and noun (N-Dem > N-Adj; N-Gen > N-Rel; N-Num > N-Adj, all from Hawkins 1983). There are also universals that relate the order of adposition and noun with the order of noun and various modifiers that do **not** obtain support in our study, most of these involve the order of noun and numeral and noun and relative clause. It seems that there are two bundles of correlated adnominal word orders: (1) prepositions tend to occur with N-Dem, N-Gen and N-Rel order; languages with prepositions also tend to be N-Num and N-Adj. These do not (always) intersect, as there is no support for example for Adp-N & N-Adj > N-Rel; Adp-N & N-Num > N-Gen; or Adp-N & N-Num > N-Rel (all Hawkins 1983), nor is there for N-Dem > N-Rel, N-Gen > N-Adj, N-Num > N-Rel (all Hawkins 1983), or N-Rel > N-Adj (Dryer 1988).

For languages with postpositions, we would expect the mirror image of what we find for prepositional languages: postpositions corresponding with pre-nominal modifiers. However, there is less support for these associations. There is no support for N-Adp > Gen-N (Greenberg 1963), while N-Adp & Dem-N > Gen-N (Hawkins 1983) **is** supported. In postpositional languages, the only two supported prenominal modifiers are demonstratives (N-Adp & (Adj-N|Rel-N) > Dem-N; Hawkins 1983) and possessive pronouns (N-Adp & (Adj-N|Rel-N) > Pron-N; Hawkins 1983). There is no evidence that postpositional languages tend to have Num-N or Adj-N order. In addition, when we look at word order universals with prenominal modifiers, we find that correlations involving Rel-N do not hold (Rel-N > Adj-N, Rel-N > N-Adp (Greenberg 1963), Rel-N | Adj-N > Gen-N (Givón 1971)). The only supported universals involving prenominal modifiers are Adj-N > Dem-N and Adj-N > Num-N (Hawkins 1983).

In summary, our results support several hubs of correlated word orders, illustrated in Figure S9:

1. Prepositional languages tend to have postnominal modifiers, but not necessary all of them; there is an interconnected hub of N-Dem, N-Gen and N-Rel order; while another major hub involves N-Num and N-Adj;
2. Postpositional languages do **not** tend to have prenominal modifiers in general, only prenominal demonstratives and possessive pronouns. When it comes to prenominal modifiers in general, there exists an association between Adj-N, Dem-N, Num-N.
3. Regarding the relationship between the order of object and verb and other word orders, we find the expected association for the order of adposition and noun; but only for verb-final languages (SOV & Det-N > N-Adp, SOV > N-Adp) and not for verb-initial ones (VSO > Adp-N, Greenberg 1963). While we do not test VO > Adp-N directly, it could explain the support for VO > N-Rel and VO > N-Gen.
4. The only two clausal narrow word order universals that are supported are OV > V-Mod (Lehmann 1973) and VO > Comp-S (Hawkins 1990).

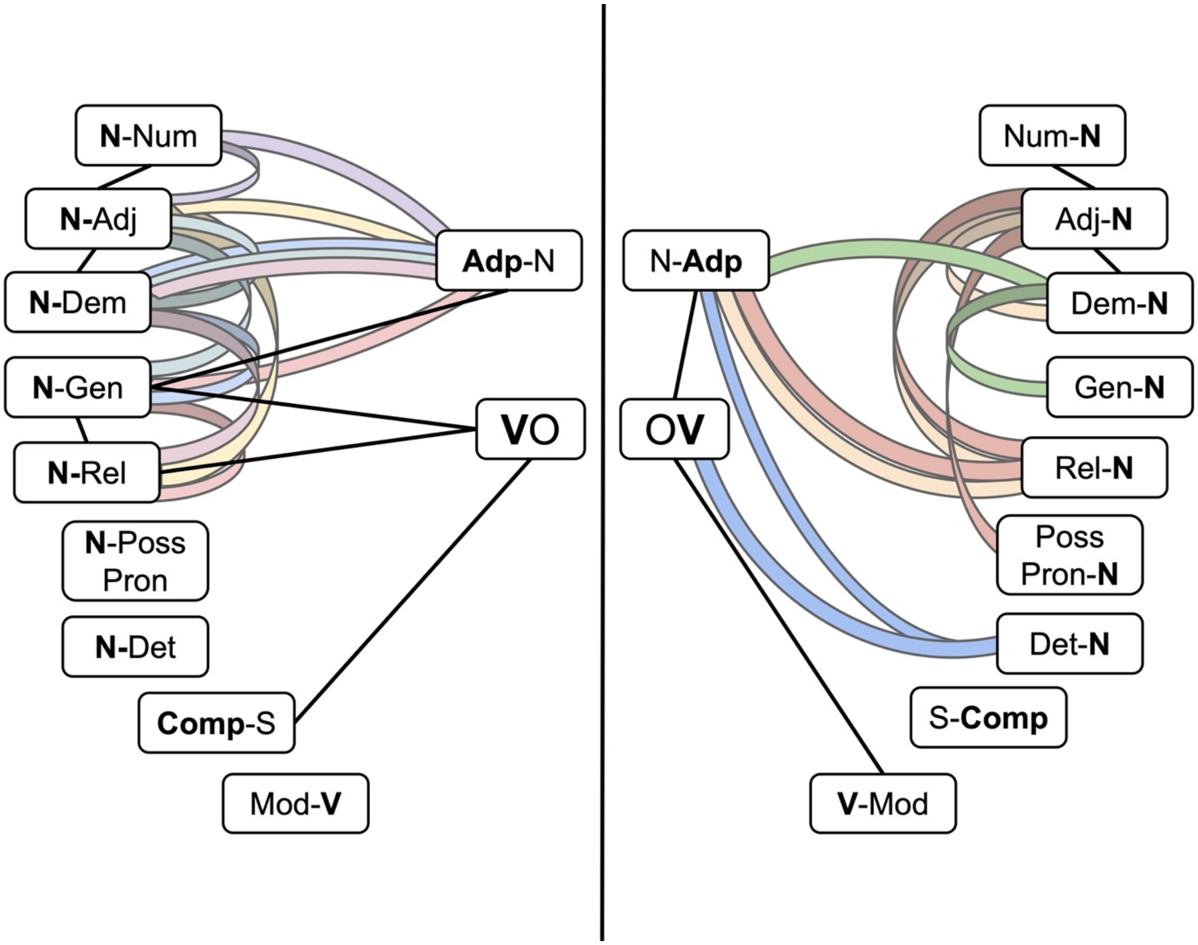

Figure S9: 21 out of 24 supported narrow word order universals in the current study.[1] *Simple universals (such as VO > N-Rel) are indicated by black lines. Complex universals with two or more word orders in the implication part (such as SOV & Det-N > N-Adp) are indicated by colored ribbons in the same color. Absence of connections between word order does* ***not*** *indicate absence of support for that correlation: unlike Dunn et al. (2011), we did not test for all correlations between a set of eight word orders, but only tested correlated word orders found in the* Universals Archive.

Now we return to explanations of narrow word order universals. For an illustration of how our results align with those in Dryer (1992, 2009), Dunn et al. (2011), Jäger & Wahle (2021), and Hartung et al. (2022), see Supplementary Information Text 8. The pattern that emerges from those studies, from others such as Hawkins (1983), Dryer (1992), Cysouw (2010), and Jing, Widmer, and Bickel (2021), and from ours is that the strength of narrow word order correlations differs depending on the type of head and dependent involved. The order of adposition and noun

[1] The Figure does not differentiate between N-Adp & (Adj-N|Rel-N) > Dem-N; Adp-N & N-Dem > N-Rel; and Adp-N & N-Dem > N-Adj; additionally, VS (tr. verb) > VS (intr. verb), Rel-N > N-Rel, and Adp-N & no SOV > N-Rel were not included.

is somehow central, as it is involved in the greatest number of supported correlations – Hawkins (1983: 297) has in fact already proposed that "Prep and Postp are more general typological indicators". Even for those who take the order of verb and object as the central pivot, VO languages and OV languages seemingly evolve differently; this is not a new finding, having been invoked in work on the Final-over-Final constraint (Sheehan et al. 2017, inter alia). Given that there seem to be two or three sets of 'densely' correlated constituent word orders that have 'loose' connections to other word orders (see Figure S9), we suspect that multiple motivations are at play; possibly all the ones summarized above *and* others. Further investigation needs to be devoted to pulling apart source-based explanations rooted in grammaticalization and language change from those rooted in cognitive preferences, having to do with processing ease and learnability. Our study cannot differentiate between these. However, our findings are in line with previous work on this topic, which highlights differential strength and interrelatedness of word order correlations.

Hierarchical universals.
This is a group of universals which consist of unidirectional chains of implicational universals that predict specific linguistic behavior, mostly with regard to a unified domain or paradigm. An example of a universal of this kind is "If the Dual extends to nouns, it also extends to pronouns" (Cuny 1906: 2; Gray 1939: 181; Jensen 1952: 9). The universals in this category are highly diverse when it comes to the principles that have been proposed to underlie their existence. For narrow word order, Hawkins' work has served as a fundamental bridge between cross-linguistic facts and ideas on processing efficiency. There has not been such an over-arching account of hierarchical universals. Unfortunately it is outside the scope of our current study to develop such a framework for all of the hierarchical universals we test. We will only provide a short overview of some of the principles that have already been proposed. Many of the suggested explanations relate to discourse structure, salience, iconicity, and economy (see the Introduction of Good 2008 on structural and external explanations). All of these are rooted in human cognition, some more overtly in the dyad of communication, others more abstractly in terms of what 'stands out' within the human experience and therefore deserves special marking.

In one of the many replies to the impactful paper by Evans and Levinson (2009) on language universals and universality, Haspelmath (2009) predicted one of the main outcomes of this study. This is illustrated in the title of his contribution: "The best-supported language universals refer to scalar patterns deriving from processing cost". Haspelmath gives several examples of such scalar patterns:

1. "*If a language has an [ø] sound (front rounded mid vowel), it also has an [e] sound (front unrounded mid vowel).*
2. *If a language has a [θ] sound (interdental fricative), it also has an [s] sound.*

3. *If a language allows two consonants at the end of a syllable, it also allows one or zero consonants at the end of a syllable.*
4. *If a language requires accusative marking on a patient object of some type, it also requires marking on patients of all types to the left on the following scale: speaker/hearer > 3rd-person pronoun > definite NP > indefinite specific NP > nonspecific NP.*
5. *If a language can form a relative clause on some clause position, it allows relativization on all other positions to the left on the following scale (Hawkins 2004): subject > object > oblique > possessor.*
6. *If a language uses a special reflexive pronoun for an adnominal possessor (e.g., Latin suum), then it also uses a special reflexive pronoun for the patient object (e.g., Latin se) [...].*" Haspelmath (2009: 458)

And as the title predicts, these are explained in terms of processing cost:

"*For example, the [u] sound requires more articulatory effort than the [s] sound; the [ø] sound is less easy to distinguish from other vowels than the [e] sound; accusative marking is less redundant on patient objects that are pronouns or definite than on indefinite and nonspecific NPs; and reflexive use is more remarkable (and hence better coded) in patient object position than in adnominal possessive position (cf.* He_1 *criticized* $himself_1$ *versus* He_1 *criticized* his_1 *colleague).*" Haspelmath (2009: 458)

Haspelmath (2009) is a short paper and no more details are given about what exactly processing cost entails, and how it captures seemingly diverse phenomena like articulatory effort, redundancy, and remarkability (the concepts mentioned in the second quote). We can assume, however, that "optimization of processing cost" is rooted in what have been called *competing motivations models* in linguistic typology. These have been widely applied (i.a. Croft 2003; MacWhinney et al. 2014) in accounts of linguistic variation and as a general framework for explaining cross-linguistic correlations. Kirby (1997; 1999) provides a compelling explanatory account based on the *competing motivations* model for a particular hierarchical universal: Keenan & Comrie's (1977) Accessibility Hierarchy. This proposed universal has been very influential and is represented below. Unfortunately, we were not able to test it in the current study as the elements involved are not tracked by Grambank dataset features.

Keenan & Comrie's (1977) Accessibility Hierarchy:
Subject > Direct Object > Indirect Object > Oblique > Genitive > Object of comparison

The Accessibility Hierarchy relates to what sorts of noun phrases can participate in relative clauses. It posits that types of NP roles in the corresponding matrix clause (sometimes referred to as "main clause") positioned towards the left hand side of this scale are more accessible for relativization (i.e. more likely to participate in a relative clause) than roles on the right. More

specifically, it predicts that if any role is able to be relativized, all of the roles to the left have to be able to be relativized too. So, subjects are the most likely NPs to be relativized and if oblique objects can be relativised, so can indirect objects, direct objects and subjects. If this hierarchical universal is true, we should not see cases of direct objects and genitives being relativised but not indirect objects. This is borne out by the cross-linguistic data presented in Keenan and Comrie (1977). For example, in Welsh, the primary strategy for forming relative clauses can only be used for subjects and direct objects (roles 1-2 above), while for English, all six roles can be relativized:

1. The woman who likes to eat cake (Subject)
2. The boy whom the teacher preferred (Direct Object)
3. The lady to whom the mayor awarded a special prize (Indirect Object)
4. The man with whom Alex went shopping (Oblique)
5. The lady whose medal was taken by the politician (Genitive)
6. The banker who the entrepreneur is richer than (Object of comparison)

Kirby (1997, 1999) explains cross-linguistic variation in relativization, as expressed by restrictions on which roles can be relativized given the Accessibility Hierarchy, as emerging from the interaction of parsing complexity (in comprehension) and morphological complexity (in production, less morphologically complex forms are preferred). Kirby experiments with a computational model where these two factors can be given various weightings: if morphologically complexity is high, that role cannot be relativized; if parsing complexity is high, the same strategy is used across roles. When weightings of both are left to vary, the simulation outcomes match the predictions of the Accessibility Hierarchy, for example by excluding the possibility that direct objects can be relativised while subjects cannot.

A famous formal account of cross-linguistic variation that matches well to the competing motivations accounts common in functional typology is Aissen's (2003) Optimality Theory account of Differential Object Marking (DOM). DOM is common cross-linguistically, and consists of patterns in a given language where some direct objects are marked for their role in the clause (e.g. with case or adpositions), while other direct objects remain unmarked. For example, in Spanish direct objects which are definite and denote human referents are preceded by the marker *a* 'lit. to' whereas other objects are left unmarked. See Spanish examples below from Fábregas (2013) for illustration:

1a	Encontré	un	problema.
	1SG.find.PST	INDF	problem
	'I found a problem'		

1b	Encontré	a	un	superviviente.
	1SG.find.PST	to	INDF	survivor
	'I found a survivor'			

Two principles (motivations) that interact with one another have been suggested to be relevant in explaining DOM: (i) iconicity, which in general holds that linguistic structure should reflect human experience of the world, and (ii) economy, which in general is a catch-all principle reflecting efficiency, the avoidance of redundancy, and the minimization of effort (Haiman 1983). In Aissen's account, iconicity implies that more prominent or affected objects (direct objects that are definite, human, or higher animates) are more likely to be case marked; while economy implies that case marking should be avoided altogether. Her analysis suggests that these two constraints interact in such a way that the outcome is the observed cross-linguistic variation in differential object marking: only direct objects that stand out in discourse (whatever that means in individual languages) are (case) marked.

For Aissen (2003), the constraints are hard-wired, and cross-linguistic variation is a result of differences in language-particular constraint rankings. Other formal approaches to hierarchical universals include, amongst others, Harley & Ritter (2002), Harbour (2014, 2016), Toosarvandani (2023) on feature geometric solutions of the feature hierarchy (see Supplementary Information Text 7), where the hierarchy emerges from the internal structure (the geometry) of features; Sigurðsson (2004), and others on morphological structure and phonological dependencies. For functional typologists, the same could emerge solely from language use. The prominence of certain direct objects (again, those that are definite, human, or higher animate) and their overt case marking can be explained in terms of the signalling of important discourse information. Economy is a very important principle in typology (Haiman 1983). This principle essentially states that linguistic structures that are predictable tend to be dropped or shortened, and has been linked to frequency (Haspelmath 2008a).

This brings us from the general idea of competing motivations to what these 'motivations' actually are. We already encountered economy and iconicity, for more on these see amongst others Haiman (1983), Haspelmath (2008a, 2008b), and below. They are two motivations that can be readily exemplified with how they matter given a range of different linguistic phenomena. Many other concepts that can be thought of as motivations are more vague. Cristofaro & Zúñiga (2018a: Sect. 2) provide an overview of synchronic explanations of hierarchical universals, and constant reference is made to language in use: overt marking is applied when 'needed' or 'more relevant', in order to 'disambiguate', and may be omitted when the referent is 'easy to identify'. Indeed, it seems that many linguists would intuitively connect hierarchical universals to communicative needs, or connect them to discourse-related concepts. One further example of

this is Givón's (1979) analysis of Keenan and Comrie's (1977) Accessibility Hierarchy. Givón argues that topicality and presuppositionality (to what extent the speaker may assume that the hearer knows what's being talked about) are in part responsible for the placement of certain roles on the Accessibility Hierarchy, most importantly those who require passivisation or promotion to direct object.

DuBois, Kumpf and Ashby (2003) contains several contributions where the nominal hierarchy (see Supplementary Information Text 7) and other cross-linguistic preferences similar to universals are explained in terms of salience. Salient entities are those that somehow stand out in discourse, that may be unexpected in that grammatical role, or especially potent. This may ultimately be related to how much empathy humans have towards those entities. Humans are probably most capable of understanding the feelings of other humans; and this may be related to the special marking of pronouns and nouns referring to humans. Moving down the cline of the nominal hierarchy, empathy is possible for animals (especially higher animals, to whom we regularly attribute human-like cognition and feelings), but much less so for inanimate objects.'Motivations' such as salience make us turn towards general human cognitive capabilities, but lack operationalisation in typology (but see Enger & Nesset 2011). See for example Kuno & Kaburaki (1977) on how empathy comes in, and Boswijk and Coler (2020) on what salience is in linguistics.

Croft's (1988) relational hierarchy is also worth mentioning here. One of the implementations of that hierarchy in the current study, 'Verbal indexation of object ⇒ verbal indexation of subject' (Croft 1988), received overwhelming statistical support, as well as several others that can be linked to the relational hierarchy. This universal can be understood as a reflection of the functional need to distinguish "the important or salient arguments", as subjects are more important to define than objects and therefore are more likely to be marked (Croft 1988). This generalization and others like it are rooted in a functional trade-off between hearer and speaker; where the hearer might prefer overt marking and redundancy, and the speaker might prefer null marking and shorter forms, decreasing their effort. This could again be explained in a competing motivations account, where iconicity and economy conflict and the outcome is marking of only "the important or salient arguments". Lastly, see Culbertson et al. (2020b) on pressures regarding the internal ordering of nominal modifiers that reflect how strongly different modifiers are associated with the head noun, rooted in how humans engage with the world.

One last example of a competing motivations account can be imagined for the agreement hierarchy (Corbett 1979), which details which phrases associated with the noun are most likely to carry syntactic agreement, such as '-*s*' in '*Zoë rides a bike*'. A highly supported hierarchical universal, Plank (1994)'s 'If determiners agree within NPs, modifiers are likelier also to agree than not to agree', is depicted in Figure S10 and a good candidate for an explanation in terms of efficient processing. On Corbett's (1979) agreement hierarchy, both determiners and adjectival

modifiers are considered to be attributive modifiers; however, adjectival modifiers are typically positioned closer to the head noun than determiners (Culbertson et al. 2020b). Since agreement may play a role in incremental processing, enabling discourse resolution, it makes sense for both determiners and modifiers to agree rather than solely determiners, which would imply ‘skipping over’ modifiers in dependent-head order languages. Economy would, of course, be the competing motivation going against agreement altogether.

One common view, then, is to emphasize the role of various motivations or wide-ranging explanatory concepts such as iconicity and salience, economy, and discourse structure, that compete and result in patterned variation across languages. Hierarchical universals simply reflect that some ordering of priorities for marking never occur, and hence such motivations must reflect fundamental aspects of human language. For more on competing motivations see various contributions in MacWhinney, Malchukov, & Moravcsik (2014), Bybee (2010), and others.

Several linguists, however, argue that we must look at diachrony of the markers and constructions involved: this case is clearly presented by Cristofaro & Zúñiga (2018b) with several examples, and others such as Gildea & Zúñiga (2016) for the referential hierarchy. This account can be explained using one of the supported universals in the current study: “If the Dual extends to nouns, it also extends to pronouns” (Cuny 1906). Given that the marking of the dual category is more common on pronouns than on full nouns, we could presume that marking of the dual on nouns could arise through generalization of number marking from pronouns to nouns. The parallel marking of the dual on nouns could be rooted in grammaticalization of the number word for ‘two’, similar to the origin of plural markers (Cristofaro 2012). See Kiparsky (2008) for a formal proposal that marries absolute universals and language change.

This all provides context for the discussion of the hierarchical universals that were formulated as implications and tested in the current study. The hierarchical universals tested in our study can be understood as specific instantiations of the broader hierarchies which were introduced in Supplementary Information Text 7: the relational hierarchy, the definiteness hierarchy, the nominal, the agreement hierarchy, the feature hierarchy and the number hierarchy. We find support for each of these hierarchies. Several of the individual universals are also closely related to others under the same hierarchy. We test five universals which can be categorised as relating to the broader idea of the definiteness or agreement hierarchy; they are all supported.

For the relational hierarchy, all associated universals are supported, except for one. This is Kozinsky’s universal, “If the prefix position is the only possible one for a subject-affix, then the object-affix also occupies this position in all forms (with the exception of Wolio) or at least in some of them (no exceptions)” (Kozinsky 1981). We tested two versions, a weak version: s prefix ⇒ o prefix, and a strong version: s prefix ⇒ o prefix & no o suffix. Of these, the strong version does not hold while the weak version does.

We test six universals associated with the broader category of the nominal hierarchy, of which only one is not supported: “If there is any inflection in nouns, there are also some inflections in pronouns”. In the Universals Archive, this claim is attributed to Moravcsik (1993), and data from Hurford & Kirby (1995), but these sources could not be retrieved. That being said, it is the most generally formulated universal out of those that relate to the nominal hierarchy.

We test six specific universals which relate to the feature hierarchy, of which two are not supported. The ones that are supported detail the relationship between the marking of number and gender: gender ⇒ number; gender Adj ⇒ number Adj; gender Dem ⇒ number Dem; gender Art ⇒ number Art. The two universals which are not supported deal with the relationship between the marking of case and number: nominal number ⇒ nominal case; case ⇒ number. These were operationalized in the best way possible, but nevertheless these were perhaps a bit opportunistic (both are originally proposed on the basis of a very small sample of languages) and could not be implemented in a manner that perfectly replicates the original proposal using Grambank data. Proposed by Campanella (1638), as interpreted in in Universals Archive (Plank and Filimonova 2006), nominal number ⇒ nominal case reads in full: “If nominals inflect for number, they also inflect for case.”. The other, case ⇒ number, proposed by Uspensky (1965: 211), based on Greenberg (1963: 103), reads in full: “Number is, as has been seen, closer to the stem and generally present when case is present, while the opposite relation holds far more rarely.” Especially regarding the latter universal, our implementation is imperfect because it does not reflect the order of inflectional morphemes. Ultimately, these results imply that when investigated in this manner, part of the core of this hierarchy holds; yet the marking of case, which was proposed to be a part of this hierarchy, is not constrained by and does not constrain number marking.

The last well-studied hierarchy we tested is the number hierarchy. Here, we have Trial ⇒ Dual and Dual ⇒ Plural, both from Greenberg (1963). The latter holds but the former does not. The reason for that is probably simply statistical power; having a trial is quite rare, in our dataset there are only fourteen languages with a trial, twelve of which also have a dual. It is highly likely that this universal would be supported with a larger language sample, given that the pattern suggests languages with a trial also have a dual.

The two remaining hierarchical universals are nominal inflection ⇒ verbal inflection; and suppletion noun ⇒ suppletion pron|verb; the former of which is supported while the latter is not. See Bobaljik (2012) on generative universals of suppletion, specifically, far more specific conclusions and theorizing on suppletion in comparative constructions.

In summary we can say that hierarchical universals receive overwhelming support, except for the strong version of Kozinsky's 1981 claim 's prefix ⇒ o prefix & no o suffix'; a very generally

formulated universal on nominal inflection; two universals on the relation between case and number, and a universal on suppletion. Greenberg's (1963) universal on trial number given dual probably simply does not hold because of statistical power. Those that are not supported are either on the periphery of the proposed hierarchies in the literature or are formulated in a 'too general' sense. What we mean here is that supported hierarchical universals are probably rooted in diachronic pathways that are informed by competing motivations (triggered by innate constraints or rooted in language use) *within specific paradigms*: they have to be formulated in specific terms or otherwise they become 'too general' and do not hold. The fact that the other generally formulated universal, nominal inflection ⇒ verbal inflection (Moravcsik 1994), *does hold,* simply speaks towards how it captures a general morphological pattern. Speculating, we could add that a reason for the unsupported universals related to case might be that case marking systems evolve and are syntactically realized very differently from systems marking person, number or gender; the latter through agreement, case through government (but see Baker 2015 for a different analysis of the morphosyntax of case systems).

The fact that hierarchical universals hold so well is probably best explained by a general competing motivations account; whether these are innate constraints or rooted in language use is an open question that our study cannot speak towards. The role of diachronic explanations and grammaticalization likewise is a topic for further research.

Broad word order universals.
The category of broad word order universals consist of generalizations where one element concerns word order and the other another element, e.g. "Verb-initial languages normally have no overt copula" (unpublished statements of Keenan's, reproduced in D. Payne 1990). Of the 72 broad word order universals we test, only 8 (11%) are found to be supported in both the spatiophylogenetic *brms* models and the *BayesTraits* coevolution analyses. Unlike narrow word order universals, the universals we have categorized as broad word order do not rely on the concept of harmonic structures in different parts of the grammar. Instead, this collection of universals is likely to be shaped by a diverse range of processes and explanations. It goes beyond the purposes of the current study to give a full overview of these here, but we outline a few that have been especially prominent in the literature. It should also be noted that quite a few proposed but unsupported broad word order universals are rather opportunistic and did not come with a theoretical basis or explanation in the first place – a few of these are listed below.

Several authors have proposed relationships between affixal morphology and word order. We investigated five of these: suffixes > N-Adp, and prefixes > Adp-N (both Greenberg 1963); VO > prefixes, and OV > suffixes (both Lehmann 1973); V-final > N-Adp & suffixes (Bybee, Pagliuca, & Perkins 1990: 30). None of them are supported. Hawkins & Gilligan (1988) propose a potential explanation in terms of the Head Ordering Principle, a processing-based account. While the authors of the universals do not overtly comment on this, a grammaticalization

account has been proposed for these by others, here we refer to Mithun (2003) who gives an exhaustive summary and at the same time, problematizes it. Mithun (2003) finds that while many affixes indeed retain the position the free-standing word was in before grammaticalization ensued, but functional changes in source material and different diachronic processes complicate the matter greatly. She specifically states that there is very little evidence for affixes to change position in the face of word order change, which would be expected given cross-category harmony, the functional explanation that has been invoked for correlations between word order and affix position. All in all, we may conclude that specific types of markers may indeed evolve in such a way that they derive from free-standing words and simply retain the relative position, but that these broadly construed universals are not supported. This is in line with Mithun (2003) and later work such as Jacques (2013). See Koplenig et al. (2017) and Nijs, van de Velde and Cuyckens (2025) for more on the topic of interactions or trade-offs between morphology and syntax.

Other entire groups of universals that are almost entirely not supported are those predicting various characteristics for verb initial languages, a group of supposed universals that relate word order to various agreement phenomena (Foster & Hosfling 1987, Kozinsky 1981), another big group that predicts various characteristics for languages with either free or rigid word order (Girard 1747 and Beauzée 1767, as interpreted in the Universals Archive, Plank and Filimonova 2006). Some of these are very opportunistic; such as free order > no Adp-N (Girard 1747) or rigid order > N-Adj (Beauzée 1767). One last, further example here is the relation between word order and the passive, proposed by Lehmann (1978b).

However, there is one group of universals for which we find partial support, and that we can discuss at more length in terms of possible explanations, given that it has been widely researched from a variety of angles. This group deals, in various ways, with the presence or absence of grammatical case and various associated features, mostly involving the order of adposition and noun, the order of verb and object, and free vs. rigid word order. Several are from Stassen (1997), who posits relationships between the presence or absence of morphological tense, the presence or absence of case, and the order of verb and object. Another set is from Girard (1747), as interpreted in the Universals Archive (Plank and Filimonova 2006). There are thirteen of these, and five are supported:

- case > no Adp-N; no case > Adp-N (both from Girard 1747, as interpreted in the Universals Archive (Plank and Filimonova 2006)
- no case > no morph tense | VO; VO > no morph tense | no case (both from Stassen 1997)
- V-final | free > N-Adp | case (Rijkhoff 1992: 249)

While eight are unsupported:

- no morph tense -> no case | VO; morph tense > case | OV; OV -> morph tense | case; case > morph tense | OV (all four from Stassen 1997)
- N-Adp > case noun (Stolz 1992: 75)
- free order > case noun | Pron (Beauzée 1767)
- free order > case (Girard 1747, as interpreted in the Universals Archive (Plank and Filimonova 2006)
- SOV|OSV > case (Greenberg 1963)

These universals revolve around strategies to resolve argument relations, of which, in the world's languages, fixed (SVO) word order, grammatical case marking, and verb agreement are among the most common solutions. These have been widely investigated and largely found to be supported by a range of different studies, including Greenberg (1963: 96), Nichols (1992: 108), Siewierska & Bakker (1996: 136), Kiparsky (1997), Dryer (2002), Gibson et al. (2013), Sinnemäki (2014), Futrell et al. (2015), Levshina (2019), and Shcherbakova et al. (2024). What is interesting in our investigation of claims taken from the Universals Archive, is that the supported universals point towards the association of:

- no case: Adp-N & (VO OR no morphological tense)
- case: no Adp-N
- V-final | free word order: N-Adp | case

The existence of free word order OR verb-final word order OR postpositions alone does not predict the presence of case; the existence of case does not predict the presence of morphological tense or OV word order; OV word order does not predict morphological tense OR case; and presence or absence of morphological tense is not indicative of case or specific word order. The tendency for case marking on core arguments to be present in verb-final languages or free word order languages has been intensively studied for many years by linguists explaining the combinations of those features by generative grammar (Kiparsky 1997), ease of processing (Greenberg 1963, Siewierska & Bakker 1996, Nichols 1992, Gibson et al. 2013, Futrell et al. 2015, Polinsky & Magyar 2020), and a complexity trade-off (Sinnemäki 2014). A more recent account by Shcherbakova et al. (2024) models three variables within the same model controlling for phylogenetic non-independence and supports the long-proposed correlation between case and both word order characteristics (verb-final word order and free word order). The involvement of adpositions, especially postpositions, can probably be explained as they are one of the diachronic sources of case systems (Kulikov 2006) and can be equated under some accounts of argument marking, such as that of Hawkins (2002) (see Dryer 2002). To summarize: strategies to resolve argument relations, as related to case marking, postpositions, free word order and verb-final order are correlated in intricate ways; possibly in a trade-off relationship (see Berdicevskis, Schmidtke-Bode and Seržant 2020 on a similar trade-off).

This leaves three other supported broad word order universals:

1. rigid order > SV (Beauzée 1767). This supported universal belongs to a group of thirteen universals that predict the behavior of languages with rigid vs. free word order; and it is the only one to be supported. This universal may be connected to the previous discussion of strategies to resolve argument relations, as rigid order has been proposed as one of those strategies. This idea is summarized by Levshina (2019: 545). For argument relations to be resolved, rigid order languages should have arguments on either side of the verb. This is actually what we tested: our implementation is along the lines of rigid order > verb-medial, as Grambank does not contain data on the placement of the subject or object with regard to the verb. However, since OVS languages are exceedingly rare, we do effectively test Beauzée's (1767) claim. Here, the explanation is potentially grounded in the same principles as the discussed correlations above. It is in any case fundamentally clear that positioning the verb between the two major arguments of a transitive sentence is the only word order that can work as an argument role disambiguation strategy (Siewierska & Bakker 1996), as in verb-initial and verb-final word orders, subject and object can be mistaken for each other.

2. switch-reference > V-final (Haiman & Munro 1983: xv). There are several universals that predict characteristics for verb final languages, such as V-final > postpositions & suffixes (Bybee, Pagliuca, & Perkins 1990: 30) – none of these hold. However, in the opposite direction, the claim that languages with switch-reference are "mostly verb-final" (according to Haiman & Munro 1983: xv) holds. Here, multiple explanations are possible. The original authors (Haiman & Munro 1983: 107) comment that in verb-final switch-reference languages, switch-reference marking is suffixal, hence appears *immediately before* the reference clause. This is of course the place where it is most relevant from a processing perspective; the marking of switch-reference at that point in discourse is most useful. Speculatively, it might also be the case that switch-reference markers evolve from markers of person or number, which might have specific properties in verb-final languages that lend them well to becoming switch-reference markers.

3. SVO > not ergative (Schwartz 1972: 32, Trask 1979: 385). This supported universal belongs to a group of universals that link word order to alignment; none of the following are supported: ergative > VSO | SOV (Schwartz 1972: 32, Trask 1979: 385), ergative case > Gen-N & N-Adj (unknown source), non-acc align > V-initial (Nichols 1992: 113). Trask (1979) provides two detailed grammatical pathways through which the two distinct types of ergativity he identifies could emerge. He admits that the word order patterns he associates with the presence and absence of ergativity are harder to explain than other features that align with ergativity, but nevertheless comes up with a sound theoretical idea, rooted in typical pathways of linguistic change. As far as we know, there has not been a study further investigating Trask's (1979) hypothesis; Siewierska (1996) likewise posits there is no further investigation on the matter; nor do more recent publications citing this work.

What is striking about these four supported universals is that they are again very specific (see the discussion on hierarchical universals). The possible explanations for them are also rather specific and rooted in complex interplay between language-in-use type explanations (in other words, speakers of languages need some way to resolve argument relations) and diachrony (see Trask's 1979 account of the emergence of ergativity). This may support the case that the universals with the strongest support are those that are founded in very specific motivatios rather than more general theories.

Other universals.
Finally, our 'other' category includes a diverse set of proposed universals that range from relatively specific morphological dependencies to associations involving clause-level phenomena and multi-clausal constructions. Of the 24 universals we test classified as 'other', just four are supported (17%). Because these target such a wide range of morphosyntactic phenomena, the processes that potentially shape them are also more disparate. Some of the unsupported universals that have been put forward have been proposed from an opportunistic perspective; examples are: SVCs (serial verb constructions) > little inflection (Lord 1993) and N-poss suffix > no PRS overt copula 'to be' (Serebrennikov 1974). Others may never have been intended as universals, such as switch-reference > accusative (Aikhenvald & Dixon 1998) or no case (noun) > article (Renzi 1992).

Of course there are some exceptions to this as well. Some of the 'other' universals relate to a general inflectional morphological profile of the language. Three of the supported ones can be said to be of this type: Gen prefix > verbal prefixes (Cutler, Hawkins, & Gilligan 1985: 730; Hawkins & Gilligan 1988: 224, C.Lehmann & Moravcsik 2000), agreement possessive heads > subject verbal agreement (Keenan 1974: 303), and less case > more tense (Serebrennikov 1974: 300). Note that the latter is supported with negative coefficients in the mixed effect models, suggesting that the association is actually between less case and **less** tense. These three universals capture general inflectional morphological profiles: languages that have prefixes on nouns and on verbs, languages that have agreement on heads within the noun phrase and the verb phrase, and isolating languages.

As for the first supported universal in this category, Gen prefix > verbal prefixes, we should first note that our implementation is quite strong and specific, as the original formulation states: "There is more prefixing on verb than on noun. If a language has any prefixes on noun, it will also have prefixes on verb with considerably more than chance frequency" (Cutler, Hawkins, & Gilligan 1985: 730; Hawkins & Gilligan 1988: 224, Lehmann & Moravcsik 2000). This is simply a consequence of the Grambank features. The next thing that is interesting about this universal is that markers of possession have a very different distribution depending on whether they are placed on the dependent or on the head: adnominal possession marked by a prefix on the

possessor is quite rare (Grambank feature GB430), while adnominal possession marked by a prefix on the possessed noun is more common but almost exclusively in the Americas (Grambank feature GB431). The two features were conflated for the analysis of this universal. Note that Dryer (2013) calls the latter "perhaps the clearest apparent example in this atlas of an Old World - New World split in the distribution of the two types of possessive affixes: while possessive suffixes are the primary type in the Old World, possessive prefixes are primary in the New World."

The distribution of possessed nouns marked by prefixes matches the distribution of prefixal person markers quite closely (see Grambank features GB079, GB090, GB092, and GB094). To the best of our knowledge, people have remarked that these tend to be prefixal (see Birchall 2014 and references therein), but nobody has made the link between prefixal markers on possessed nouns and person markers on verbs. Since these both encode person, they may be linked through grammaticalization (marking of pronominal markers with heads), but this is just speculation. Grossman and Polis (2018) provide a diachronic account of the predominantly prefixal nature of Ancient Egyptian-Coptic, showing that it does not rely on any unusual processes (see also Harris 2008).

As for the second of these, "If heads of possessive constructions agree with their possessors in a given language then verbs agree with subjects in that language" (Keenan 1974: 303), the key paper is Nichols (1986). In that paper, Nichols describes the existence of head-marking and dependent-marking languages, meaning that languages have the tendency to either mark grammatical information about possession, person, number, and grammatical role on heads or on dependents (the other logical options also occur but are less common). The supported universal is a clear reflection of this tendency. Nichols (1986) argues that head-marking is favored even though dependent-marking occurs more frequently. She gives several specific reasons for that, mostly from a linguistic perspective, dealing with flexibility in terms of word order, the retention of head-marking in simplification processes, and from a diachronic perspective, showing that head-marking arises from a greater variety of sources than dependent marking. Interesting for the current universal, verb agreement (head-marking of argument roles) is also common in otherwise dependent-marking languages, so it is clear that this is a unidirectional universal. The potential cognitive or communicative benefits of being consistently head-marking have, however, received little attention, nor has a cohesive body of theoretical literature about specific diachronic pathways for head-marking emerged. The famous follow-up to Nichols (1986) is Nichols (1992) (see the critique by Cysouw 2002), which links head- and dependent-marking to other typological features and explains global patterns in terms of areal divergence.

The third supported universal, by Serebrennikov (1974), attests to a correlation between less case and less tense, basically identifying as a type so-called isolating languages that (probably) do not have a lot of inflectional morphology. As can be observed in Figure S12, this universal is not as

categorically supported as some of the other universals, as we observe frequent and stable pairing of features predicted by the universal to be uncommon, when both are gray or when both are red. However, there is a tendency towards less case marking (red) to be associated with less tense marking (gray) in several places on the global phylogeny, including in Indo-European, Austroasiatic, and Austronesian. Again, it goes beyond the space we have for discussing our findings to give a full overview of morphological typology, for the widely accepted comprehensive account, we refer to Bickel & Nichols (2007). One possible explanation for the support for this universal is the sociolinguistic account that has been given for the lack or reduction of inflectional morphology. Both features, case markers and tense markers, can be challenging for adult second-language learners to acquire, which can lead instead to the learners relying on simplified markers or omitting them altogether. It has been argued that in communities with large proportions of second-language learners this can result in reduction of inflectional morphology (Lupyan & Dale 2010, Sinnemäki & DiGarbo 2018, Sinnemäki 2020), including case and tense markers.

So while these three mark some core universals of morphology, we should note that there are four morphological universals that are not supported:

- (person)-number (verb) > tense-mood (verb) (Greenberg 1963)
- more case > less tense (Serebrennikov 1974: 300)
- nominal case > verbal inflection (Campanella 1638, as interpreted in the Universals Archive, Plank and Filimonova 2006)
- SVCs (serial verb constructions) > little inflection (Lord 1993: 235)

So if the three supported ones above speak towards a general morphological profile, why do these not hold? Here we fall short of real answers and can only speculate. We suspect that, similar to hierarchical and word order universals, changes to morphology that lead to correlations are *specific* types of changes, rooted in linguistic principles such as were affix origins, agreement patterns, and head-dependent structure.Usage-based explanations such as those rooted in simplification-through-L2-learning also seem worthy of more attention. More research on this is clearly needed.

That leaves the last supported universal in this category, “An opposition of "alienable" and "inalienable" possession is almost inevitable in languages where possession is regularly head-marked” (Nichols 1988: 576, Nichols 1992: 119). Nichols (1992) presents, in our interpretation, a grammaticalization account of the emergence of split alienable and inalienable possession. Since it is the head noun that is possessed, it follows that its semantics could start determining what possessive construction is used. It is also possible that independent pronouns evolve into inalienable markers on head nouns, given that inalienable nouns are often obligatorily possessed. Another potential explanation is iconicity: head-marked alienable nouns may form shorter

expressions, marking the close relationship between referents of inalienable nouns and their possessor.

The global conclusion we can draw from the ‘other’ category is that the correlations we find support for are relatively specific ones that all relate to morphology; and even more strongly, they do not connect a morphological feature to a non-morphological feature – we investigated some universals like this, but none of them are supported. Coupled with the lack of support for broad word order universals that broach correlations with prefixes and suffixes, this may imply that there are few universals that impact both morphology and syntax, except for where they touch upon one another in argument resolution, or where they are involved in complexity trade-offs (see above).

Concluding remarks regarding explaining universals.
In the preceding sections, the view may have emerged that there are several different types of explanations that are mutually exclusive, of which only one can be ‘true’. This view is mistaken: the field has long recognized that explaining implicational universals is hard and dependent on many interacting factors (i.a. Hawkins 1979, LaPolla 2002, contributions in Good 2008, see below). Haspelmath (2024), for example, describes how a range of phenomena can potentially be explained by three different kinds of explanations: structural, evolutionary, and biocognitive.

What we find most intriguing, given the methods we apply in the current study, is the integration of explanations rooted in diachronic processes and other types of explanations, whether they be formal, functional, or something else. While investigating diachrony hand-in-hand with other types of explanation (processes, mechanisms, etc.) was methodologically impossible in typology before Maslova (2000) and Dunn et al. (2011), it is possible now; typology as a field may take the input from the earlier literature and develop this line of thought further. This view echoes several sources, cited here at length:

Bybee, Perkins and Pagliuca (1994), from their book “The evolution of grammar: Tense, aspect and modality in the languages of the world”, p. 300:

“*Our view, then, is that grammaticization is not goal-directed; grams cannot "see" where they are going, nor are they pulled into abstract functions. The push for grammaticization comes from below – it originates in the need to be more specific, in the tendency to infer as much as possible from the input, and in the necessity of interpreting items in context. This means of course that the grammaticization process has the potential for revealing a great deal about the psychology of language use, but it also means that the method for studying grammaticization must at once be cross-linguistic and context-oriented.*”

Mithun, from her 2003 article "Why prefixes?", p. 182:

"*Without an awareness of the individual histories of affixes, attempts at general explanations for their positions are bound to fail much of the time. But generalizations are not impossible. Some of the seemingly idiosyncratic developments seen here reflect deeper, general principles. The development of adverbial prefixes and adverbial suffixes in Kawaiisu from different members of the same compound construction shows that headedness does not determine which roots will develop into affixes. But the same development suggests that semantic generality and frequency in speech might. The frequency factor has also shaped the Choctaw pronominal affix paradigm in a complex way, resulting in pronominal prefixes for the entire paradigm except for the first person agents. Explanations of both the observed suffixing preference and the occurrence of prefixes are surely not beyond our grasp. Our generalizations will suffer, however, if we fail to take into account the individual histories of the affixes we are seeking to describe.*"

Moravcsik, from her 2007 article "What is universal about typology?", p. 37-38:

"*But is there a causal explanation for why a language system is the way it is? The causal explanation cannot be in terms of individual acquisition since the acquisition process presupposes the system as it is. Similarly, it cannot be in terms of individual language use since that, too, draws on the system. Nor can language function be directly invoked as a cause since there is no temporal process to connect function – the goals and resources of a human being in acquiring and using language – to the system of language he acquires and uses. As Dryer points out (2007: 246), even though language function is often cited as an explanation for language structure, this must be interpreted as a shorthand for language function explaining diachrony which in turn explains structure. Indeed, the only possible causal explanation for a language system is by reference to history: how a given system evolved from something else.*"

Harris, from her 2008 chapter "On the explanation of typologically unusual structures", p. 76:

"*I have argued that two specific typologically unusual constructions in two languages of the Caucasus are due to the unusual co-occurrence of quite usual processes. The more changes are involved, the less likely all will happen to co-occur. Typologically unusual constructions can be explained in terms of their origin. This approach explains both the fact that such constructions are unusual and the fact that they occur at all. It is the fact that so many specific factors or changes must co-occur or occur sequentially in an appropriate order that explains the infrequency of these constructions, and no further explanation is needed. Many typologically unusual constructions can be explained as uncommon combinations of common changes. In this sense, they are the result of historical accident.*"

Nichols (2008), concluding remarks from Good's (ed.) (2008) book Linguistic universals and language change, p. 287-288:

"*A consensus view would appear to be that, rather than synchronic patterning always being the goal and driving force of language change, various synchronic patterns are the predictable consequences of diachronic processes which have their own logic independent of the synchrony they produce. Thus, to a greater extent than Greenberg probably had in mind, synchronic structural patterns are epiphenomenal. But they are not entirely so. Economies of various kinds appear to be targets of change (as shown by Haspelmath, Garrett, and Albright), and there appear to be pure structural patterns that may be goals of change but are not its accidental results: Kiparsky's D hierarchy, word-final neutralization, stress-weight covariation; perhaps some of the word-order patterns, if they can be stated non-framework-internally; and the affinity between serialization and non-inflection that Hopper describes. This may be a consensus, but is this state of knowledge durable?*"

And two more quotes from Bybee, who has, probably more than any other functional linguist, focused on the role of universals in a holistic approach to what human language is. From her 2006 chapter "Language change and universals", p. 194:

"*I have argued for the necessity of taking diachrony into account in the formulation of language universals. In my view, linguistic theory must look beyond synchronic generalizations about particular language states to the formational mechanisms that bring linguistic structure into being. Language states come about through the complex interplay of processes at work as language is used. To assess the place of language in the context of human cognitive abilities, it is important to note that most of the processes at work as language is used apply to non-linguistic activities as well. Thus automation, habituation, and categorization can be seen to operate in non-linguistic abilities. Language is highly evolved but not totally distinct from other neuromotor and cognitive abilities (Bybee, 1998).*"

And on the last page of her 2015 book, *Language change*, p. 263:

"*[...] the way it works is this. No one intends to change language, but language users intend to communicate, and they use all the means at their disposal to do this. They are human and so their cognition and social awareness and goals come into play. As these are shared by language users, the same cognitive and social processes play out over usage events in all languages. Within a community, the accumulation of usage events as they are affected by production pressures, access of lexical and grammatical memories, productive use of these memories, and the necessary implications and inferences that go along with all communication create strong patterns across speakers and situations. In a positive feedback loop, these patterns affect*

cognitive or memory representations of language users and in this way affect future usage events. Thus languages are always changing but, in broad form, always staying the same."

These studies are not meant to give an exhaustive overview, but: given that most of them appeared in the 90s and early 2000s, we may state that Dunn et al. (2011) was extremely timely: they proposed the methods central in our study can be used in linguistics, methods that allow us to make diachronic inferences about synchronic distributions. (The same, of course, applies to other statistical methods to study typological distributions, which arose around the same time – see, amongst others, Bickel 2008, 2013, 2015 on the Family Bias method, and Sinnemäki 2010, Cysouw 2010 and Bakker et al. 2011 on generalized linear mixed-effect models (GLMMs).) Dunn et al. (2011) may have received a lot of critique (see replies to it in *Linguistic Typology* vol. 15, 2nd issue), but if it has (only) worked to put language change and statistical methods for typology solidly on the table, its importance is quite clear. If we are to build a holistic theory of why human language is the way it is, all components mentioned in this section have to be considered: (1) human cognitive endowment for language and everything else that humans do with their brains, especially considering things that human brains are good at and what they are bad at, (2) how first, second, and nth languages are learned, (3) communicative situations and goals that affect language use, and (4) language change. Not all of these components have received equal amounts of attention, and few people have focused on how they should interact in a holistic theory of human language. Next steps that can be taken are taking these methods (and others) in hand, and attempting to assess the relevance of these components and others for a unified theory of human language.

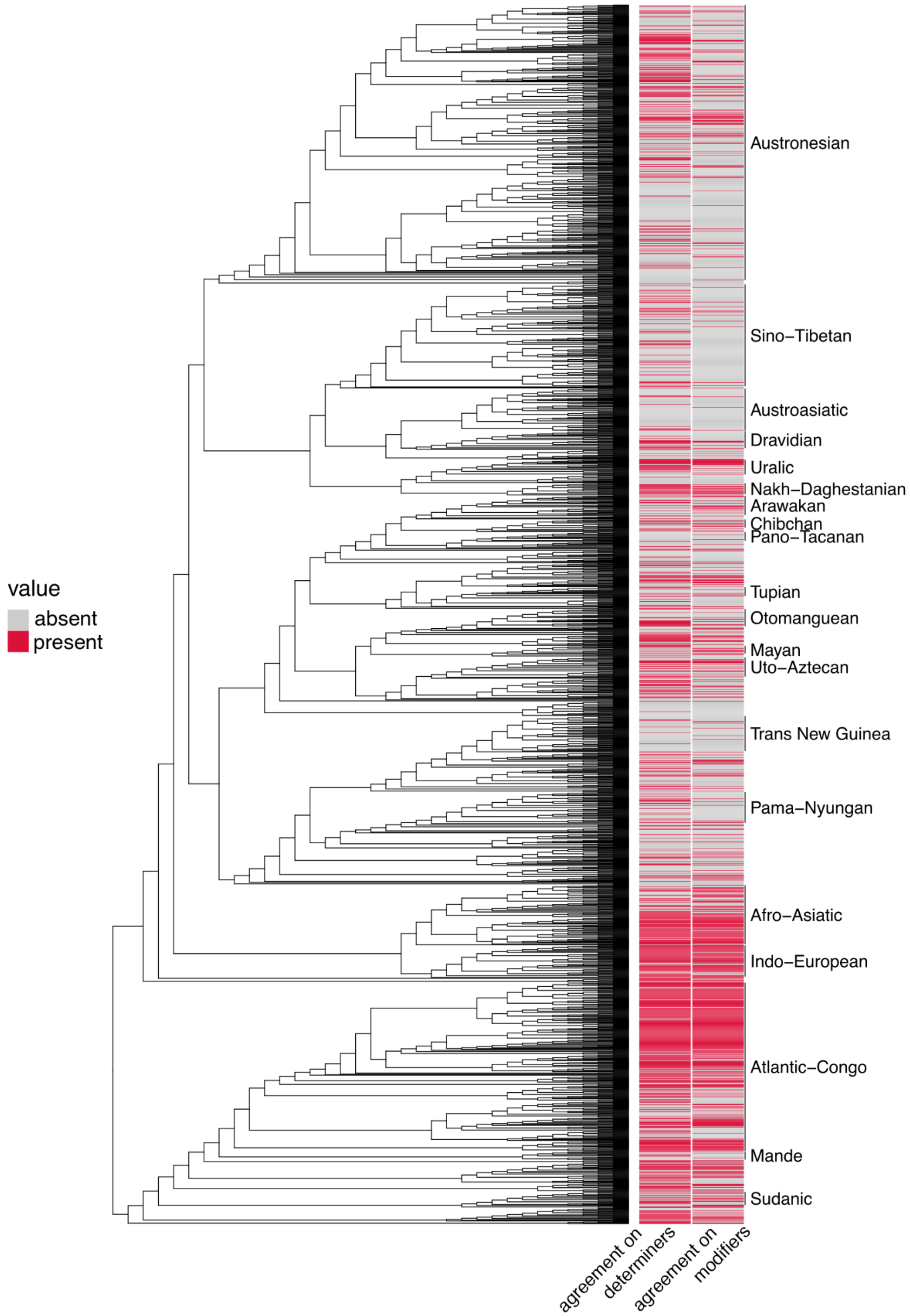

Figure S10: Dataset on adnominal agreement on 2226 languages plotted onto the tips of the tips of the world tree, illustrating the universal "If determiners agree within NPs, modifiers are likelier also to agree than not to agree." (Plank 1994). Note that the MCC tree is taken from a posterior sample of 902 trees, and support for higher order branches is extremely low.

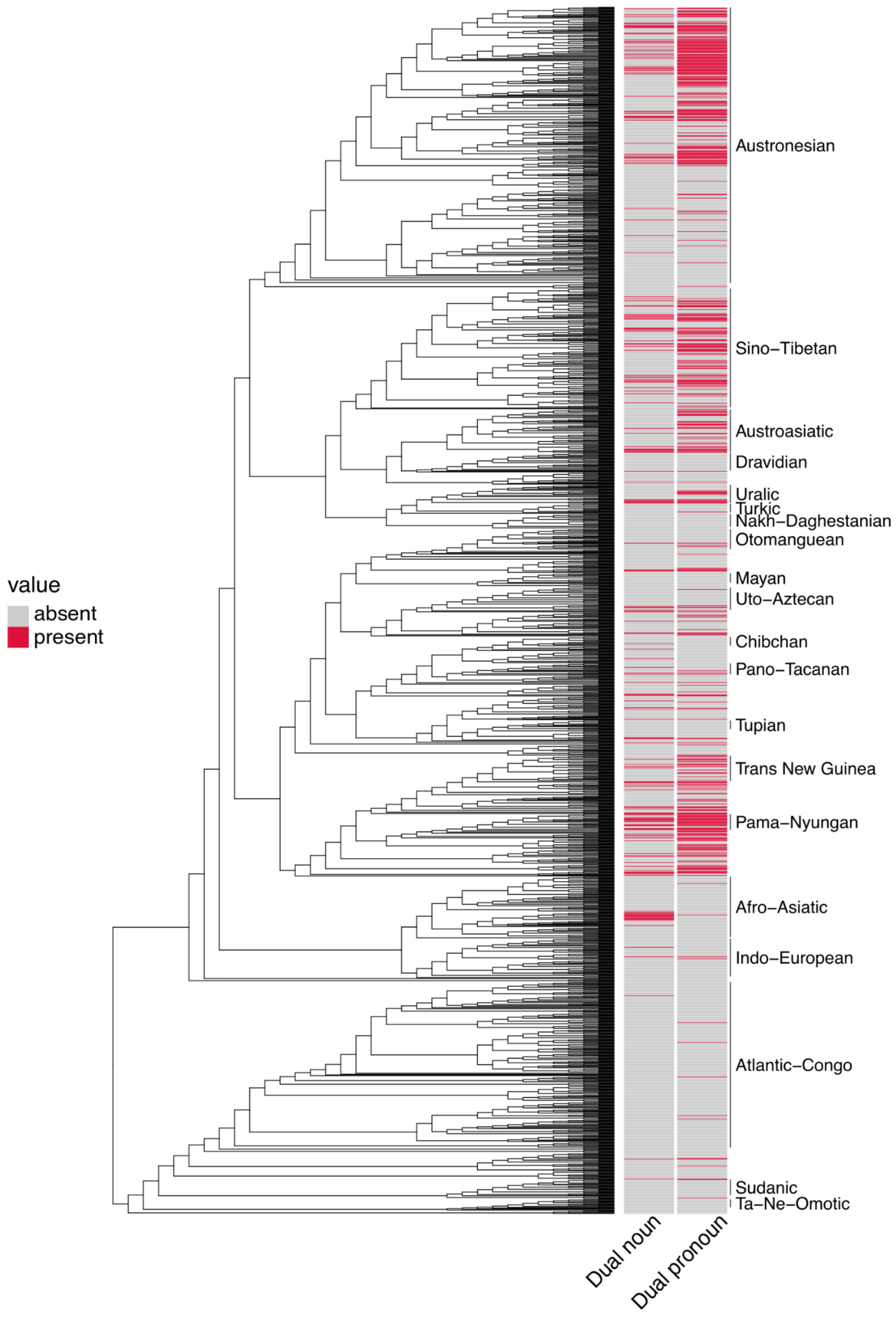

Figure S11: Dataset on number from 1777 languages plotted onto the tips of the tips of the world tree, illustrating the universal "If the Dual extends to nouns, it also extends to pronouns" (Cuny 1906). Note that the MCC tree is taken from a posterior sample of 902 trees, and support for higher order branches is extremely low.

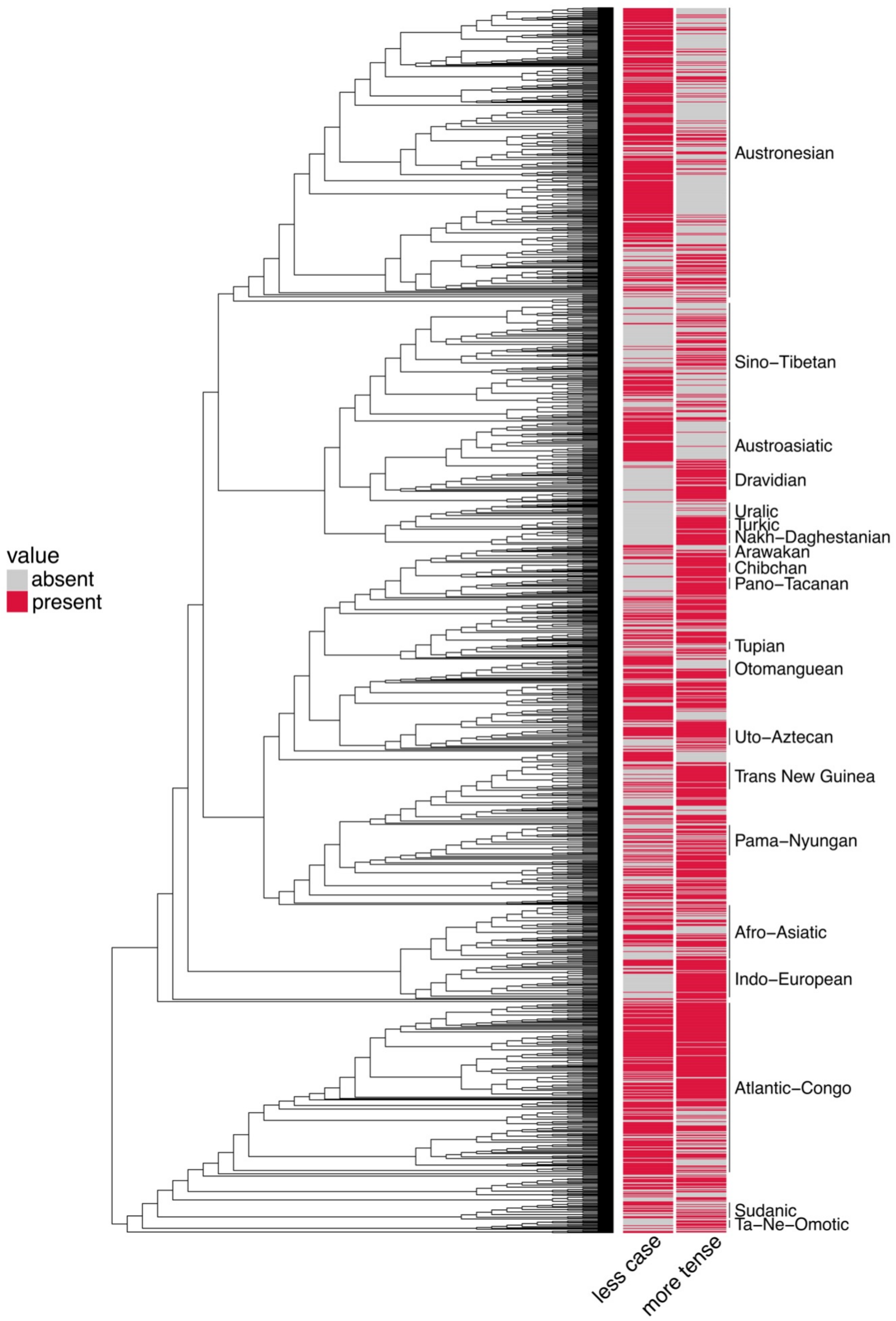

Figure S12: Dataset on number from 1744 languages plotted onto the tips of the tips of the world tree, illustrating the universal "The more developed a case system is, the less is its system of verbal tenses" (Serebrennikov 1974), implemented as less case ⇒ more tense. Note that the MCC tree is taken from a posterior sample of 902 trees, and support for higher order branches is extremely low.

Supplementary Information Text 10: Modeling the results in terms of phylogenetic distance, spatial distance, and macro area

In order to investigate gradient support for universals, we created linear models explaining the support for universals (for the spatiophylogenetic tests performed in *brms*, the median of the main fixed effect estimates; for the phylogenetic coevolution analyses in *BayesTraits*, the median Bayes' Factor (BF) relating the fit of the dependent vs. the independent model) in terms of the random effects included in the spatiophylogenetic correlations. The latter included 1) phylogenetic covariance, 2) spatial covariance, 3) intercepts on macro-area, and 4) slopes on macro-areas, calculating medians of these over the analyses conducted on 100 phylogenetic trees. The macro-area are Africa, Australia, Eurasia, Papunesia (Island South East Asia, New Guinea and Oceania minus Australia), North America, and South America, taken from Glottolog v. 4.3 (Hammarström et al. 2020). Analyses were conducted using *lme4* (Bates et al. 2005) in R (R Core Team 2021), with p-values calculated using the *lmerTest* package (Kuznetsova et al. 2017). Full results are included in Table S7 and S8.

Table S7: Results for modeling the median of the main fixed effect estimates from *brms* in terms of the random effects

	Estimate	Std. Error	t value	Pr(>|t|)
Intercept	1.08	0.31	3.47	0.0007 ***
SD phylo covar	-0.01	0.03	-0.26	0.79
SD slope macro-areas	0.62	0.24	2.62	0.009 **
SD spatial covar	-0.14	0.15	-0.94	0.35
SD intercept macro-areas	-0.12	0.18	-0.66	0.51

Residual standard error: 1.308 on 186 degrees of freedom; Multiple R-squared: 0.04201; F-statistic: 2.039 on 4 and 186 DF, p-value: 0.091

While R^2 values are small and it is clear that these models do not contain deep explanations of the level of support different universals receive, there is a clear positive effect for the standard deviation of the slopes of the macro-areas; i.e. better supported universals have higher variation in the macro-area slopes. Figures S13 and S14 demonstrate that this effect is only present in a subset of the data. For those universals that were supported on both analyses (n = 60), we observe one group for which the median slope SDs are between 0.25 and 0.9, with no relationship to the median estimate of the main fixed effect (Figure S13) or median BF (Figure S14), in other words, the strength of the universal. But from slope SD > 0.9, we do observe a linear trend indicating an increase of the median slope SD as the median main fixed effect

estimate (Figure S13) or median BF (Figure S14) increases. This positive relation suggests that there is a tipping point in how diverse macro-areas can be, with a subset of the universals that receive high support displaying different patterns across macro-areas.

Table S8: Results for modeling the median BF from *BayesTraits* in terms of the random effects

	Estimate	Std. Error	t value	Pr(>|t|)
Intercept	34.39	9.21	3.73	0.0003 ***
SD phylo covar	-0.65	0.89	-0.73	0.46
SD slope macro-areas	18.86	6.98	2.70	0.008 **
SD spatial covar	-10.85	4.58	-2.37	0.018 *
SD intercept macro-areas	-3.81	5.29	-0.72	0.47

Residual standard error: 38.76 on 186 degrees of freedom; Multiple R-squared: 0.084; F-statistic: 4.23 on 4 and 186 DF, p-value: 0.003

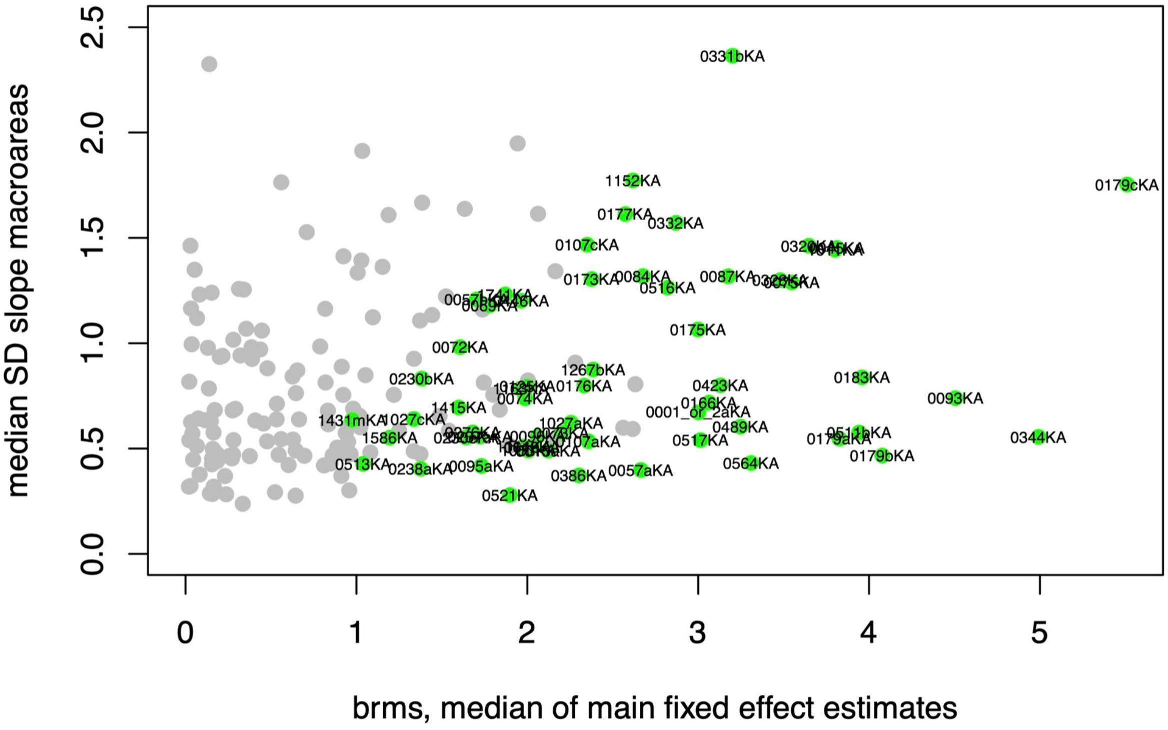

Figure S13: Estimates of the main fixed effects from the spatiophylogenetic analyses plotted against the median SD of the slopes of the macro-areas for 191 universals. Supported universals are in green; non-supported ones in gray.

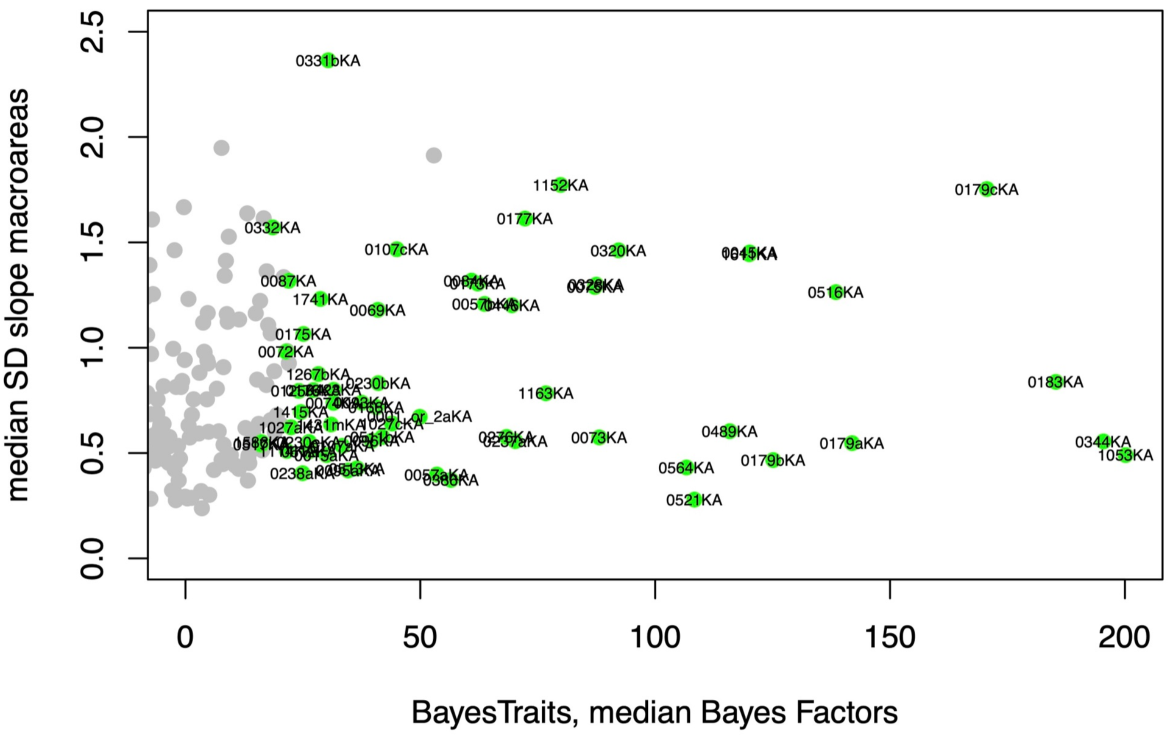

Figure S14: Median Bayes Factors from the phylogenetic coevolution analyses plotted against the median SD of the slopes of the macro-areas for 191 universals. Supported universals are in green; non-supported ones in gray.

In the rest of this section, some Figures and Tables are given, illustrating strength and differences between macro-areas, if any, across a selected set of universals.

Table S9 and Figure S15 relate the universal "If a language has Prep word order, then if the adjective follows the noun, the relative clause follows the noun" (Hawkins 1983: 74), with median main fixed effect 2.56 (median 95% CI 1.09 - 4.10), median BF = 19 (lower bound of the 95% High Densitity Interval is just below zero (9.75) , and therefore this universal is not supported). This universal displayes no remarkable SD of the macro-area slopes (0.64).

Table S10 and Figure S16 relate the universal "If the demonstrative determiner follows the noun, then the adjectives follows the noun" (Hawkins 1983: 82), which has high support (median main fixed effect is 2.20, median 95% CI 1.47 - 3.02, median BF = 88) and a high SD of the macro-area slopes (1.47). It might be the case that Australia deviates from the other macro-areas.

Table S11 and Figure S17 relate the universal "No language has a trial number unless it has a dual" (Greenberg 1963), which is not supported (median of the main fixed effect is 2.06, median

95% CI -1.96 - 5.21) and with a high SD of the macro-area slopes (1.73). This claim only holds in the Pacific.

Table S9: Median macro-area slope estimates from the spatiophylogenetic analyses of the universal "If a language has Prep word order, then if the adjective follows the noun, the relative clause follows the noun" (Hawkins 1983: 74), see Figure S15

macro-area	Estimate	Error	Q2.5	Q97.5
Africa	0.05	0.59	-1.11	1.44
Australia	-0.34	0.79	-2.47	0.76
Eurasia	0.13	0.60	-091	1.55
North America	0.20	0.75	-0.99	2.11
Papunesia	0.19	0.61	-0.84	1.82
South America	-0.06	0.71	-1.63	1.36

Table S10: Median macro-area slope estimates from the spatiophylogenetic analyses of the universal "If the demonstrative determiner follows the noun, then the adjectives follows the noun" (Hawkins 1983: 82), see Figure S16

macro-area	Estimate	Error	Q2.5	Q97.5
Africa	-0.66	0.55	-1.90	0.13
Australia	0.46	0.62	-0.43	2.01
Eurasia	0.26	0.44	-0.57	1.22
North America	0.09	0.49	-0.85	1.21
Papunesia	-0.004	0.40	-0.83	0.82
South America	0.07	0.50	-0.98	1.14

Table S11: Median macro-area slope estimates from the spatiophylogenetic analyses of the universal " No language has a trial number unless it has a dual" (Greenberg 1963), see Figure S17

macro-area	Error	Estimate	Q2.5	Q97.5
Africa	-0.38	1.83	-4.85	3.05
Australia	-0.45	1.78	-4.76	2.76
Eurasia	1.78	1.77	-5.01	2.33
North America	-0.39	1.81	-4.78	2.98
Papunesia	2.17	2.03	-0.48	6.81
South America	-0.41	1.80	-4.76	2.87

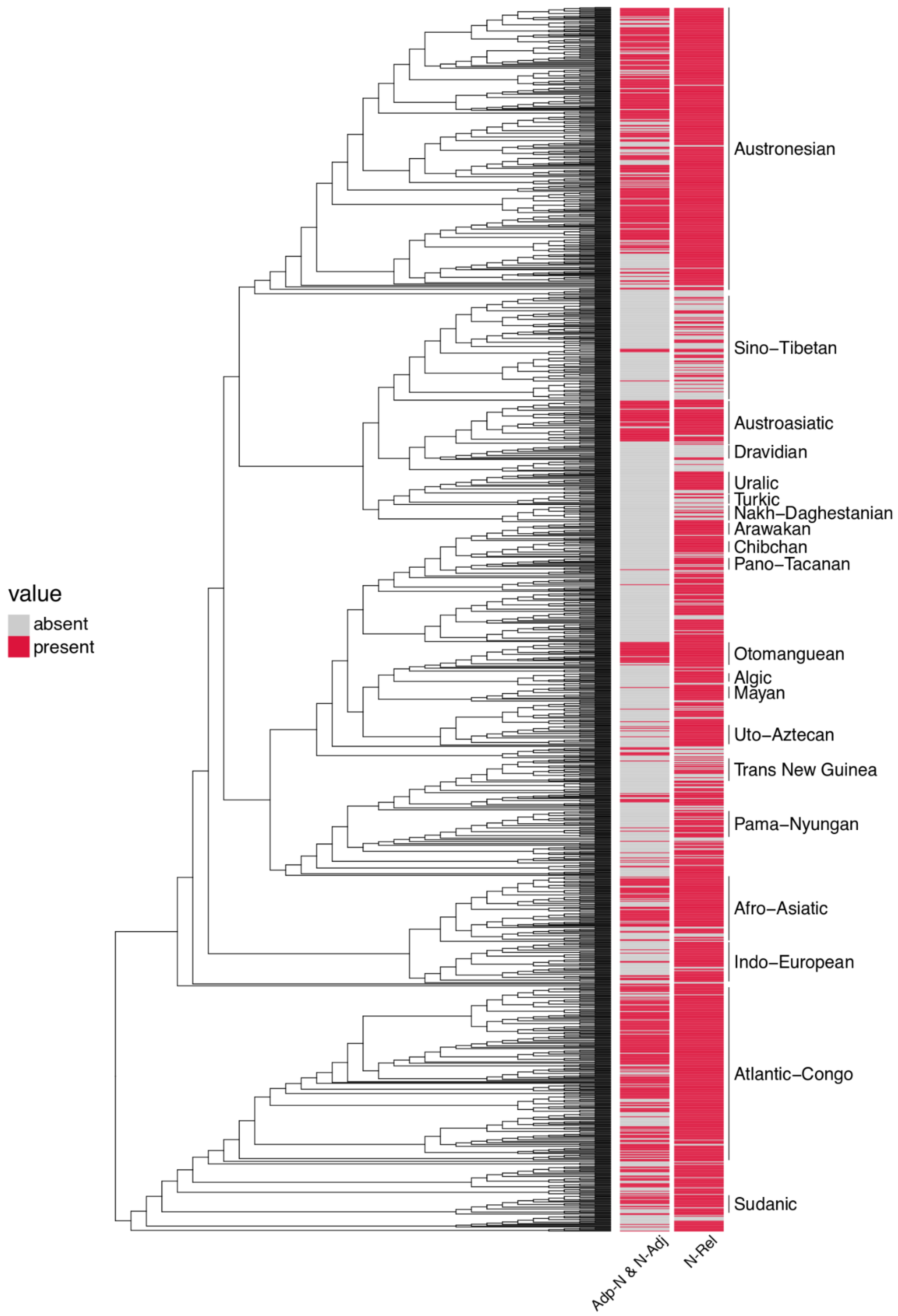

Figure S15: Dataset on adnominal word order from 1754 languages plotted onto the tips of the tips of the world tree, illustrating the universal "If a language has Prep word order, then if the adjective follows the noun, the relative clause follows the noun." (Hawkins 1983: 74). Note that the MCC tree is taken from a posterior sample of 902 trees, and support for higher order branches is extremely low.

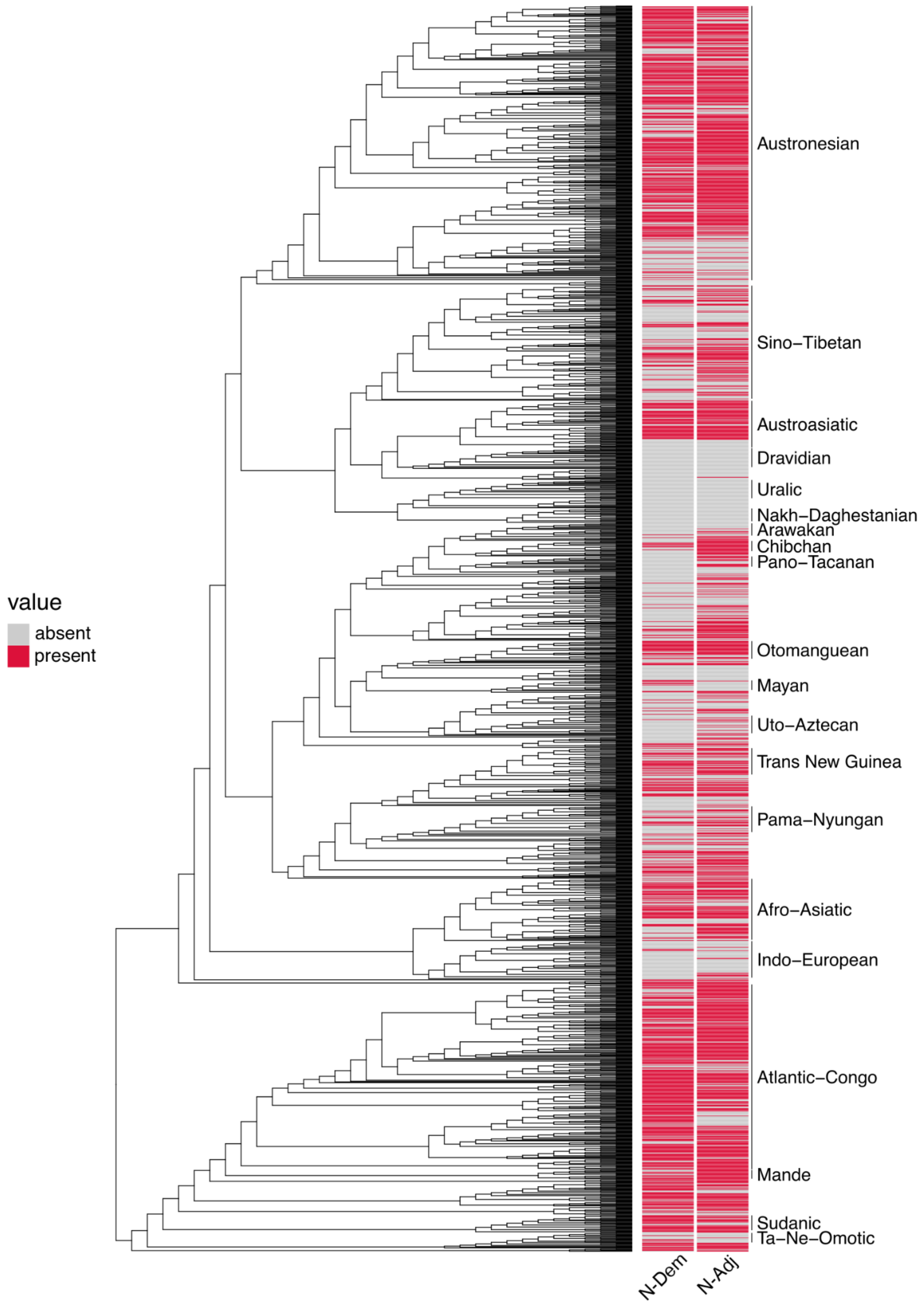

Figure S16: Dataset on adnominal word order from 1840 languages plotted onto the tips of the tips of the world tree, illustrating the universal "If the demonstrative determiner follows the noun, then the adjective follows the noun." (Hawkins 1983: 82). Note that the MCC tree is taken from a posterior sample of 902 trees, and support for higher order branches is extremely low.

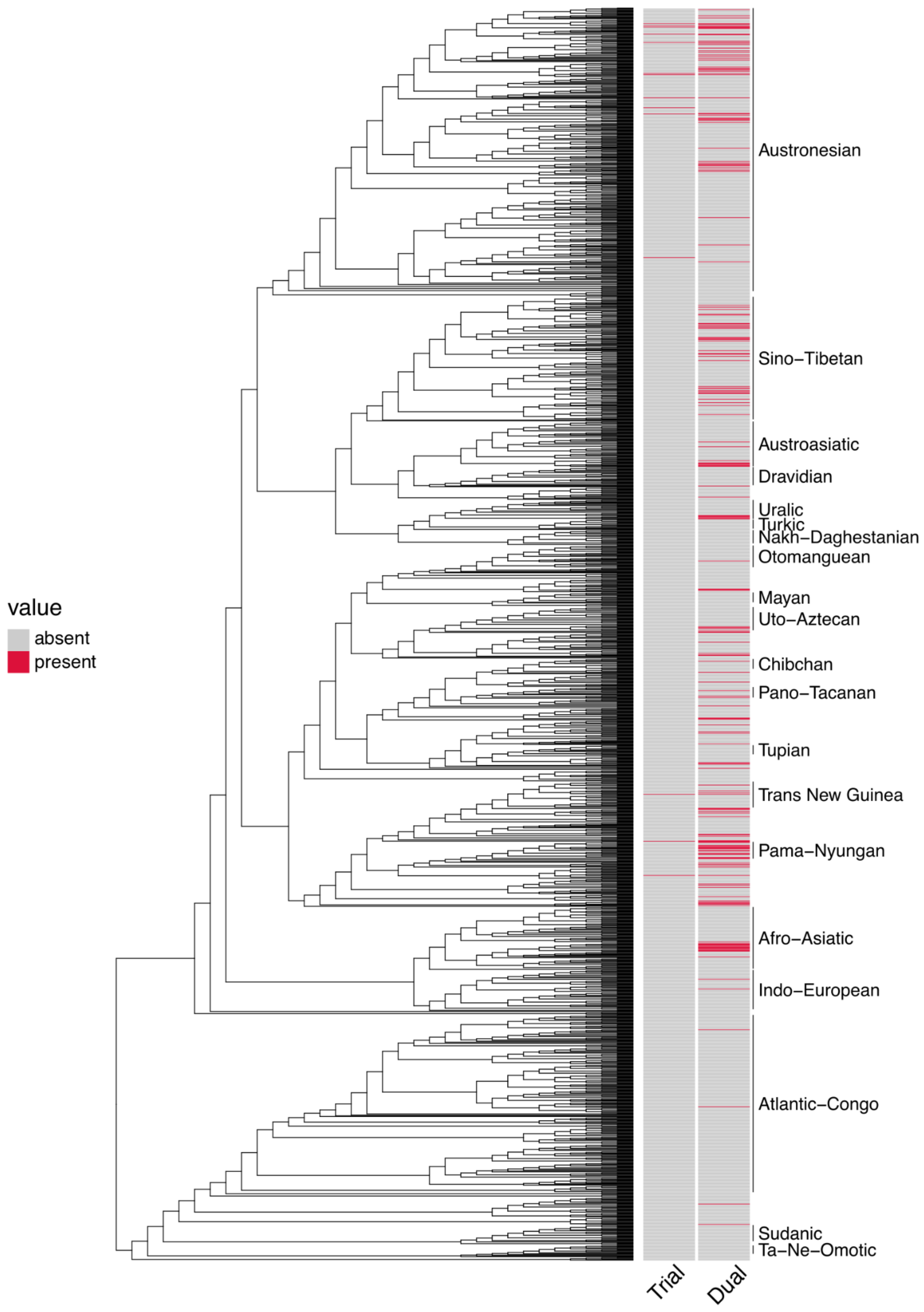

Figure S17: Dataset on the dual and trial from 1788 languages plotted onto the tips of the tips of the world tree, illustrating the universal "No language has a trial number unless it has a dual." (Greenberg 1963). Note that the MCC tree is taken from a posterior sample of 902 trees, and support for higher order branches is extremely low.

Supplementary Information: References

1. Skirgård, H., Haynie, H. J., Blasi, D. E., Hammarström, H., Collins, J., Latarche, J. J., Lesage, J., Weber, T., Witzlack-Makarevich, A., Passmore, S., Chira, A., Maurits, L., Dinnage, R., Dunn, M., Reesink, G., Singer, R., Bowern, C., Epps, P., Hill, J., … Gray, R. D. (2023). Grambank reveals the importance of genealogical constraints on linguistic diversity and highlights the impact of language loss. Science Advances, 9(16), eadg6175.
2. Bouckaert, Remco, David Redding, Oliver Sheehan, Thanos Kyritsis, Russell Gray, Kate E Jones, and Quentin Atkinson. 2022. 'Global Language Diversification Is Linked to Socio-Ecology and Threat Status'. Preprint. SocArXiv. https://doi.org/10.31235/osf.io/f8tr6.
3. Hammarström, Harald & Forkel, Robert & Haspelmath, Martin & Bank, Sebastian. 2022. Glottolog 4.6. Jena: Max Planck Institute for the Science of Human History. https://doi.org/10.5281/zenodo.3754591. (Available online at http://glottolog.org, Accessed on 2022-09-30.)
4. Plank, Frans and Elena Filimonova. 2006. 'The Universals Archive: A brief introduction for prospective users', STUF - Language Typology and Universals, vol. 53, no. 1, pp. 109–123.
5. Marchese, Lynell 1988. Noun classes and agreement systems in Kru: A historical approach. In Barlow & Ferguson (eds.), Agreement in Natural Language: Approaches, Theories, Descriptions, 323-342. Stanford: Center for the Study of Language and Information.
6. Greenberg, Joseph H. 1963. 'Some Universals of Grammar with Particular Reference to the Order of Meaningful Elements'. In Universals of Language, edited by Joseph H. Greenberg, 73–113. London: MIT Press.
7. Dryer, Matthew S. 1988. 'Object-Verb Order and Adjective-Noun Order: Dispelling a Myth'. Lingua 74: 185–217.
8. Hawkins, John A. 1983. Word Order Universals. San Diego: Academic Press.
9. Dryer, Matthew S. 1998. 'Why statistical universals are better than absolute universals.' Chicago Linguistic Society 33: The Panels, 1, 123–145.
10. Siewierska, Anna. 1996. 'Word Order Type and Alignment Type'. STUF - Language Typology and Universals 49 (2): 149–76.
11. Dryer, Matthew S. 2002. 'Case Distinctions, Rich Verb Agreement, and Word Order Type (Comments on Hawkins' Paper)'. Theoretical Linguistics 28(2).
12. Sinnemäki, Kaius. 2008. 'Complexity Trade-Offs in Core Argument Marking'. In Language Complexity: Typology, Contact, Change, edited by Matti Miestamo, Kaius Sinnemäki, and Fred Karlsson, 67–88. Amsterdam: Benjamins.
13. Aldai, Gontzal. 2011. 'Wh-Questions and SOV Languages in Hawkins' (2004) Theory: Evidence from Basque'. Linguistics 49 (5).
14. Schepper, Kees de. 2013. You and Me against the World?: First, Second and Third Person in the World's Languages. Utrecht: LOT.

15. Bickel, Balthasar, Alena Witzlack-Makarevich, and Taras Zakharko. 2015. ‘Typological Evidence against Universal Effects of Referential Scales on Case Alignment’. In Scales and Hierarchies, edited by Ina Bornkessel-Schlesewsky, Andrej L. Malchukov, and Marc Richards, 7–44. Berlin: De Gruyter.
16. Culbertson, Jennifer. 2017. ‘New Approaches to Greenbergian Word Order Dependencies’. In Dependencies in Language: On the Causal Ontology of Linguistic Systems, edited by Nicholas J. Enfield, 23–38. Berlin: Language Science Press.
17. Schmidtke-Bode, Karsten. 2019. ‘Attractor States and Diachronic Change in Hawkins's “Processing Typology”’. In Explanation in Typology: Diachronic Sources, Functional Motivations and the Nature of the Evidence, edited by Karsten Schmidtke-Bode, Natalia Levshina, Susanne Maria Michaelis, and Ilja A. Seržant, 123–48. Berlin: Language Science Press.
18. Tang, Marc, and One-Soon Her. 2019. ‘Insights on the Greenberg-Sanches-Slobin Generalization: Quantitative Typological Data on Classifiers and Plural Markers’. Folia Linguistica 53 (2): 297–331. https://doi.org/10.1515/flin-2019-2013.
19. Berg, Thomas. 2020. ‘Nominal and Pronominal Gender: Putting Greenberg's Universal 43 to the Test’. STUF - Language Typology and Universals 73 (4): 525–74.
20. Gerdes, Kim, Sylvain Kahane, and Xinying Chen. 2021. ‘Typometrics: From Implicational to Quantitative Universals in Word Order Typology’. Glossa: A Journal of General Linguistics 6 (1): 17. https://doi.org/10.5334/gjgl.764.
21. Guzmán Naranjo, Matías, and Laura Becker. 2022. ‘Statistical Bias Control in Typology’. Linguistic Typology 26 (3): 605–70.
22. Brosa-Rodríguez, A., Jiménez-López, M.D. 2023. A Typometrical Study of Greenberg's Linguistic Universal 1. In: Mehmood, R., et al. Distributed Computing and Artificial Intelligence, Special Sessions I, 20th International Conference. DCAI 2023. Springer, Cham.
23. Dryer, Matthew S. 1992. ‘The Greenbergian Word Order Correlations’. Language 68 (1): 81–138.
24. Dunn, Michael, Simon J Greenhill, Stephen C Levinson, and Russell D Gray. 2011. ‘Evolved Structure of Language Shows Lineage-Specific Trends in Word-Order Universals’. Nature 473 (7345): 79–82. https://doi.org/10.1038/nature09923.
25. Jäger, Gerhard, and Johannes Wahle. 2021. ‘Phylogenetic Typology’. Frontiers in Psychology 12 (July): 682132.
26. Lehmann, W. P. 1973. ‘A Structural Principle of Language and Its Implications’. Language 49 (1): 47.
27. Croft, William. 1988. ‘Agreement vs. Case Marking and Direct Objects’. In Agreement in Natural Language: Approaches, Theories, Descriptions, edited by Michael Barlow and Charles A. Ferguson, 159–79. Stanford: CSLI Publications.
28. Moravcsik, Edith A. 1974. ‘Object-Verb Agreement’. Working Papers on Language Universals 15: 25–140.

29. Greenberg, Joseph H., Charles E. Osgood, and James J. Jenkins. 1963. 'Memorandum Concerning Language Universals'. In Universals of Language, xv–xxvii. Cambridge, MA: MIT Press.
30. Hagège, Claude. 1982. 'Linguistic Universals as General Tendencies'. In Proceedings of the XIIIth International Congress of Linguists, August 29th – September 4th 1982, Tokyo, edited by Shirô Hattori and Kazuko Inoue, 36–940. Tokyo: Tokyo Press.
31. Stassen, Leon. 1992. 'A Hierarchy of Main Predicate Encoding'. In Meaning and Grammar. Cross-Linguistic Perspectives, edited by Michael Kefer and Johan van der Auwera, 179–201. Berlin: Mouton de Gruyter.
32. Kozinsky, Isaak. 1981. 'Nekotorye Grammaticeskie Universalii v Podsistemax Vyrazenija Subjektno-Objektnyx Otnnosenij [Some Grammatical Universals in Subsystems of Expression of Subject-Object Relations]'. Doctoral dissertation, Moscow: Moskovskij gosudarstvennyj universitet.
33. Campannella, Tommaso. 1638. Philosophiæ Rationalis Partes Quinque. Videlicet: Grammatica, Dialectica, Rhetorica, Poetica, Historiographia, Iuxta Propria Principia. Suorum Operum Toumus, Volume I. Paris: Ioannes du Bray.
34. Girard, Gabriel. 1747. Les Vrais Principles de La Langue Françoise, Ou La Parole Réduite En Méthode, Conformément Aux Lois de l'usage. En Seize Discours. Paris: Le Breton.
35. Smith, Adam. 1761 [1983]. 'Considerations Concerning the First Formation of Languages, and the Different Genius of Original and Compounded Languages'. In The Glasgow Edition of the Works and Correspondence of Adam Smith, Vol. IV: Lectures on Rhetoric and Belles Lettres, edited by J. C. Bryce, 201–26. Oxford: Clarendon Press.
36. Beauzée, Nicolas. 1767 [1974]. Grammaire Générale, Ou Exposition Raisonnée Des Éléments Nécessaires Du Langage, Pour Servir de Fondement à l'étude de Toutes Les Langues. Stuttgart: Frommann.
37. Palmaitis, Letas. 1978. 'Tipologines Pastabos Del Gimines Kategorijos Formavimosi. [Typological Notes on the Development of Gender]'. Baltistica 14 (1): 27–38.
38. Klimov, Georgij A. 1973. Ocerk Obscej Teorii Èrgativnosti. [Outline of a General Theory of Ergativity]. Moscow: NNauka.
39. Vardul', Ivan F. 1969. Jazykovye Universalii i Lingvisticeskaya Tipologia. [Language Universals and Linguistic Typology]. Moscow: Nauka.
40. Moravcsik, Edith A. 1994. 'Inflectional Morphology in the Hungarian Noun Phrase: A Typological Assessment'. EUROTYP Working Papers VII/22, 127–85.
41. Nichols, Johanna. 1992. Linguistic Diversity in Space and Time. Chicago: University of Chicago Press.
42. Rijkhoff, Jan. 1992. 'The Noun Phrase: A Typological Study of Its Form and Structure'. Doctoral dissertation, Universiteit van Amsterdam.

43. Ultan, Russell. 1978e. ‘Toward a Typology of Substantival Possession’. In Universals of Human Language Vol. 4: Syntax, edited by Joseph H. Greenberg, 11–50. Stanford: Stanford University Press.
44. Moravcsik, Edith A. 1997. ‘Parts and Wholes in the Hungarian Noun Phrase: A Typological Study’. In Proceedings of LP ’96: Typology: Prototypes, Item Orderings and Universals, edited by Bohumil Palek, 307–24. Prague: Charles University Press.
45. Cuny, A. 1906. Le nombre duel en grec. Paris: Klincksieck.
46. Renzi, Loeenzo. 1992. ‘Le développement de l’article en roman’. Revue Roumaine de Linguistique 37 (2–3): 161–76.
47. Gil, David. 1987. ‘Definiteness, Noun Phrase, Configurationality, and the Count-Mass Distinction’. In The Representation of (in)Definiteness, edited by Eric J. Reuland and Alice ter Meulen, 254–69. Cambridge, MA: MIT Press.
48. Haiman, John, and Pamela Munro, eds. 1983. Switch-Reference and Universal Grammar. Amsterdam: Benjamins.
49. Ultan, Russell. 1978c. ‘Interrogative Systems’. In Universals of Human Language Vol. 4: Syntax, edited by Joseph H. Greenberg, 211–48. Stanford: Stanford University Press.
50. Dryer, Matthew S. 1989. ‘Large Linguistic Areas and Language Sampling’. Studies in Language 13: 257–92.
51. Stassen, Leon. 1984. ‘The Comparative Compared’. Journal of Semantics 3: 143–82.
52. Stassen, Leon. 1997. Intransitive Predication. Oxford: Oxford University Press.
53. Bybee, Joan L. 1985. Morphology. A Study of the Relation between Meaning and Form. Amsterdam: Benjamins.
54. Plank, Frans. 1994. ‘What agrees with what in what, generally speaking?’, EUROTYP Working Papers, vol. VII, p. 20.
55. Kiparsky, Paul. 1997. ‘The Rise of Positional Licensing’. In Parameters of Morphosyntactic Change, edited by Ans Van Kemenade and Nigel Vincent, 460–94. Cambridge: Cambridge University Press.
56. Hawkins, John A. 1990. ‘A Parsing Theory of Word Order Universals’. Linguistic Inquiry 21 (2): 223–61.
57. Lehmann, Winifred P. 1978a. Syntactic Typology: Studies in the Phenomenology of Language. Austin: University of Texas Press.
58. Moravcsik, Edith A. 1969. ‘Determination’. Working Papers on Language Universals 1: 63–98.
59. Stolz, Thomas. 1992. Lokalkasussysteme: Aspekte Einer Strukturellen Dynamik. Wilhelmsfeld: Egert.
60. Schwartz, Arthur. 1972. ‘The VP-Constituent of SVO Languages’. Working Papers on Language Universals 8: 21–51.
61. Trask, Robert L. 1979. ‘On the Origins of Ergativity’. In Ergativity: Towards a Theory of Grammatical Relations, edited by Frans Plank, 385–404. London: Academic Press.

62. Subbarao, Karumuri V. 1997. 'Agreement in South Asian Languages'. In Keynote Address Delivered at International Dravidian Linguistics Association, Seminar on Agreement. Chennai, India.
63. Subbarao, Karumuri V. 1998. 'Linguistic Theory and Syntactic Typology: A Proposal for a Symbiotic Relationship'. In Vaagbhaarati: Proceedings of the International Conference on South Asian Languages, edited by Ljudmila V. Khokhlova and Atil Sawani, 5–23. Moscow: Institute of Asian and African Languages, Moscow State University.
64. Serebrennikov, Boris A. 1974. Verojatnostnye Obosnovanija v Komparativistike [Probability Foundations in Comparative Linguistics]. Moscow: Nauka.
65. Mahajan, Anoop. 1997. 'Universal Grammar and the Typology of Ergative Languages'. In Studies on Universal Grammar and Typological Variation, edited by Artemis Alexiadou and T. Allan Hall, 35–57. Amsterdam: Benjamins.
66. Keenan, Edward L. 1974. 'The Functional Principle: Generalizing the Notion of "Subject Of"'. Chicago Linguistic Socienty 10: 298–309.
67. Lord, Carol. 1993. Historical Change in Serial Verb Constructions. Amsterdam: Benjamins.
68. Payne, Doris L. 1990. The Pragmatics of Word Order: Typological Dimensions of Verb Initial Languages. Berlin: Mouton de Gruyter.
69. Bybee, Joan L., William Pagliuca, and Revere Perkins. 1990. 'On the Asymmetries in the Affixation of Grammatical Material'. In Studies in Typology and Diachrony: Papers Presented to Joseph H. Greenberg on His 75th Birthday, edited by William Croft, Keith Denning, and Suzanne Kemmer, 1–42. Amsterdam: Benjamins.
70. Keenan, Edward L. 1985. 'Relative Clauses'. In Language Typology and Syntactic Description, Volume 2: Complex Constructions, edited by Timothy Shopen, 141–70. Cambridge: Cambridge University Press.
71. Cole, Peter. 1987. 'The Structure of Internally Headed Relative Clauses'. Natural Language and Linguistic Theory 5: 277–302.
72. Foster, Joseph F., and Charles A. Hofling. 1987. 'Word Order, Case, and Agreement'. Linguistics 25 (3): 475-499. https://doi.org/10.1515/ling.1987.25.3.475.
73. Rijkhoff, Jan. 2000. 'When Can a Language Have Adjectives? An Implicational Universal'. In Approaches to the Typology of Word Classes, edited by Petra M. Vogel and Comrie, Bernard, 217–57. Berlin: Mouton de Gruyter.
74. Kalinina, Elena Ju. 1998. 'Nefinitnye Skazuemye v Nezavisimom Predlozenii. [Nonfinite Predicates in the Main Clause]'. Doctoral dissertation, Moscow: Moskovskij gosudarstvennyj universitet.
75. Kuno, Susumu. 1974. 'The Position of Relative Clauses and Conjunctions'. Linguistic Inquiry V (1): 117–36.
76. Cutler, Anne, John A Hawkins, and Gary Gilligan. 1985. 'The Suffixing Preference: A Processing Explanation'. Linguistics 23 (5): 723–58.

77. Hawkins, John A., and Gary Gilligan. 1988. ‘Prefixing and Suffixing Universals in Relation to Basic Word Order’. Lingua 74: 219–59.
78. Lehmann, Christian, and Edith A. Moravcsik. 2000. ‘Noun’. In Morphology: An International Handbook, edited by Geert Booij, Christian Lehmann, Joachim Mugdan, Wolfgang Kesselheim, and Stavros Skopeteas, 732–56. Berlin: Walter de Gruyter.
79. Givón, Talmy. 1971. ‘Historical Syntax and Synchronic Morphology: An Archaeologist’s Field Trip’. In The Diachrony of Grammar, 1:1–26. Amsterdam: Benjamins.
80. Schmidt, P. Wilhelm. 1926. Die Sprachfamilien Und Sprachenkreise Der Erde. Heidelberg: Winter.
81. Aikhenvald, Alexandra Y., and Robert M. W. Dixon. 1998. ‘Dependencies between Grammatical Systems’. Language 74 (1): 56–80.
82. Dryer, Matthew S. 2013. Determining Dominant Word Order. In: Dryer, Matthew S. & Haspelmath, Martin (eds.) WALS Online. Leipzig: MaxPlanck Institute for Evolutionary Anthropology.
83. Bürkner, Paul. 2017. ‘brms: An R Package for Bayesian Multilevel Models Using Stan’, Journal of Statistical Software, vol. 80, no. 1, pp. 1–28.
84. Pagel, M. (1994). Detecting correlated evolution on phylogenies: A general method for the comparative analysis of discrete characters. Proceedings of the Royal Society (B), 255, 37–45.
85. Pagel, M., & Meade, A. (2006). Bayesian analysis of correlated evolution of discrete characters by reversible-jump Markov Chain Monte Carlo. The American Naturalist, 167(6), 808–825.
86. R Core Team 2023. R: A language and environment for statistical computing. R Foundation for Statistical Computing, Vienna, Austria. https://www.R-project.org/. Version 4.3.0.
87. Carpenter et al. 2017. ‘Stan : A Probabilistic Programming Language’, J. Stat. Soft., vol. 76, no. 1, pp. 1–32.
88. Bürkner, Paul. (2023). Estimating Phylogenetic Multilevel Models with brms. Online resource available at: https://cran.r-project.org/web/packages/brms/vignettes/brms_phylogenetics.html.
89. Sinnemäki, Kaius. 2010. ‘Word Order in Zero-Marking Languages’, Studies in Language 34 (4): pp. 869–912.
90. Claessens, S., Kyritsis, T., & Atkinson, Q. D. (2020). Revised analysis shows relational mobility predicts sacrificial behavior in Footbridge but not Switch or Loop trolley problems. Proceedings of the National Academy of Sciences, 117(24), 13203–13204.
91. Guzmán Naranjo, M., & Becker, L. (2022). Statistical bias control in typology. Linguistic Typology, 26(3), 605–670. https://doi.org/10.1515/lingty-2021-0002.
92. Shcherbakova, O., Michaelis, S. M., Haynie, H. J., Passmore, S., Gast, V., Gray, R. D., Greenhill, S. J., Blasi, D. E., & Skirgård, H. (2023). Societies of strangers do not speak less complex languages. Science Advances, 9(33), eadf7704.

93. Ribeiro, P. J., Diggle, P., Christensen, O., Schlather, M., Bivand, R., & Ripley, B. 2024. Package 'geoR': Analysis of Geostatistical Data [Computer software]. https://cran.r-project.org/web/packages/geoR/geoR.pdf.
94. Dinnage, R., Skeels, A., & Cardillo, M. (2020). Spatiophylogenetic modelling of extinction risk reveals evolutionary distinctiveness and brief flowering period as threats in a hotspot plant genus. Proceedings of the Royal Society B: Biological Sciences, 287(1926), 20192817.
95. Nychka, D., Furrer, R., Paige, J., Sain, S., & Nychka, M. D. (2015). Package 'fields' [Computer software]. http://cran. r-project. org/web/packages/fields/fields.
96. Bentz, C., & Winter, B. (2013). Languages with More Second Language Learners Tend to Lose Nominal Case. Language Dynamics and Change, 3(1), 1–27.
97. Coupé, C. (2018). Modeling linguistic variables with regression models: Addressing non-gaussian distributions, non-independent observations, and non-linear predictors with random effects and generalized additive models for location, scale and shape. Frontiers in Psychology, 9, 513.
98. Sinnemäki, K., & Di Garbo, F. (2018). Language Structures May Adapt to the Sociolinguistic Environment, but It Matters What and How You Count: A Typological Study of Verbal and Nominal Complexity. Frontiers in Psychology, 9, 89–23.
99. Josserand, M., Meeussen, E., Majid, A., & Dediu, D. (2021). Environment and culture shape both the colour lexicon and the genetics of colour perception. Scientific Reports, 11(1), 19095. https://doi.org/10.1038/s41598-021-98550-3.
100. Dediu, D. (2023). Ultraviolet light affects the color vocabulary: Evidence from 834 languages. Frontiers in Psychology, 14, 1143283.
101. Pagel, M., & Meade, A. (2024). BayesTraits V4.1.1. Software manual.
102. Xie, W., Lewis, P. O., Fan, Y., Kuo, L., & Chen, M. H. (2011). Improving marginal likelihood estimation for Bayesian phylogenetic model selection. Systematic Biology, 60(2), 150–160. https://doi.org/10.1093/sysbio/syq085.
103. Raftery, A. E. (1996). Hypothesis testing and model selection via posterior simulation. In W. R. Gilks, S. Richardson, & D. J. Spiegelhalter (Eds.), Markov Chain Monte Carlo in practice (pp. 163–188). Springer.
104. Croft, William. 2003. Typology and Universals. Cambridge: Cambridge University Press.
105. Cysouw, M. (2005). Quantitative methods in typology = Quantitative Methoden in der Typologie. In R. Köhler, G. Altmann, & R. G. Piotrowski (Eds.), Quantitative Linguistik: Ein internationales Handbuch (pp. 554–578). Walter de Gruyter.
106. Ladd, D. R., Roberts, S. G., and Dediu, D. (2014). Correlational Studies in Typological and Historical Linguistics. *Annual Review of Linguistics*, 1(1), 140804162027000. https://doi.org/10.1146/annurev-linguist-030514-124819.
107. Bromham, L., Hua, X., Cardillo, M., Schneemann, H., and Greenhill S.J. (2018) Parasites and politics: why cross-cultural studies must control for relatedness, proximity

and covariation. *Royal Society Open Science* 5: 181100. http://dx.doi.org/10.1098/rsos.181100

108. Hartmann, F. (2022). Methodological Problems in Quantitative Research on Environmental Effects in Phonology. *Journal of Language Evolution*, 7(1), 95–119. https://doi.org/10.1093/jole/lzac003.
109. Bromham, L. (2023) Meaning and purpose: Using phylogenies to investigate human history and cultural evolution. *Biological Theory*, 18(4), 284-302. https://doi.org/10.1007/s13752-022-00401-5.
110. Bromham, L. and Yaxley, K.J. (2023) Neighbours and relatives: Accounting for spatial distribution when testing causal hypotheses in cultural evolution. *Evolutionary Human Sciences*, 5:e27. doi:10.1017/ehs.2023.23.
111. Shcherbakova, O., Michaelis, S. M., Haynie, H. J., Passmore, S., Gast, V., Gray, R. D., Greenhill, S. J., Blasi, D. E., & Skirgård, H. (2023). Societies of strangers do not speak less complex languages. Science Advances, 9(33), eadf7704. https://doi.org/10.1126/sciadv.adf7704.
112. Cysouw, M. (2011). Understanding transition probabilities. *Linguistic Typology*, 15(2). https://doi.org/10.1515/lity.2011.028.
113. Cathcart, C. A. (2018). Modeling linguistic evolution: A look under the hood. *Linguistics Vanguard*, 4(1). https://doi.org/10.1515/lingvan-2017-0043.
114. Aissen, Judith. 2003. Differential Object Marking: Iconicity vs. Economy. Natural Language & Linguistic Theory 21, 435–483.
115. Haude, Katharina and Witzlack-Makarevich, Alena. "Referential hierarchies and alignment: An overview" Linguistics, vol. 54, no. 3, 2016, pp. 433-441.
116. Silverstein, Michael. 1976. Hierarchy of features and ergativity. In Robert M. W. Dixon (ed.), Grammatical categories in Australian languages, 112–171. Atlantic Highlands, NJ: Humanities Press.
117. Dixon, R. M. W. 1979. Ergativity. Language 55. 59–138.
118. Corbett, Greville G. 1979. 'The agreement hierarchy', J. Ling., vol. 15, no. 2, pp. 203–224.
119. Corbett, Greville G. 2022. 'The Agreement Hierarchy Revisited: The Typology of Controllers'. Word Structure 15 (3): 181–225.
120. Noyer, Rolf. 1992. Features, Positions, and Affixes in Autonomous Morphological Structures. Ph.D. Dissertation. Cambridge, Massachusetts: Massachusetts Institute of Technology.
121. Harley, Heidi, and Elizabeth Ritter. 2002. Person and Number in Pronouns: A Feature-Geometric Analysis. Language 78(3), 482-526.
122. Corbett, Greville G. 2000. Number. Cambridge: Cambridge University Press.
123. Miestamo, Matti. 2009. Implicational hierarchies and grammatical complexity. In Geoffrey Sampson, David Gil, and Peter Trudgill (eds.). Language Complexity as an

Evolving Variable (Studies in the Evolution of Language 13). Oxford: Oxford University Press, 80-97.

124. Franzon, Francesca, Chiara Zanini, and Rosa Rugani. 2019. Do non-verbal number systems shape grammar? Numerical cognition and Number morphology compared. Mind & Language 34(1), 37-58.

125. Steele, Susan. 1978. 'Word Order Variation: A Typological Study'. In Universals of Human Language Vol 4: Syntax, edited by Joseph H. Greenberg, 585–623. Stanford: Stanford University Press.

126. Dryer, Matthew S. 2009. 'The Branching Direction Theory of Word Order Correlations Revisited'. In Universals of Language Today, edited by Sergio Scalise, Elisabetta Magni, and Antonietta Bisetto, 76:185–207. Studies in Natural Language and Linguistic Theory. Dordrecht: Springer Netherlands.

127. Dryer, Matthew S. 2011. 'The Evidence for Word Order Correlations'. Linguistic Typology 15 (2).

128. Hawkins, John A. 1994. A Performance Theory of Order and Constituency. Cambridge: Cambridge University Press.

129. Hartung, K., Jäger, G., Gröttrup, S., & Georges, M. (2022). Typological Word Order Correlations with Logistic Brownian Motion. Proceedings of the 4th Workshop on Research in Computational Linguistic Typology and Multilingual NLP, 22–26. https://doi.org/10.18653/v1/2022.sigtyp-1.

130. Dryer, M. S., and Haspelmath, M. (2013). The World Atlas of Language Structures Online. Leipzig: Max Planck Institute for Evolutionary Anthropology. Available online at: http://wals.info/.

131. Hammarström, H., Castermans, T., Forkel, R., Verbeek, K., Westenberg, M. A., & Speckmann, B. (2018). Simultaneous visualization of language endangerment and language description. Language Documentation & Conservation, 12, 359-392. Retrieved from http://hdl.handle.net/10125/24792.

132. Cysouw, M. (2010). Dealing with diversity: Towards an explanation of NP-internal word order frequencies. Linguistic Typology, 14(2–3). https://doi.org/10.1515/lity.2010.010.

133. Dryer, M. S. (2018). On the order of demonstrative, numeral, adjective, and noun. Language, 94(4), 798–833. https://doi.org/10.1353/lan.2018.0054.

134. Dryer, M. S. (2019). Grammaticalization accounts of word order correlations. In *Explanation in typology: Diachronic sources, functional motivations, and the nature of the evidence*, edited by K. Schmidtke-Bode, N. Levshina, S. M. Michaelis, and I. A. Seržant. (pp. 63–95). Berlin: Language Science Press.

135. Futrell, R., Levy, R. P., & Gibson, E. (2020). Dependency locality as an explanatory principle for word order. Language, 96(2), 371–412. https://doi.org/10.1353/lan.2020.0024.

136. Gerdes, K., Kahane, S., & Chen, X. (2019). Rediscovering Greenberg's Word Order Universals in UD. Proceedings of the Third Workshop on Universal Dependencies (UDW, SyntaxFest 2019), 124–131. https://doi.org/10.18653/v1/W19-801.
137. Hahn, M., Jurafsky, D., & Futrell, R. (2020). Universals of word order reflect optimization of grammars for efficient communication. Proceedings of the National Academy of Sciences, 117(5), 2347–2353. https://doi.org/10.1073/pnas.191092311.
138. Hahn, M., & Xu, Y. (2022). Crosslinguistic word order variation reflects evolutionary pressures of dependency and information locality. Proceedings of the National Academy of Sciences, 119(24), e2122604119. https://doi.org/10.1073/pnas.2122604119.
139. Hawkins, J. A. (2014). *Word order universals* (Vol. 3). Amsterdam: Elsevier.
140. Jing, Y., Widmer, P., & Bickel, B. (2021). Word Order Variation is Partially Constrained by Syntactic Complexity. Cognitive Science, 45(11). https://doi.org/10.1111/cogs.13056.
141. Good, J., ed. (2008). *Linguistic universals and language change*. Oxford: Oxford University Press.
142. Baker, M. C. (2001). *The atoms of language*. Oxford: Oxford University Press.
143. Biberauer, T., & Roberts, I. (2005). Changing EPP parameters in the history of English: Accounting for variation and change. English Language and Linguistics, 9(1), 5–46. https://doi.org/10.1017/S1360674305001528.
144. Biberauer, T, and Sheehan, M., eds. (2013). *Theoretical approaches to disharmonic word order.* Oxford: Oxford University Press.
145. Cinque, G. (2005). Deriving Greenberg's Universal 20 and Its Exceptions. Linguistic Inquiry, 36(3), 315–332.
146. Cinque, G. (2013). Cognition, universal grammar, and typological generalizations. Lingua, 130, 50–65. https://doi.org/10.1016/j.lingua.2012.10.007.
147. Cinque, G. (2014). *Typological studies: Word order and relative clauses*. London: Routledge.
148. Kayne, R. S. (1994). *The antisymmetry of syntax* (Vol. 25). Cambridge, MA: MIT press.
149. Sheehan, M., Biberauer, T., Roberts, I., & Holmberg, A. (2017). *The final-over-final condition: A syntactic universal*. Cambridge, MA: MIT Press.
150. Svenonius, P., ed. (2000). *The Derivation of VO and OV.* Amsterdam: Benjamins.
151. Svenonius, P. (2025). Word Order Universals and Their Relationship to Structure. Annual Review of Linguistics, 11(1), 137–162.

152. Whitman, J. (2008). The classification of constituent order generalizations and diachronic explanation. In *Linguistic universals and language change*, edited by J. Good. (pp. 233–252). Oxford: Oxford University Press.

153. Vennemann, T. (1974) "Theoretical Word Order Studies: Results and Problems." Papiere zur Linguistik 7, 5-25.

154. Keenan, E. L. and Comrie, B. (1977). Noun Phrase Accessibility and Universal Grammar, Linguistic Inquiry, vol. 8, no. 1, 63–99.

155. Hawkins, J. A. (2004). *Efficiency and complexity in grammars.* Oxford: Oxford University Press.

156. Culbertson, J., Franck, J., Braquet, G., Barrera Navarro, M., & Arnon, I. (2020a). A learning bias for word order harmony: Evidence from speakers of non-harmonic languages. Cognition, 204, 104392. https://doi.org/10.1016/j.cognition.2020.104392.

157. Martin, A., Adger, D., Abels, K., Kanampiu, P., & Culbertson, J. (2024). A Universal Cognitive Bias in Word Order: Evidence From Speakers Whose Language Goes Against It. Psychological Science, 35(3), 304-311.

158. Bybee, J. L. (1988). The diachronic dimension in explanation. *Explaining language universals*, *35*, 379.

159. Lehmann, C. (1992). Word order change by grammaticalization. In *Internal and External Factors in Syntactic Change*, edited by M. Gerritsen and D. Stein. Berlin: De Gruyter Mouton. https://doi.org/10.1515/9783110886047.395

160. LaPolla, R. J. (2002). Problems of methodology and explanation in word order universals research. Languages and Cultures of the East, 1–18.

161. Kuteva, T., & Heine, B. (2008). On the explanatory value of grammaticalization. In *Linguistic universals and language change*, edited by J. Good. (pp. 215–230). Oxford: Oxford University Press.

162. Aristar, A. R. (1991). On Diachronic Sources and Synchronic Pattern: An Investigation into the Origin of Linguistic Universals, Language, vol. 67, no. 1, 1–33.

163. Collins, J. (2019). Some language universals are historical accidents. In *Explanation in typology: Diachronic sources, functional motivations, and the nature of the evidence*, edited by K. Schmidke-Bode, N. Levshina, S. M. Michaelis, and I. A. Seržant. (pp. 47–61). Berlin: Language Science Press.

164. Marchello-Nizia, C. (2009). Word Order from Latin to French: A Case of Grammaticalization (from Pragmatics to Syntactics) or of Typological Coherence? In *Grammaticalization and Pragmatics: Facts, Approaches, Theoretical Issues*, edited by C. Rossari, C. Ricci, and A. Spiridon. Studies in Pragmatics, Volume: 5. Leiden: Brill

165. Cristofaro, S. (2014). Competing motivation models and diachrony: What evidence for what motivations? In *Competing motivations in grammar and usage*, edited by B. MacWhinney, A. L. Malchukov, and E. Moravcsik. (pp. 282–298). Oxford: Oxford University Press.

166. Cristofaro, S. (2017). Implicational universals and dependencies. In *Dependencies in language*, edited by N. J. Enfield. (pp. 9–23). Berlin: Language Science Press.

167. Cristofaro, S. (2019). Taking diachronic evidence seriously: Result-oriented vs. Source- oriented explanations of typological universals. In *Explanation in typology: Diachronic sources, functional motivations and the nature of the evidence*, edited by K. Schmidke-Bode, N. Levshina, S. M. Michaelis, and I. A. Seržant. (pp. 25–46). Language Science Press.

168. Harris, A. C. (2008). On the Explanation of Typologically Unusual Structures. In *Linguistic Universals and Language Change*, edited by J. Good. (pp. 54–76). Oxford: Oxford University Press.

169. Mansfield, J. & Krapp, L. S. A Simple Explanation for Harmonic Word Order. Cognitive Science 49, e70056 (2025).

170. Nichols, J. (2008). Universals and diachrony: Some observations. In *Linguistic Universals and Language Change*, edited by J. Good. (pp. 287–293). Oxford: Oxford University Press.

171. Tily, H., & Jaeger, T. F. (2011). Complementing quantitative typology with behavioral approaches: Evidence for typological universals. Linguistic Typology, 15(2). https://doi.org/10.1515/lity.2011.033.

172. Gray, Louis H. (1939). Foundations of Language. New York: Macmillan.

173. Jensen, Hans (1952). Die sprachliche Kategorie des Numerus.Wissenschaftliche Zeitung der Universität Rostock, Reihe Gesellschafts und Sprachwissenschaften 1:2, 1-21.

174. Haspelmath, M. (2009). The best-supported language universals refer to scalar patterns deriving from processing cost. Behavioral and Brain Sciences, 32(5), 457–458. doi:10.1017/S0140525X09990689.

175. MacWhinney, B., Malchukov, A. L., & Edith Moravcsik (Eds.). (2014). *Competing Motivations in Grammar and Usage*. Oxford: Oxford University Press.

176. Kirby, S. (1997). Competing motivations and emergence: Explaining implicational hierarchies. Linguistic Typology, 1(1). https://doi.org/10.1515/lity.1997.1.1.5.

177. Kirby, S. (1999). *Function, selection, and innateness.* Oxford: Oxford University Press.

178. Fábregas, A. (2013). Differential object marking in Spanish: State of the art. Borealis–An International Journal of Hispanic Linguistics, 2(2), 1-80.

179. Haimain, J. (1983). Iconic and economic motivation. Language, 59(4), 781–819.

180. Harley, H. and Ritter, E., (2002). Person and number in pronouns: A feature-geometric analysis. Language, 78(3), 482-526.

181. Harbour, D. (2014). Paucity, abundance and the theory of number. Language, 90(1), 185-229.
182. Harbour, D. (2016). *Impossible persons.* Cambridge, MA: MIT Press.
183. Toosarvandani, M. (2023). The interpretation and grammatical representation of animacy. Language 99(4), 760-808. https://dx.doi.org/10.1353/lan.2023.a914193.
184. Sigurðsson, H. A. (2004). The syntax of person, tense, and speech features. Rivista di Linguistica - Italian Journal of Linguistics, 16(1), 219-251.
185. Haspelmath, M. (2008a). Creating economical morphosyntactic patterns in language change. In *Language universals and language change*, edited by J. Good. (pp. 185–214). Oxford: Oxford University Press.
186. Haspelmath, M. (2008b). Frequency vs. iconicity in explaining grammatical asymmetries. Cognitive Linguistics, 19(1), 1–33. https://doi.org/10.1515/COG.2008.001.
187. Cristofaro, S., & Zúñiga, F., eds. (2018a). *Typological hierarchies in synchrony and diachrony*. Amsterdam: Benjamins.
188. Cristofaro, S., & Zúñiga, F. (2018b). Synchronic vs. Diachronic approaches to typological hierarchies. In *Typological hierarchies in synchrony and diachrony*, edited by S. Cristofaro and F. Zúñiga. (pp. 3–30). Amsterdam: Benjamins.
189. Givón, T. (1979). *On understanding grammar*. New York: Academic Press.
190. DuBois, J., Kumpf, L. E., & Ashby, W. J. (2003). *Preferred Argument Structure: Grammar as architecture for function*. Amsterdam: Benjamins.
191. Enger, H.-O., & Nesset, T. (2011). Constraints on diachronic development: The Animacy Hierarchy and the Relevance Constraint. STUF - Language Typology and Universals, 64(3), 193–212. https://doi.org/10.1524/stuf.2011.0015.
192. Kuno, S., & Etsuko Kaburaki. (1977). Empathy and Syntax. Linguistic Inquiry, 8(4), 627–672. http://www.jstor.org/stable/4178011.
193. Boswijk, V., & Coler, M. (2020). What is Salience? Open Linguistics, 6(1), 713–722. https://doi.org/10.1515/opli-2020-0042
194. Culbertson, J., Schouwstra M., & S. Kirby, S. (2020b). From the world to word order: Deriving biases in noun phrase order from statistical properties of the world. Language, 96(3), 696–717.
195. Bybee, J. L. (2010). Markedness: Iconicity, economy, and frequency. In *The Oxford Handbook of Linguistic Typology*, edited by J. J. Song. (pp. 131-147). Oxford: Oxford University Press.
196. Gildea, S., & Zúñiga, F. (2016). Referential hierarchies: A new look at some historical and typological patterns. Linguistics, 54(3), 483–529. https://doi.org/10.1515/ling-2016-0007.
197. Cristofaro, Sonia. (2012). Typological universals in synchrony and diachrony: the evolution of number marking. Talk at SLE 2012, Stockholm, Sweden.

198. Kiparsky, P. (2008). Universals constrain change; change results in typological generalizations. In *Linguistic universals and language change*, edited by J. Good. (pp. 23–53). Oxford: Oxford University Press.

199. Moravcsik, Edith A. (1993). Government. In *Syntax: Ein internationales Handbuch zeitgenössischer Forschung Vol. 1*, edited by J. Jacobs, A. v. Stechow, W. Sternefeld & T. Vennemann. (pp. 707- 721). Berlin: de Gruyter.

200. Hurford, J. & Kirby, S. (1995). Neural preconditions for proto-language. Behavioral and Brain Sciences 18, 193–194.

201. Campanella, T. (1638). *Philosophiæ rationalis partes quinque. Videlicet: Grammatica, dialectica, rhetorica, poetica, historiographia, iuxta propria principia. Suorum operum toumus, Volume I*, Paris: Ioannes du Bray.

202. Uspensky, B. A. (1965). *Strukturnaja tipologija jazykov*. [Structural Typology of Languages.] Moskva: Nauka.

203. Bobaljik, J. D. (2012). *Universals in Comparative Morphology: Suppletion, Superlatives, and the Structure of Words*. Cambridge, MA: MIT Press.

204. Baker, M. (2015). *Case*. Cambridge, MA: Cambridge University Press.

205. Mithun, M. (2003). Why prefixes? Acta Linguistica Hungarica, 50(1–2), 155–185.

206. Jacques, G. (2013). Harmonization and disharmonization of affix ordering and basic word order. Linguistic Typology, 17(2). https://doi.org/10.1515/lity-2013-0009.

207. Koplenig, A., Meyer, P., Wolfer, S., & Müller-Spitzer, C. (2017). The statistical trade-off between word order and word structure – Large-scale evidence for the principle of least effort. PLoS ONE, 12(3), e0173614-25. https://doi.org/10.1371/journal.pone.0173614.

208. Nijs, J., Van De Velde, F., & Cuyckens, H. (2025). Is Word Order Responsive to Morphology? Disentangling Cause and Effect in Morphosyntactic Change in Five Western European Languages. Entropy, 27(1), 53. https://doi.org/10.3390/e27010053.

209. Lehmann, Winfred P. (1978b). Conclusions: The profound unity underlying languages. In *Syntactic Typology: Studies in the Phenomenology of Language*, edited by Lehmann, W. P. (pp. 395-432). Austin: University of Texas Press.

210. Siewierska, A. & Bakker, D. (1996). The distribution of subject and object agreement and word order type. Studies in Language 20(1), 115-161.

211. Gibson, E., Piantadosi, S. P., Brink, K., Bergen, L., Lim, E. & Saxe, R. (2013). A noisy-channel account of crosslinguistic word-order variation. Psychological science 24(7), 1079–1088.

212. Futrell, R., Mahowald, K. & Gibson, E. (2015). Quantifying word order freedom in dependency corpora. In *Proceedings of the third international conference on dependency linguistics* edited by Depling. (pp. 91–100).

213. Sinnemäki, K. (2014). Complexity trade-offs: A case study. In *Measuring grammatical complexity*, edited by F. J. Newmeyer & L. B. Preston. (pp. 179–201). Oxford: Oxford University Press.
214. Levshina, N. (2019). Token-based typology and word order entropy: A study based on Universal Dependencies. Linguistic Typology, 23(3), 533–572. https://doi.org/10.1515/lingty-2019-0025.
215. Shcherbakova, O., Blasi, D. E., Gast, V., Skirgård, H., Gray, R. D. & Greenhill, S. J. (2024). The evolutionary dynamics of how languages signal who does what to whom. Scientific Reports 14(1). 7259.
216. Polinsky, M., & Magyar, L. (2020). Headedness and the Lexicon: The Case of Verb-to-Noun Ratios. Languages, 5(1), 9. https://doi.org/10.3390/languages5010009.
217. Kulikov, L. (2006). Case systems in a diachronic perspective. In *Case, valency and transitivity*, edited by L. Kulikov, A. Malchukov, & P. de Swart. (pp. 23–48). Amsterdam: John Benjamins.
218. Hawkins, J. A. (2002). Symmetries and asymmetries: Their grammar, typology and parsing. Theoretical Linguistics, 28(2). https://doi.org/10.1515/thli.2002.28.2.95.
219. Berdicevskis, A., Schmidtke-Bode, K., & Seržant, I. (2020). Subjects tend to be coded only once: Corpus-based and grammar-based evidence for an efficiency-driven trade-off. Proceedings of the 19th International Workshop on Treebanks and Linguistic Theories, 79–92.
220. Birchall, J. (2014). Verbal argument marking patterns in South American languages. In The Native Languages of South America: Origins, Development, Typology, edited by L. O'Connor & P. Muysken. (pp. 223–249). Cambridge: Cambridge University Press.
221. Grossman, E., & Polis, S. (2018). Swimming against the typological tide or paddling along with language change?: Dispreferred structures and diachronic biases in affix ordering. Journal of Historical Linguistics, 8(3), 388–443. https://doi.org/10.1075/jhl.17027.gro.
222. Nichols, J. (1986). Head-Marking and Dependent-Marking Grammar. Language, 62(1), 56–119.
223. Cysouw, M. (2002). Interpreting typological clusters. Linguistic Typology, 6(1). https://doi.org/10.1515/lity.2002.003.
224. Bickel, B., & Nichols, J. (2007). Inflectional morphology. In Linguistic typology and syntactic description. Volume III: Grammatical categories and the lexicon, edited by T. Shopen. (2nd ed., pp. 169–240). Cambridge: Cambridge University Press.
225. Lupyan, G. & Dale, R. (2010). Language Structure Is Partly Determined by Social Structure. PLoS ONE 5(1): e8559. https://doi.org/10.1371/journal.pone.0008559.
226. Sinnemäki, K. (2020). Linguistic system and sociolinguistic environment as competing factors in linguistic variation: A typological approach. Journal of Historical Linguistics 6(2), pp. 20191010. https://doi.org/10.1515/jhsl-2019-1010.

227. Nichols, J. (1988). On alienable and inalienable possession. In In Honor of Mary Haas: From the Haas Festival Conference on Native American Linguistics, edited by W. Shipley. (pp. 557-610). Berlin: Mouton de Gruyter.
228. Hawkins, J. A. (1979). Implicational Universals as Predictors of Word Order Change. Language, 55(3), 618–648.
229. Haspelmath, M. (2024). Different explanations in grammar are mutually compatible: Structural, evolutionary and biocognitive. Typology of Morphosyntactic Parameters, 7, 133–151.
230. Maslova, E. (2000). A dynamic approach to the verification of distributional universals. Linguistic Typology, 4(3), 307-333. https://doi.org/10.1515/lity.2000.4.3.307.
231. Moravcsik, E. A. (2007). What is universal about typology? Linguistic Typology, 11(1), 27-41. https://doi.org/10.1515/LINGTY.2007.004.
232. Bybee, J. (2006). Language change and universals. In *Linguistic universals*, edited by R. Mairal & J. Gil. (pp. 179–194). Cambridge: Cambridge University Press.
233. Bybee, J. (2015). *Language change*. Cambridge: Cambridge University Press.
234. Bickel, B. (2008). A general method for the statistical evaluation of typological distributions. Manuscript, University of Leipzig.
235. Bickel, B. (2013). Distributional Biases in Language Families. In *Language Typology and Historical Contingency: In Honor of Johanna Nichols*, edited by B. Bickel, L. A. Grenoble, D. A. Peterson, and A. Timberlake. (pp. 415–44). Amsterdam: John Benjamins.
236. Bickel, B. (2015). Distributional Typology: Statistical Inquiries into the Dynamics of Linguistic Diversity. In *The Oxford Handbook of Linguistic Analysis, 2nd Edition*, edited by B. Heine and H. Narrog. (pp. 901–23). Oxford: Oxford University Press.
237. Cysouw, M. (2010). Dealing with Diversity: Towards an Explanation of NP-Internal Word Order Frequencies. *Linguistic Typology* 14(2–3), 221–34.
238. Bakker, P., Daval-Markussen. A., Parkvall, M. & Plag, I. (2011). Creoles are typologically distinct from non-creoles. Journal of Pidgin and Creole Languages 26(1), 5–42.
239. Bates, Douglas, Martin Mächler, Ben Bolker, and Steve Walker. 2015. 'Fitting Linear Mixed-Effects Models Using Lme4'. Journal of Statistical Software 67 (1).
240. R Core Team (2021). R: A language and environment for statistical computing. R Foundation for Statistical Computing, Vienna, Austria. https://www.R-project.org/.
241. Kuznetsova, Alexandra, Per B. Brockhoff, and Rune H. B. Christensen. (2017). 'LmerTest Package: Tests in Linear Mixed Effects Models'. Journal of Statistical Software 82 (13). https://doi.org/10.18637/jss.v082.i13.
