# Supplementary material for: Enduring constraints on grammar revealed by Bayesian spatiophylogenetic analyses
Source: Nat Hum Behav. 2025 Nov 17;10(1):126–36. doi: 10.1038/s41562-025-02325-z (PMC12846912; doi:10.1038/s41562-025-02325-z)
Supplement: Supplementary file 2 — Reporting Summary [file 41562_2025_2325_MOESM2_ESM.pdf]

nature portfolio

Corresponding author(s): *Double-anonymous peer review submissions: write DAPR and your manuscript number here instead of author names.*

Last updated by author(s): *YYYY-MM-DD*

Reporting Summary

Nature Portfolio wishes to improve the reproducibility of the work that we publish. This form provides structure for consistency and transparency in reporting. For further information on Nature Portfolio policies, see our Editorial Policies and the Editorial Policy Checklist.

Statistics

For all statistical analyses, confirm that the following items are present in the figure legend, table legend, main text, or Methods section.

n/a	Confirmed	
☐	☒	The exact sample size (n) for each experimental group/condition, given as a discrete number and unit of measurement
☐	☒	A statement on whether measurements were taken from distinct samples or whether the same sample was measured repeatedly
☐	☒	The statistical test(s) used AND whether they are one- or two-sided<br>*Only common tests should be described solely by name; describe more complex techniques in the Methods section.*
☐	☒	A description of all covariates tested
☐	☒	A description of any assumptions or corrections, such as tests of normality and adjustment for multiple comparisons
☐	☒	A full description of the statistical parameters including central tendency (e.g. means) or other basic estimates (e.g. regression coefficient) AND variation (e.g. standard deviation) or associated estimates of uncertainty (e.g. confidence intervals)
☐	☒	For null hypothesis testing, the test statistic (e.g. F, t, r) with confidence intervals, effect sizes, degrees of freedom and P value noted<br>*Give P values as exact values whenever suitable.*
☐	☒	For Bayesian analysis, information on the choice of priors and Markov chain Monte Carlo settings
☐	☒	For hierarchical and complex designs, identification of the appropriate level for tests and full reporting of outcomes
☐	☒	Estimates of effect sizes (e.g. Cohen's d, Pearson's r), indicating how they were calculated

Our web collection on statistics for biologists contains articles on many of the points above.

Software and code

Policy information about availability of computer code

Data collection

No code was used to create primary data, of course, but (base) R (version: 4.3.0) was used to manipulate datafiles, such as merge datafiles, import information from Grambank, create appropriate datafiles for BayesTraits, etc.

Data that was taken from existing datasets:
Glottolog: version 4.3
Grambank: version 1.0

Data analysis	All code for analysis we used is open source. For R we used packages:<br>brms (R version: 4.3.2, package version: 2.20.3) which depends on STAN (R version: 4.3.2, package version: 2.33.0);<br>geoR (R version: 4.3.2, package version: 1.9-2);<br>fields (R version: 4.3.2, package version: 15.2);<br>ape (R version: 4.3.2, package version: 5.7-1);<br>corHMM (R version: 4.4.0, package version: 2.8);<br>treeio (R version: 4.4.0, package version: 1.24.3);<br>ggtree (R version: 4.4.0, package version: 3.11.0);<br>lme4 (R version: 4.3.0, package version: 1.1-34);<br>lmerTest (R version: 4.3.0, package version: 3.1-3)<br><br>We used BayesTraits version 4.1.2 (compiled with OpenMP support).

For manuscripts utilizing custom algorithms or software that are central to the research but not yet described in published literature, software must be made available to editors and reviewers. We strongly encourage code deposition in a community repository (e.g. GitHub). See the Nature Portfolio guidelines for submitting code & software for further information.

Data

Policy information about availability of data

All manuscripts must include a data availability statement. This statement should provide the following information, where applicable:

- Accession codes, unique identifiers, or web links for publicly available datasets
- A description of any restrictions on data availability
- For clinical datasets or third party data, please ensure that the statement adheres to our policy

All the data that support the findings of this study are available via GitHub at https://github.com/SimonGreenhill/TestingLinguisticUniversals

Research involving human participants, their data, or biological material

Policy information about studies with human participants or human data. See also policy information about sex, gender (identity/presentation), and sexual orientation and race, ethnicity and racism.

Reporting on sex and gender	N/A
Reporting on race, ethnicity, or other socially relevant groupings	N/A
Population characteristics	N/A
Recruitment	N/A
Ethics oversight	N/A

Note that full information on the approval of the study protocol must also be provided in the manuscript.

Field-specific reporting

Please select the one below that is the best fit for your research. If you are not sure, read the appropriate sections before making your selection.

☐ Life sciences ☒ Behavioural & social sciences ☐ Ecological, evolutionary & environmental sciences

For a reference copy of the document with all sections, see nature.com/documents/nr-reporting-summary-flat.pdf

Behavioural & social sciences study design

All studies must disclose on these points even when the disclosure is negative.

Study description	Our study is closest to an evolutionary study design. We use a comprehensive new global grammatical database (Grambank) to test 191 proposed grammatical universals. The data in Grambank ultimately rely on linguistic analyses made by experts on the language and typologists. Using Bayesian phylogenetic methods we test these putative universals while controlling for spatial and phylogenetic non-independence. Here we conduct four distinct experiments: 1) uncontrolled GLMM models; 2) spatiophylogenetic GLMM models 3) GLMM models with a categorial control for language family; 4) BayesTraits/overt evolutionary modeling without correcting for spatial autocorrelation.
Research sample	Here we sample human languages and universals. Regarding the latter, we evaluated over 2,000 documented universals in the Universals Archive (Plank and Filimonova 2006, https://typo.uni-konstanz.de/rara/category/universals-archive/). We compared the morphosyntactic features involved in these universals to the data in Grambank version 1.0 (Skirgård et al. 2023) to determine which

	of them could be analyzed using features from Grambank. Then we reformulated those universals that were testable, first by splitting complex universals that address multiple ostensibly associated linguistic features into simple implicational generalizations (which we refer to here as simply 'universals' or 'generalizations'). Second, we matched feature constellations from generalizations to Grambank questionnaire questions and combinations thereof. Since Grambank only covers morphosyntax, any generalizations involving phonology or semantics were excluded; many other claims were excluded because they were too specific, were explicitly diachronic, or were not implicational. From 146 original universals that matched the Grambank variables and our criteria for synchronic implicational claims, we formulated 191 simple universals to test. Regarding languages, we review the language sample in SI Sect. 1. As for sample coverage per family and per area, we refer to the Supplementary Information of Skirgård et al. (2023). The overall sampling in Grambank across areas can be assessed in Figure S2 of their paper, which shows the coverage over macro-areas (as defined in Glottolog, Hammarström et al. 2022). The coverage over macro-areas is even, all areas have between 24% and 34% coverage. Grambank version 1.0 covers between 13% and 68% of the 15 largest language families as listed on Glottolog (Hammarström et al. 2022). Coverage for Austroasiatic, Isolates (as a group), and Pama-Nyungan is fair, while Indo-European and Tai-Kadai languages are underrepresented.
Sampling strategy	For both universals and languages, a maximally exhaustative sampling strategy was chosen. We include all universals that are in the Universals Archive (see above) and that are testable using Grambank. We include all languages that have the necessary information in Grambank to test the universals on.
Data collection	See above. The core authors responsible for data collection are Hannah Haynie, Olena Shcherbakova, Hedvig Skirgård, and Annemarie Verkerk.
Timing	Data collection was performed between June 2019 and June 2021, intermittedly given involvements in other projects by all parties involved.
Data exclusions	See above on excluding universals that could not be studied using Grambank data.
Non-participation	We did not have any (human) participants.
Randomization	Randomization is irrelevant for this research.

Reporting for specific materials, systems and methods

We require information from authors about some types of materials, experimental systems and methods used in many studies. Here, indicate whether each material, system or method listed is relevant to your study. If you are not sure if a list item applies to your research, read the appropriate section before selecting a response.

Materials & experimental systems

n/a	Involved in the study
☒	☐ Antibodies
☒	☐ Eukaryotic cell lines
☒	☐ Palaeontology and archaeology
☒	☐ Animals and other organisms
☒	☐ Clinical data
☒	☐ Dual use research of concern
☒	☐ Plants

Methods

n/a	Involved in the study
☒	☐ ChIP-seq
☒	☐ Flow cytometry
☒	☐ MRI-based neuroimaging

Plants

Seed stocks	N/A
Novel plant genotypes	N/A
Authentication	N/A
